# Supplementary material for: Study protocol: The effect of a low-carbohydrate enteral nutrition formula on postoperative hyperglycemia in non-diabetic patients with esophageal cancer: A randomized exploratory phase II trial (ENLICHE study)
Source: PLoS One. 2025 May 28;20(5):e0325039. doi: 10.1371/journal.pone.0325039 (PMC12118858; doi:10.1371/journal.pone.0325039)
Supplement: S1 Study Protocol — (DOCX) [file pone.0325039.s001.docx]

Study Protocol

A Randomized Exploratory phase II Trial evaluating the Effect of Enteral Nutrition formula with Low Carbohydrate on Postoperative Hyperglycemia in Non-Diabetic Patients with Esophageal Cancer

(ENLICHE study)

**Principal investigator:**

**Masayuki Watanabe**

Department of Gastroenterological Surgery, Cancer Institute Hospital, Japanese Foundation for Cancer Research

〒135-8550

3-8-31 Ariake, Koto-ku, Tokyo

TEL：03-3520-0111

FAX：03-3570-0343

E-mail：masayuki.watanabe@jfcr.or.jp

**Research Secretariat:**

**Yu Imamura**

Department of Gastroenterological Surgery, Cancer Institute Hospital, Japanese Foundation for Cancer Research

〒135-8550

3-8-31 Ariake, Koto-ku, Tokyo

TEL：03-3520-0111

FAX：03-3570-0343

Email:[yu.imamura@jfcr.or.jp](mailto:yu.imamura@jfcr.or.jp)

**Masayoshi Terayama**

Department of Gastroenterological Surgery, Cancer Institute Hospital, Japanese Foundation for Cancer Research

〒135-8550

3-8-31 Ariake, Koto-ku, Tokyo

TEL：03-3520-0111

FAX：03-3570-0343

E-mail：masayoshi.terayama@jfcr.or.jp

# 0. Overview

## 0.1. Shema


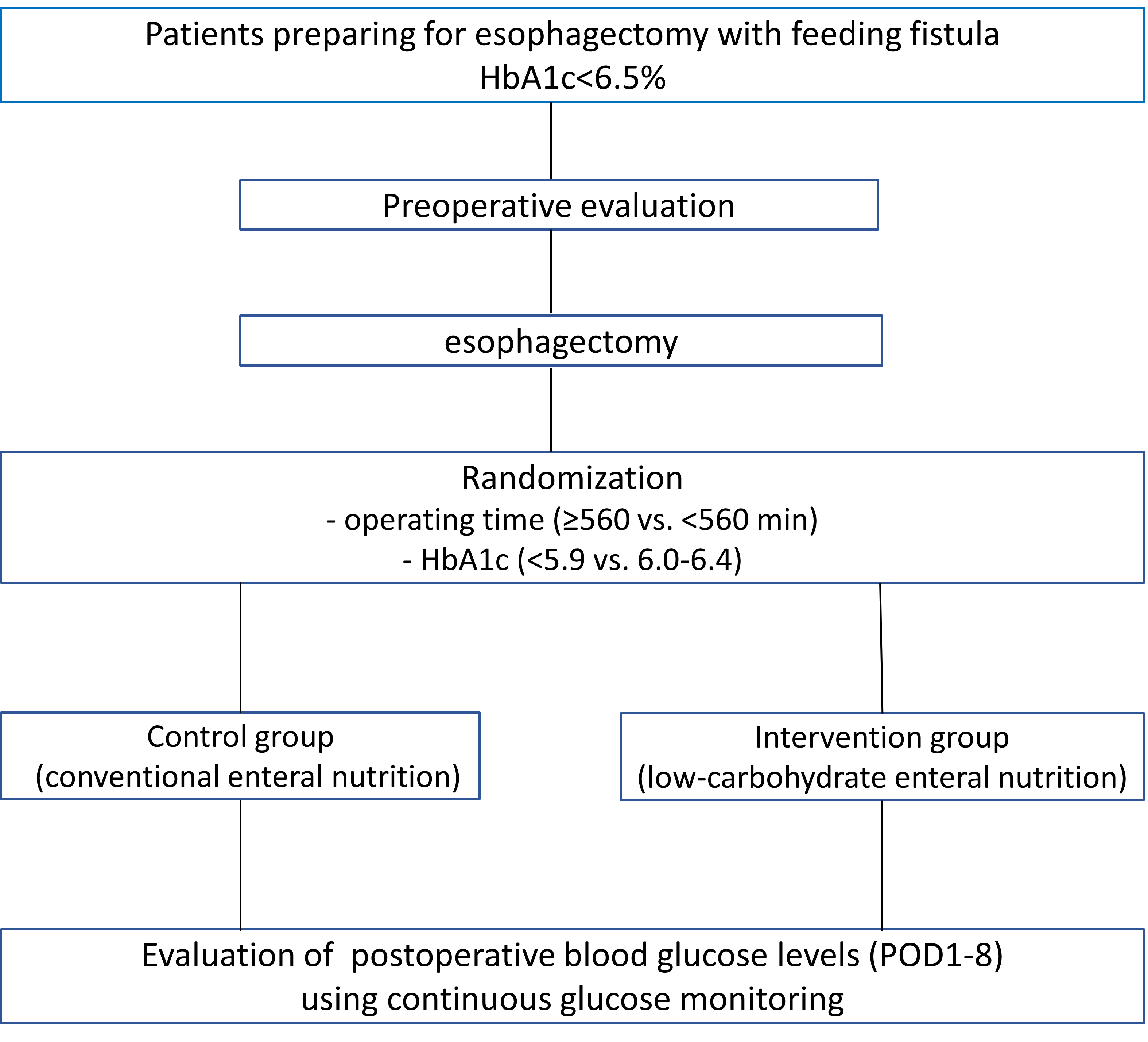


## 0.2. Objectives

This study aims to evaluate the effectiveness of the glucose control enteral formula Glucerna®-REX (Abbott Japan) in suppressing hyperglycemia when used as a postoperative enteral nutrition formulation for esophageal cancer patients, in comparison to a standard enteral nutrition formula without glucose restriction (Meiji Main)

**Primary endpoint**:

The average value of Time in Range (TIR) measured by Continuous Glucose Monitoring (CGM) up to the second postoperative day.

**Secondary endpoints**:

1. Incidence rate of infectious complications during hospitalization

2. Incidence rate of infectious complications within 30 days postoperatively

3. Incidence rate of all complications during hospitalization

4. Incidence rate of adverse events

5. Average value of Time Above Range (TAR) during the entire measurement period

(postoperative days 1-8) measured by CGM

6. Daily TAR values measured by CGM up to postoperative day 8

7. Average value of Area Under the Curve (AUC) during the entire measurement period

(postoperative days 1-8) measured by CGM

8. Daily AUC values measured by CGM up to postoperative day 8

9. Average value of Time in Range (TIR) during the entire measurement period (postoperative days 1-8) measured by CGM

10. Daily TIR values measured by CGM up to postoperative day 8

11. Rate of change in nutritional indicators during hospitalization compared to the time of admission (serum albumin, prealbumin, and total protein levels)

12. Number of cases requiring blood glucose control intervention for hyperglycemia (≥ 300 mg/dL)

13. Number of cases requiring changes to enteral nutrition formulas from postoperative day 3 onwards

14. Number of cases requiring a reduction of more than 50% in enteral nutrition from the previous day after postoperative day 3

## 0.3. Eligibility

### Patients who meet all of the inclusion criteria and do not meet any of the exclusion criteria will be included.

### 0.3.1. Inclusion criteria

1. Diagnosed histologically with esophageal cancer. Histological type is not specified..
2. Do not have a history of diabetes mellitus (HbA1c less than 6.5 at first visit).
3. The scheduled surgery is subtotal esophagectomy with gastric tube reconstruction.
4. Be at least 20 years of age at the time of registration.
5. Sufficient explanation of the study has been provided, and informed consent for

participation has been obtained.

1. Organ function is preserved, and the patient is tolerant to general anesthesia.

### 0.3.2. Exclusion Criteria

1. Patients with distant metastases.
2. Patients who have undergone palliative resection.
3. Patients who have undergone second stage reconstruction.
4. Patients who underwent a total pharyngeal laryngeal esophagectomy.
5. Patients who required combined thoracic ductectomy.
6. Patients who have undergone preoperative chemoradiotherapy.
7. Patients who did not receive steroid prophylaxis for unavoidable reasons.
8. Patients with implanted medical devices such as pacemakers.

## 0.4. Treatment

From postoperative day 1, the control group will receive the standard enteral formula, Meiji Main, while the intervention group will receive the glucose-restricted enteral formula, Glucerna®-REX. The formula will be continuously administered for 24 hours at a rate of 400 mL on postoperative day 1, 800 mL on day 2, 1200 mL on day 3, and 1600 mL from day 4 onwards. From postoperative days 4 to 8, the administration will be maintained at 1600 mL. On postoperative day 9, both groups will switch to standard postoperative management, transitioning to a concentrated enteral formula, HineX® E-gel, at 1000 mL, and start oral intake.

## 0.5. Planned number of enrollments and duration of the study

Number of registered patients: 50 (25 patients in the control group and 25 patients in the

intervention group).

Registration period: 1.5 years after jRCT disclosure.

Follow-up period: 0.5 years after the end of enrollment

Analysis period: 1 year after the end of enrollment

Total study period: 2.5 years

## 0.6. Inquiries

[Contact information for patient registration and inquiries regarding patient

selection criteria]

**Yu Imamura**

Department of Gastroenterological Surgery, Cancer Institute Hospital, Japanese Foundation for Cancer Research

〒135-8550

3-8-31 Ariake, Koto-ku, Tokyo

TEL：03-3520-0111

FAX：03-3570-0343

Email:[yu.imamura@jfcr.or.jp](mailto:yu.imamura@jfcr.or.jp)

**Masayoshi Terayama**

Department of Gastroenterological Surgery, Cancer Institute Hospital, Japanese Foundation for Cancer Research

〒135-8550

3-8-31 Ariake, Koto-ku, Tokyo

TEL：03-3520-0111

FAX：03-3570-0343

E-mail：masayoshi.terayama@jfcr.or.jp

[Inquiries that do not have clinical judgment such as EDC operation]

**Data Center: Yoshiko Matsui**

Advanced Cancer Treatment Development Center, Planning and Strategy Department, Cancer Institute Hospital, Japanese Foundation for Cancer Research

〒135-8550 3-8-31 Ariake, Koto-ku, Tokyo

TEL：03-3520-0111 FAX:：03-3570-0701

E-mail：yoshiko.matsui@jfcr.or.jp

# Contents

[0. Overview 3](#_Toc161155319)

[0.1. Shema 3](#_Toc161155320)

[0.2. Objective 3](#_Toc161155321)

[0.3. Eligibility 4](#_Toc161155322)

[0.3.1. Inclusion criteria 4](#_Toc161155323)

[0.3.2. Exclusion Criteria 4](#_Toc161155324)

[0.4. Treatment 4](#_Toc161155325)

[0.5. Planned number of enrollments and duration of the study 4](#_Toc161155326)

[0.6. Inquiries 4](#_Toc161155327)

[Contents 6](#_Toc161155328)

[1. Objectives 9](#_Toc161155329)

[2. Background and Rationale for the Test Plan 9](#_Toc161155330)

[2.1. Background 9](#_Toc161155331)

[2.2. Current status of our department 10](#_Toc161155332)

[2.2.1 Enteral Nutrition 10](#_Toc161155333)

[2.2.2. Blood Glucose Monitoring and Management 10](#_Toc161155334)

[2.3. Overview of the Study Intervention 11](#_Toc161155335)

[2.3.1. Enteral Nutrition 11](#_Toc161155336)

[2.3.2. Ingredients of enteral nutrition formula used in this study 11](#_Toc161155337)

[2.3.3. FreeStyleLibre Pro 12](#_Toc161155338)

[2.4. Study Design 12](#_Toc161155339)

[2.4.1. Blood glucose measurement using CGM 12](#_Toc161155340)

[2.4.2. Clinical Hypothesis 13](#_Toc161155341)

[2.4.3. Prospects for Patient Enrollment 13](#_Toc161155342)

[2.5. Expected benefits and disadvantages associated with participation 14](#_Toc161155343)

[2.5.1. Expected Benefits 14](#_Toc161155344)

[2.5.2. Expected Burden and Disadvantages 14](#_Toc161155345)

[2.6. Significance of the Study 14](#_Toc161155346)

[2.7. Ancillary Research 14](#_Toc161155347)

[3. Criteria and Definitions Used in the Exam 14](#_Toc161155348)

[3.1. Infectious Complications 14](#_Toc161155349)

[3.2 Diagnostic Criteria for Esophageal Cancer 14](#_Toc161155350)

[4. Patient Eligibility Criteria 14](#_Toc161155351)

[4.1. Inclusion criteria 15](#_Toc161155352)

[4.2. Exclusion Criteria 15](#_Toc161155353)

[5. Registration and Allocation 15](#_Toc161155354)

[5.1. Registration Procedure 15](#_Toc161155355)

[5.1.1. Notes on registration 16](#_Toc161155356)

[5.2. Randomization and Randomization Factors 16](#_Toc161155357)

[6. Treatment plan and Criteria for Treatment Modification 17](#_Toc161155358)

[6.1. Protocol Treatment 17](#_Toc161155359)

[6.1.1. Enteral Nutrition Used: 17](#_Toc161155360)

[6.1.2. Dosing Schedule, Dosage and Dosage 17](#_Toc161155361)

[6.1.3. Blood glucose management 17](#_Toc161155362)

[6.2. Criteria for Protocol Treatment Completion, Discontinuation, or Modification 18](#_Toc161155363)

[6.2.1. Definition of Protocol Treatment Completion 18](#_Toc161155364)

[6.2.2. Criteria for Discontinuation of Protocol Treatment 18](#_Toc161155365)

[6.2.3. Change Criteria for Protocol Treatment 18](#_Toc161155366)

[6.3. Combined treatment and supportive treatment 18](#_Toc161155367)

[6.3.1. Acceptable Concomitant and Supportive Therapy for Hyperglycemia and Hypoglycemia 18](#_Toc161155368)

[6.3.2. Other Acceptable Concomitant and Supportive Cares 19](#_Toc161155369)

[6.3.2. Unacceptable Concomitant and Supportive Therapy 19](#_Toc161155370)

[7. Anticipated Adverse Events 19](#_Toc161155371)

[7.1. Expected Adverse Events and Defects 19](#_Toc161155372)

[7.1.1. Adverse Events Associated with Enteral Nutrition (Meiji Main and Glucerna-REX ®) 19](#_Toc161155373)

[7.1.2. Adverse Events Associated with Esophagectomy 19](#_Toc161155374)

[7.1.3. Adverse Events Associated with the Use of FreeStyle Libre Pro 19](#_Toc161155375)

[7.1.4. Malfunctions Associated with the Use of FreeStyle Libre Pro 20](#_Toc161155376)

[7.2. Evaluation of Adverse Events / Adverse Reactions 20](#_Toc161155377)

[7.3. Adverse Event Observation Period 20](#_Toc161155378)

[7.4. Causality Assessment 20](#_Toc161155379)

[8. Evaluation items 20](#_Toc161155380)

[8.1. Endpoints prior to enrollment (within 28 days prior to the date of surgery) 20](#_Toc161155381)

[8.2. Evaluation items on the day of surgery 21](#_Toc161155382)

[8.3. Evaluation Items on Postoperative Day 1 21](#_Toc161155383)

[8.4. Evaluation Items on Postoperative Days 2 to 3 21](#_Toc161155384)

[8.5. Evaluation Items on Postoperative Days 4 to 8 21](#_Toc161155385)

[8.6. Evaluation Items on Postoperative Day 9 22](#_Toc161155386)

[8.7. Evaluation Items at Discharge: Acceptable discharge date±7 days 22](#_Toc161155387)

[8.8. Endpoints 30 days after the last dose of Meiji Main and Glucerna-REX ® (post-observation): acceptable + 14 days 22](#_Toc161155388)

[8.9. Study Calendar 23](#_Toc161155389)

[9. Reporting of Adverse Events 25](#_Toc161155390)

[9.1. Adverse Events Subject to Reporting Requirements 25](#_Toc161155391)

[9.2. Reporting Requirements and Reporting Procedures in the Event of Adverse Events or Illnesses 26](#_Toc161155392)

[9.2.1. Responsibilities of Persons Engaged in the Study 26](#_Toc161155393)

[9.2.2. Responsibilities of the Research Office 26](#_Toc161155394)

[9.2.3. Responsibilities of the Principal Investigator and the Research Secretariat 26](#_Toc161155395)

[9.2.4. Report to the Efficacy and Safety Evaluation Committee 26](#_Toc161155396)

[9.2.5. Responsibilities of the Efficacy and Safety Evaluation Committee 26](#_Toc161155397)

[9.2.6. Report to the Accredited Clinical Research Review Board and the Minister of Health, Labour and Welfare 26](#_Toc161155398)

[9.2.7. Periodic Reporting 26](#_Toc161155399)

[10. Determination and endpoint definition 27](#_Toc161155400)

[10.1. Evaluation items 27](#_Toc161155401)

[11. Statistical matters 27](#_Toc161155402)

[11.1. Definition of Analysis Populations](#_Toc161155403)

[27](#_Toc161155403)

[11.2. Handling of Missing Data 28](#_Toc161155404)

[11.3. Main Analysis 28](#_Toc161155405)

[11.4. Sub-evaluation projects (efficacy) 28](#_Toc161155406)

[11.5. Sub-secondary evaluation (security) 28](#_Toc161155407)

[11.6. Planned Registrations 29](#_Toc161155408)

[11.7. Interim Analysis 29](#_Toc161155409)

[11.8. Intentional level and multiplicity 29](#_Toc161155410)

[11.9. Final Analysis 29](#_Toc161155411)

[12. Discontinuation of the entire study 29](#_Toc161155412)

[12.1. Overall Study Discontinuation Criteria 29](#_Toc161155413)

[12.2. Procedures for Discontinuation of Clinical Research 30](#_Toc161155414)

[13. Ethical matters 30](#_Toc161155415)

[13.1. Patient Protection 30](#_Toc161155416)

[13.2. Informed Consent 30](#_Toc161155417)

[13.2.1. Explanation to Patients 30](#_Toc161155418)

[13.2.2. consent 31](#_Toc161155419)

[13.2.3. Withdrawal of consent 31](#_Toc161155420)

[13.3. Personal Information 32](#_Toc161155421)

[13.3.1. Policies, Laws, and Norms to Follow 32](#_Toc161155422)

[13.3.2. Protection of Personal Information and Patient Identification 32](#_Toc161155423)

[13.3.3. Purpose of use of personal information, items to be used, and method of use 32](#_Toc161155424)

[13.4. Source Materials 32](#_Toc161155425)

[13.5. Storage of Samples and Information 33](#_Toc161155426)

[13.6. Indemnification 33](#_Toc161155427)

[13.7. Intellectual Property 33](#_Toc161155428)

[13.8. Protocol Compliance 33](#_Toc161155429)

[13.9. Application to CRB and Notification of Implementation Plan 33](#_Toc161155430)

[14. Periodic Reporting 33](#_Toc161155431)

[15. Management of Conflicts of Interest (COI) of Persons Involved in Clinical Research 34](#_Toc161155432)

[16. Changes to the contents of documents approved by the CRB 34](#_Toc161155433)

[16.1 Changes to the Implementation Plan 34](#_Toc161155434)

[16.2 Changes to the Protocol 34](#_Toc161155435)

[16.3 Changes to the Consent Brief and Explanation and Re-Consent of the Patient 34](#_Toc161155436)

[16.4 Other Approved Documents 35](#_Toc161155437)

[17. Management of nonconformances 35](#_Toc161155438)

[17.1. Non-Conformances 35](#_Toc161155439)

[17.2. Material Non-Conformities 35](#_Toc161155440)

[17.3. Not suitable for reporting 35](#_Toc161155441)

[18. Monitoring and Auditing 36](#_Toc161155442)

[18.1. Periodic Monitoring 36](#_Toc161155443)

[18.2. Auditing 36](#_Toc161155444)

[19. Termination of the entire study 36](#_Toc161155445)

[19.1. Termination of Study 36](#_Toc161155446)

[19.2. Procedure for Termination of Research 36](#_Toc161155447)

[19.3. Handling of Research Results 37](#_Toc161155448)

[19.3.1. Publication of Research Results 37](#_Toc161155449)

[19.3.2. Secondary Use of Data 37](#_Toc161155450)

[20. Efficacy and Safety Evaluation Committee 37](#_Toc161155451)

[21. Research Organization 38](#_Toc161155452)

[21.1. Principal investigator 38](#_Toc161155453)

[21.2. Research Secretariat 38](#_Toc161155454)

[21.3. Co-Investigators 38](#_Toc161155455)

[21.4. Statistical Analyst 39](#_Toc161155456)

[21.5. Monitoring Officer 39](#_Toc161155457)

[21.6. Data Centers 39](#_Toc161155458)

[21.7. Efficacy and Safety Evaluation Committee 39](#_Toc161155459)

[22. References 40](#_Toc161155460)

## 1. Objectives

This study aims to evaluate the effectiveness of the glucose control enteral formula Glucerna®-REX (Abbott Japan) in suppressing hyperglycemia when used as a postoperative enteral nutrition formulation for esophageal cancer patients, in comparison to a standard enteral nutrition formula without glucose restriction (Meiji Main)

**Primary endpoint**:

The average value of Time in Range (TIR) measured by Continuous Glucose Monitoring (CGM) up to the second postoperative day.

**Secondary endpoints**:

1. Incidence rate of infectious complications during hospitalization

2. Incidence rate of infectious complications within 30 days postoperatively

3. Incidence rate of all complications during hospitalization

4. Incidence rate of adverse events

5. Average value of Time Above Range (TAR) during the entire measurement period

(postoperative days 1-8) measured by CGM

6. Daily TAR values measured by CGM up to postoperative day 8

7. Average value of Area Under the Curve (AUC) during the entire measurement period

(postoperative days 1-8) measured by CGM

8. Daily AUC values measured by CGM up to postoperative day 8

9. Average value of Time in Range (TIR) during the entire measurement period (postoperative days 1-8) measured by CGM

10. Daily TIR values measured by CGM up to postoperative day 8

11. Rate of change in nutritional indicators during hospitalization compared to the time of admission (serum albumin, prealbumin, and total protein levels)

12. Number of cases requiring blood glucose control intervention for hyperglycemia (≥ 300 mg/dL)

13. Number of cases requiring changes to enteral nutrition formulas from postoperative day 3 onwards

14. Number of cases requiring a reduction of more than 50% in enteral nutrition from the previous day after postoperative day 3

# 2. Background and rationale for the test plan

## 2.1. Background

Esophageal cancer remains a malignancy with a poor prognosis, having a 5-year survival rate of 40.6% [1]. Although advancements in medical technology have led to improved outcomes through multimodal therapies combining surgery with chemotherapy and radiotherapy, surgery still remains the only potential curative treatment. However, esophagectomy is known to be associated with a high frequency of postoperative complications among gastrointestinal surgeries. Among these, the incidence of infectious complications is particularly high, with serious conditions such as pneumonia and anastomotic leakage being not only prone to becoming severe but also impacting long-term prognosis. Therefore, reducing the risk of postoperative infectious complications is a critical challenge in the treatment of esophageal cancer [2].

In our hospital, between 2014 and 2018, postoperative infectious complications were observed in approximately 30% (127 patients) of 430 patients who underwent esophagectomy and reconstruction for esophageal cancer. One contributing factor is postoperative hyperglycemia, which impairs neutrophil chemotaxis and phagocytic function and, together with the increase in pro-inflammatory cytokines immediately after surgery, elevates the risk of infectious complications. It is well known that patients with diabetes are at particularly high risk of postoperative infectious complications [3].

In a retrospective study conducted prior to this trial, we examined the relationship between postoperative blood glucose levels and infectious complications in esophageal cancer patients. We reported that not only diabetic patients but also non-diabetic patients who experience postoperative hyperglycemia are at an increased risk of infectious complications. Moreover, surgical stress was found to contribute to postoperative hyperglycemia [4]. In our study, patients with an average blood glucose level of 200 mg/dL or higher on postoperative day 1 had a significantly higher incidence of infectious complications compared to those with an average blood glucose level below 200 mg/dL (hyperglycemic group: 39.3% vs. non-hyperglycemic group: 25.6%, P = 0.0068). Subgroup analysis showed that this trend was observed not only in diabetic patients (hyperglycemic group: 39.3% vs. non-hyperglycemic group: 25.6%, P = 0.0068) but also markedly in non-diabetic patients (hyperglycemic group: 42.9% vs. non-hyperglycemic group: 25.2%, P = 0.0022). Furthermore, multivariate analysis of subgroups revealed that an average blood glucose level of 200 mg/dL on postoperative days 1, 2, and 4 was an independent risk factor for infectious complications in non-diabetic patients [multivariate hazard ratio for hyperglycemic group compared to non-hyperglycemic group: postoperative day 1: 1.94, postoperative day 2: 3.68, postoperative day 4: 3.07].

One factor contributing to postoperative hyperglycemia is nutritional management. After esophageal cancer surgery, it often takes time for patients to resume adequate oral intake. As a result, enteral nutrition using semi-elemental formulas via a gastrointestinal fistula is frequently initiated immediately after surgery. Choosing an appropriate enteral formula according to the patient’s needs is crucial. For patients with diabetes, a glucose-restricted formula with a low-carbohydrate, high-fat composition is preferable to stabilize perioperative blood glucose fluctuations compared to standard enteral formulas. The enteral formula to be used in this study, Glucerna®-REX, is a glucose-restricted formula developed with a carbohydrate-to-total energy ratio restricted to 25% (compared to 60% in the standard enteral formula Meiji Main), with 50% of the energy derived from fats. Moreover, over 90% of the fats are composed of unsaturated fatty acids such as oleic acid, linoleic acid, and α-linolenic acid, which are less likely to have a negative impact on lipid metabolism compared to saturated fatty acids. Therefore, even in cases where blood glucose management is challenging with standard enteral formulas, using Glucerna®-REX is expected to provide better control over blood glucose levels. In clinical practice, it is already widely used as a postoperative enteral formula for diabetic patients.

However, the use of Glucerna®-REX for postoperative nutritional management in non-diabetic patients has not been common, and it remains unclear whether Glucerna®-REX can suppress early postoperative hyperglycemia in non-diabetic patients undergoing esophageal cancer surgery. Therefore, we have planned a single-center, open-label, randomized controlled trial to compare and evaluate the effectiveness of Glucerna®-REX in suppressing early postoperative hyperglycemia in non-diabetic patients with esophageal cancer, using the standard semi-elemental formula Meiji Main as a control. In this study, we will use the continuous glucose monitoring (CGM) system FreeStyle Libre Pro, which visualizes 24-hour blood glucose fluctuations, to measure postoperative blood glucose levels [5-7]. Using CGM allows for a detailed understanding of blood glucose variability, enabling accurate and comprehensive detection of potential hyperglycemia or hypoglycemia that may be missed with conventional intermittent measurements such as HbA1c or scheduled blood glucose monitoring. The aim of this study is not only to evaluate the effects of Glucerna®-REX but also to obtain data on blood glucose variability after administration of the standard enteral formula Meiji Main, which has not been reported to date.

## 2.2. Current status of our department

### 2.2.1 Enteral Nutrition

At our department, we have traditionally used the standard semi-elemental formula Meiji Main as a postoperative enteral nutrition formula for non-diabetic patients. The typical administration schedule is 400 mL on postoperative day 1, 800 mL on day 2, 1200 mL on day 3, and 1600 mL from day 4 onwards. On postoperative day 9, the formula is switched to the concentrated liquid formula HineX® E-gel at 1000 mL, and oral intake is initiated. After the initiation of oral intake, the enteral nutrition is gradually reduced according to the patient’s intake capacity. The timing of switching to HineX® E-gel and the initiation of oral intake, as well as the amount of Meiji Main administered, are adjusted as necessary based on the patient's condition..

### 2.2.2. Blood Glucose Monitoring and Management

During the Intensive Care Unit (ICU) stay, blood glucose levels are measured every 6 hours using arterial blood gas samples collected via the arterial line inserted into the radial artery. From postoperative day 4 onwards, while in the general ward, intermittent blood glucose measurements using finger-prick tests (typically four times a day: before each meal and before bedtime) are conducted if intervention is deemed necessary based on prior blood glucose trends.

Interventions for blood glucose levels (rescue treatment for hyperglycemia and hypoglycemia) are as follows:

| Blood glucose (mg/dL) | | Fast-acting insulin | 50% Dextrose |
| --- | --- | --- | --- |
| Hyperglycemia | 201 or more ~ 245 or less | 2 Units | - |
|  | 250 or more ~ 299 or less | 4 Units | - |
|  | 300 or more ~ 349 or less | 6 Units | - |
|  | 350 or more ~ 399 or less | 8 Units | - |
|  | 400 or more | 10 Units | - |
| hypoglycemia | 69 or less | - | 20mL |

Postoperative management of the current situation in our department

| Location  Days  Items | operating room | ICU | | | General Ward | | |
| --- | --- | --- | --- | --- | --- | --- | --- |
|  | Day0 | Day1 | Day2 | Day3 | Day4-8 | Day9 | Day 10 onwards |
| Surgery | ○ |  |  |  |  |  |  |
| Meiji Main |  | 400mL | 800mL | 1200mL | 1600mL |  |  |
| Hynex ® Eagle |  |  |  |  |  | 1000mL | Tapering as appropriate |
| Dietary intake |  |  |  |  |  | ○ | ○ |
| Blood glucose measurement (4 times/day) |  | ○ | ○ | ○ | ○ | ○ | ○ |
| Interventions for blood glucose levels |  | ○ | ○ | ○ | ○ | ○ | ○ |

## 2.3. Overview of the Study Intervention

### 2.3.1. Enteral Nutrition

The enteral formula used in this study, Glucerna®-REX, was developed as a glucose-restricted enteral formula and is already widely used in clinical practice. While standard enteral formulas are typically composed of 60% carbohydrates as their energy source, Glucerna®-REX is formulated as a low-carbohydrate enteral formula with a carbohydrate-to-total energy ratio limited to 25% (a 66% reduction compared to Meiji Main). Instead, it provides 50% of its energy from fats. Notably, more than 90% of these fats are unsaturated fatty acids such as oleic acid, linoleic acid, and α-linolenic acid, which are less likely to negatively affect lipid metabolism compared to saturated fatty acids. Therefore, using Glucerna®-REX is expected to have a stronger suppressive effect on blood glucose levels compared to standard enteral formulas.

### 2.3.2. Ingredients of enteral nutrition formula used in this study.

.

| Variable | MEIN (Meiji),  standard EN | Glucerna-REX (Abbott),  low-carbohydrate EN |
| --- | --- | --- |
| Capacity | 100mL | 100mL |
| Energy | 100Kcal | 100Kcal |
| Protein | 5g | 4.2g |
| **Lipids** | **2.8g** | **5.6g** |
| **Carbohydrates** | **15g** | **9.7g** |
| Glucidics | 13.2g | 8.8g |
| Dietary fiber | 1.8g | 0.9 |
| Fluids | 84.1g | 85g |
| Vitamin A | 150μg | 104μg |
| Vitamin B1 | 0.25mg | 0.12mg |
| Vitamin B2 | 0.3mg | 0.18mg |
| Vitamin B12 | 0.6μg | 0.3μg |
| Vitamin D | 0.75μg | 0.9μg |
| Folic acid | 50μg | 20μg |
| Vitamin C | 50mg | 11mg |
| Sodium | 80mg | 94mg |
| Potassium | 120mg | 100mg |
| Calcium | 100mg | 70mg |
| Osmotic pressure | 640mOsm/L | 560mOsm/L |

### 2.3.3. FreeStyleLibre Pro

The FreeStyle Libre Pro used in this study includes a blood glucose monitoring sensor and a Reader (Figure 1). Typically, the sensor is applied to the outer upper arm to ensure comfort and ease of movement. It should be placed avoiding areas with scars, moles, stretch marks, lumps, or sites used for insulin injections. In this study, once the patient returns to the ward immediately after surgery, a sub-investigator will attach the sensor to the outer upper arm of the patient and conduct continuous blood glucose monitoring from postoperative day 1 to day 8. On postoperative day 9, data will be extracted using the dedicated Reader through a scanning procedure, after which the sensor will be disposed of.

It should be noted that during routine postoperative X-ray examinations, it is not necessary to remove the sensor.

**Figure 1: Continuous blood glucose meter (CGM) used in this study: FreeStyle Libre Pro (Abbott Japan)**（<https://www.myfreestyle.jp/hcp/>）


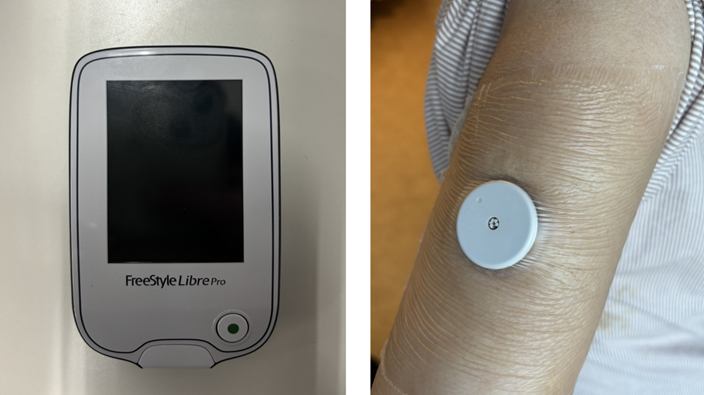


## 2.4. Study Design

The study will be conducted as a single-center, open-label, randomized, parallel-group study.

###
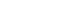
2.4.1. Blood glucose measurement using CGM

To verify the effects of the glucose-restricted enteral formula Glucerna®-REX, the following parameters obtained from CGM will be evaluated as indicators of its ability to suppress blood glucose elevation.

Primary outcome：

**TIR (Time in Range)**: Defined as the percentage of time during 24-hour continuous blood glucose monitoring in which blood glucose levels are within the target range of 70-180 mg/dL (the time period when the curve is within the red box in Figure 2 (hours)/24h).

Secondary outcomes：

**Time Above Range (TAR)**: Defined as the percentage of time during 24-hour continuous blood glucose monitoring in which blood glucose levels are ≥180 mg/dL, representing the hyperglycemic range (the sum of time periods labeled as ① in Figure 2 (hours)/24h) [7].

**Area Under the Curve (AUC)**: Defined as the area under the curve for blood glucose levels ≥180 mg/dL during 24-hour continuous blood glucose monitoring, representing the extent of hyperglycemia (the sum of the shaded areas labeled as ② in Figure 2) [9].

TIR (Time in Range) was introduced as a new glycemic control indicator in the "International Consensus on Glycemic Control Targets" presented at the American Diabetes Association (ADA) Scientific Sessions in June 2019 [8]. This indicator has been shown to correlate with conventional glycemic control markers, such as HbA1c, with a 10% increase in TIR being associated with an approximate 0.5% decrease in HbA1c (Figure 2) [9]. Additionally, TIR has the advantage of detecting hidden hyperglycemia and hypoglycemia that cannot be captured through intermittent measurements, making it a precise and comprehensive indicator. Given these attributes, TIR has been deemed an appropriate primary endpoint for this study.

**Figure 2: Blood glucose data using a continuous glucose meter (CGM)**

（Battelino T　et al. Diabetes Care. 2019［7］）


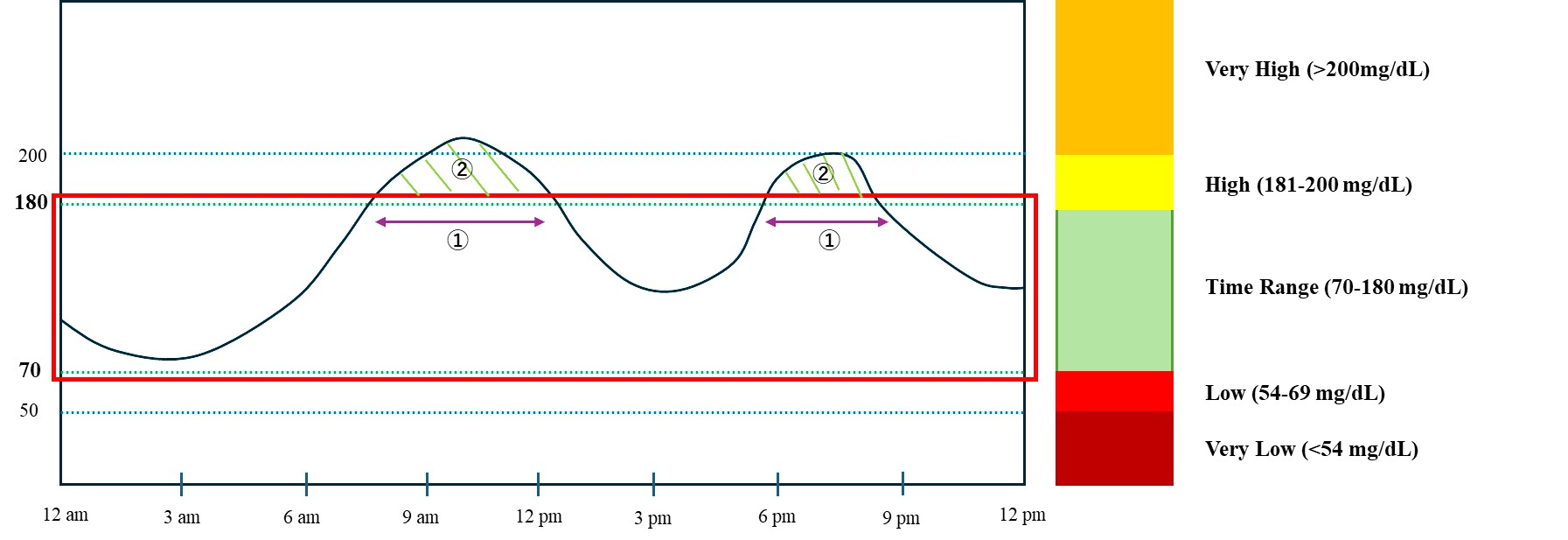


**Figure 3: Relationship between TIR values and corresponding HbA1c percentages**

(Koide et al. "How to make use of blood glucose data you want to read now (Nanzando)" 2020)


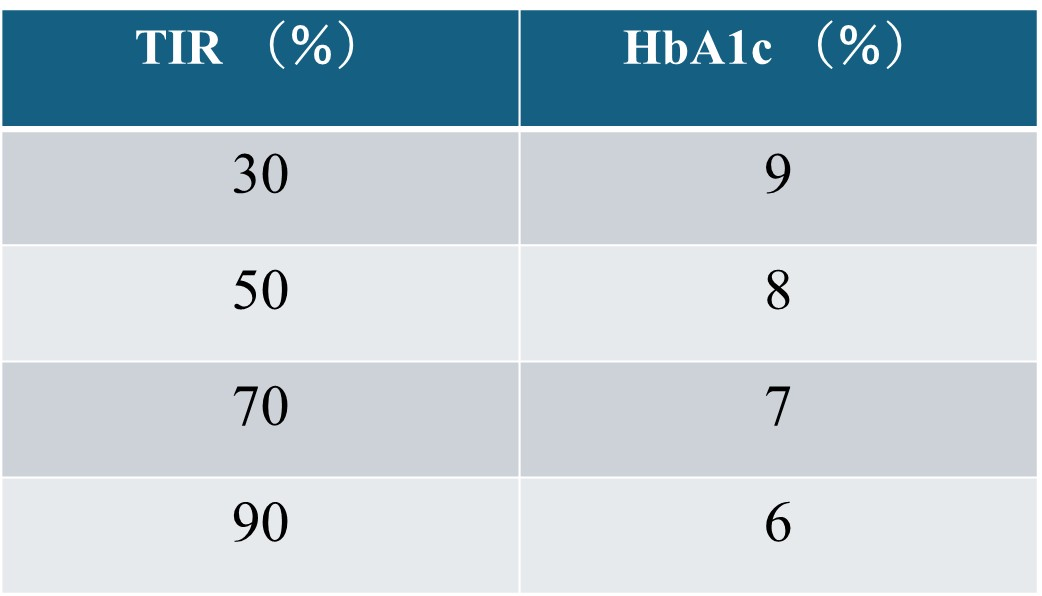


### 2.4.2. Clinical Hypothesis

The clinical hypothesis of this study is that "Glucerna®-REX suppresses postoperative blood glucose elevation in non-diabetic patients undergoing esophageal cancer surgery." The target sample size for this study is set at 50 patients. Due to difficulties in determining the sample size based on a statistical hypothesis, the number of cases was set according to the number of patients that can be enrolled during the study period. Assuming a dropout rate, with 48 patients included, and a standard deviation of 8.5% for the intergroup difference in TIR, it is expected that the mean intergroup difference in TIR can be estimated with a confidence interval width of approximately ±5%.

### 2.4.3. Prospects for Patient Enrollment

Currently, our facility performs approximately 110 cases of esophagectomy and reconstruction surgery for esophageal cancer annually. Taking into account the exclusion criteria, we expect to complete patient enrollment within 1.5 years after the jRCT disclosure

Planned number of registrations: 50 (25 people/group)

Registration period: 1.5 years after the jRCT disclosure

Follow-up period: 0.5 years after the end of enrollment

Analysis period: 1.0 years after completion of enrollment

Total study period: 2.5 years

## 2.5. Expected benefits and disadvantages associated with participation

### 2.5.1. Expected Benefits

Since this is a research study, no direct benefits for participants are anticipated.

### 2.5.2. Expected Burden and Disadvantages

The study will use Glucerna®-REX, Meiji Main, and FreeStyle Libre Pro, which are all widely used in routine clinical practice, as outlined in Section 2.1. Glucerna®-REX and Meiji Main will be provided as dietary components, and both the sensor and Reader for the FreeStyle Libre Pro will be purchased using the esophageal surgery research fund. Therefore, participating in this study will not impose any additional physical burden or economic disadvantage on the patients.

According to the study protocol, the frequency and parameters of blood tests will not exceed those of standard postoperative management. However, there is a slight risk of skin irritation or inflammation around the application site due to the adhesive used when attaching the FreeStyle Libre Pro sensor.

## 2.6. Significance of the Study

The significance of this study is to clarify whether nutritional management using the glucose-restricted enteral formula Glucerna®-REX has a stronger suppressive effect on postoperative blood glucose elevation in non-diabetic patients undergoing esophageal cancer surgery compared to the standard enteral formula without glucose restriction (Meiji Main). The findings from this study will be used to generate clinical hypotheses for future confirmatory trials.

## 2.7. Ancillary Research

No incidental studies will be conducted in this study.

# 3. Criteria and definitions used in this study

## 3.1. Infectious Complications

Infectious complications are evaluated in the "NCI-Common Terminology Criteria for Adverse Events v5.0 (CTCAE v5.0 Japanese)" Japanese (hereinafter referred to as CTCAE) v5.0-JCOG). When grading adverse events, the one that is closest to the definition of Grade 0~4 is judged accordingly.

## 3.2 Diagnostic Criteria for Esophageal Cancer

Matters related to the treatment of esophageal cancer will be conducted in accordance with the **Esophageal Cancer Treatment Guidelines (2022 Edition)**

# 4. Patient Eligibility Criteria

Patients who meet all of the inclusion criteria and do not meet any of the exclusion criteria will be included.

- 1. **Inclusion criteria**

1. Diagnosed histologically with esophageal cancer. Histological type is not specified..

②　 Do not have a history of diabetes mellitus (HbA1c less than 6.5 at first visit).

1. The scheduled surgery is subtotal esophagectomy with gastric tube reconstruction.
2. Be at least 20 years of age at the time of registration.

⑤　 Sufficient explanation of the study has been provided, and informed consent for

participation has been obtained.

⑥　 Organ function is preserved, and the patient is tolerant to general anesthesia.

- 1. **Exclusion Criteria**

1. Patients with distant metastases.
2. Patients who have undergone palliative resection.
3. Patients who have undergone second stage reconstruction.
4. Patients who underwent a total pharyngeal laryngeal esophagectomy.
5. Patients who required combined thoracic ductectomy.
6. Patients who have undergone preoperative chemoradiotherapy.
7. Patients who did not receive steroid prophylaxis for unavoidable reasons.
8. Patients with implanted medical devices such as pacemakers.

**Rationale for Exclusion Criterion ⑦:**

**Patients Not Receiving Prophylactic Steroid Administration**

Typically, steroids (Solumedrol 250 mg) are administered preoperatively to prevent postoperative systemic inflammatory response syndrome (SIRS). However, the presence or absence of steroid administration may potentially influence postoperative blood glucose fluctuations. Therefore, patients who have not received prophylactic steroid administration are excluded to minimize variability in postoperative blood glucose levels.

# 5. Registration and Allocation

## 5.1. Registration Procedure

After confirming that the patient meets all inclusion criteria and does not meet any exclusion criteria, informed consent will be obtained. The required information will then be entered and registered in the Electronic Data Capture (EDC) system managed by the Cancer Institute Hospital.

EDC system: Viedoc

URL：https://v4jp.viedoc.net/Login

Contact information for patient registration and inquiries regarding patient

selection criteria]

**Yu Imamura**

Department of Gastroenterological Surgery, Cancer Institute Hospital, Japanese Foundation for Cancer Research

〒135-8550

3-8-31 Ariake, Koto-ku, Tokyo

TEL：03-3520-0111

FAX：03-3570-0343

Email:[yu.imamura@jfcr.or.jp](mailto:yu.imamura@jfcr.or.jp)

**Masayoshi Terayama**

Department of Gastroenterological Surgery, Cancer Institute Hospital, Japanese Foundation for Cancer Research

〒135-8550

3-8-31 Ariake, Koto-ku, Tokyo

TEL：03-3520-0111

FAX：03-3570-0343

E-mail：masayoshi.terayama@jfcr.or.jp

[Inquiries that do not have clinical judgment such as EDC operation]

**Data Center: Yoshiko Matsui**

Advanced Cancer Treatment Development Center, Planning and Strategy Department, Cancer Institute Hospital, Japanese Foundation for Cancer Research

〒135-8550 3-8-31 Ariake, Koto-ku, Tokyo

TEL：03-3520-0111 (内線7512)　FAX:：03-3570-0701

E-mail：yoshiko.matsui@jfcr.or.jp

### 5.1.1. Notes on registration

Registration after the initiation of protocol treatment is strictly not permitted under any circumstances. If the entered data are incomplete, registration will not be accepted until all required information is provided. Registration is considered complete when a registration number is issued. Except in cases where consent is withdrawn, including refusal for research data usage, once a patient is registered, the registration cannot be cancelled or deleted from the database. In the event of duplicate registration, the initial registration information (registration number and assigned group) will be used in all cases. If erroneous or duplicate registrations are discovered, promptly notify the research secretariat.

## 5.2. Randomization and Randomization Factors

After registering a case in the EDC system, the patient will be randomly assigned to one of the groups by the EDC system. The allocation factors are surgical time (≥ 560 min or < 560 min) and HbA1c value (HbA1c < 6.0 or 6.1-6.4), which are factors of particular concern that may impact the primary outcome. The minimization method will be used to ensure that significant imbalances do not occur. Details of the allocation factors will remain blinded to the patients.

# 6. Treatment plan and Criteria for Treatment Modification

## 6.1. Protocol Treatment

### 6.1.1. Enteral Nutrition Used:

After esophagectomy, the control group will receive Meiji Main as the enteral nutrition formula, while the intervention group will receive Glucerna®-REX.

### 6.1.2. Dosing Schedule and Dosage

Starting from postoperative day 1, the control group will receive the standard enteral formula Meiji Main, while the intervention group will receive the glucose-restricted enteral formula Glucerna®-REX. Both formulas will be continuously administered for 24 hours at the following doses: 400 mL on postoperative day 1, 800 mL on day 2, 1200 mL on day 3, and 1600 mL from day 4 onwards. From postoperative days 4 to 8, the administration will continue at 1600 mL. On postoperative day 9, both groups will transition to standard postoperative management, switching to the concentrated liquid formula HineX® E-gel at 1000 mL, and initiate oral intake.

### 6.1.3. Blood Glucose Management

In this study, after the patient returns to the ward immediately post-surgery, the sub-investigator will attach the FreeStyle Libre Pro to the outer upper arm of the patient and begin continuous glucose monitoring (CGM). The sub-investigator will remove the FreeStyle Libre Pro from the patient on postoperative day 9. In addition, from the time the patient returns to the ward, blood glucose will be routinely measured 4 times a day using either blood gas analysis or the Dexter system.

Rescue treatments for hyperglycemia and hypoglycemia will be managed based on the blood glucose values obtained from the routine measurements via blood gas analysis or the Dexter system. Blood glucose information obtained from the FreeStyle Libre Pro will not be used as a reference for determining whether to perform rescue treatments for hyperglycemia or hypoglycemia.

Rescue treatments for hyperglycemia and hypoglycemia will be conducted as described below. However, rescue treatment for blood glucose levels between 201-299 mg/dL will not be permitted until the second postoperative day.

Criteria for rescue treatment for postoperative hyperglycemia and hypoglycemia

| Blood glucose (mg/dL) | | season | Fast-acting insulin | 50% Dextrose |
| --- | --- | --- | --- | --- |
| Hyperglycemia | 201 or more ~ 249 or less | Up to postoperative day 2 | None | - |
|  |  | From the 3rd postoperative day onwards | 2 Units | - |
|  | 250 or more ~ 299 or less | Up to postoperative day 2 | None | - |
|  |  | From the 3rd postoperative day onwards | 4 Units | - |
|  | 300 or more ~ 349 or less | Postoperative day 1 and beyond | 6 Units | - |
|  | 350 or more ~ 399 or less | Postoperative day 1 and beyond | 8 Units | - |
|  | 400 or more | Postoperative day 1 and beyond | 10 Units | - |
| hypoglycemia | 69 or less | Postoperative day 1 and beyond | - | 20mL |

<Rationale for rescue treatment for hyperglycemia and hypoglycemia>

Given that the study targets non-diabetic patients and the primary endpoint is the average TIR up to postoperative day 2, non-intervention with rapid-acting insulin will be permitted for blood glucose levels between 201 mg/dL and 299 mg/dL during this period. Conversely, if blood glucose levels fall below 69 mg/dL or exceed 300 mg/dL before postoperative day 2, non-intervention may pose a significant risk to patient safety. In such cases, intervention will be conducted using glucose administration or rapid-acting insulin, as appropriate.

## 6.2. Criteria for Protocol Treatment Completion, Discontinuation, or Modification

### 6.2.1. Definition of Protocol Treatment Completion

The initiation of protocol treatment is defined as the application of the FreeStyle Libre Pro. The protocol treatment is considered complete when the administration of Meiji Main, Glucerna®-REX, or any other nutritional formula is finished and the FreeStyle Libre Pro is removed.

If Meiji Main, Glucerna®-REX, or any other nutritional formula is administered up to postoperative day 8 and the FreeStyle Libre Pro remains attached until postoperative day 9, the protocol treatment is deemed complete

.

### 6.2.2. Criteria for Protocol Treatment Discontinuation

Protocol treatment will be discontinued in any of the following situations. The date of protocol treatment discontinuation will be defined as the date of death in the event of death or the date on which the attending physician determines that protocol treatment should be discontinued for other reasons.

When it becomes difficult to continue the study due to adverse events.

1. If the patient offers to discontinue protocol treatment.
2. Death during protocol treatment
3. When it is determined that discontinuation is necessary at the discretion of the principal investigator or co-investigator.
4. During the ICU period (within 4 days after surgery), the sensor is removed and the missing data is revealed.
5. By the second postoperative day, hyperglycemia (300 mg/dL or more) requiring insulin administration.
6. By the second postoperative day, the patient has a hypoglycemic condition (69 mg/dL or less) that requires sugar administration.
7. Skin troubles attributed to the FreeStyle Libre Pro sensor adhesive.
8. The need arises to remove the seal on the FreeStyle Libre Pro sensor for an MRI examination.
9. In addition, if serious deviations from the protocol are discovered, such as protocol violations, post-registration surgical procedures, or changes in treatment policies.

### 6.2.3. Change Criteria for Protocol Treatment

Adverse events related to the enteral formula (see Section 7.1.1) may necessitate changes to an alternative formula (selected from existing nutritional formulas deemed appropriate by the principal investigator or sub-investigator) or a reduction in the dosage of the enteral formula for both the control and intervention groups. If Grade 3 or higher symptoms (as defined by CTCAE ver. 5.0) are observed, both the control and intervention groups will either switch to an alternative nutritional formula, as determined appropriate by the principal investigator or sub-investigator or reduce the dosage of the protocol formula by 50% or more compared to the previous day, without changing the protocol formula itself.

## 6.3. Combined treatment and supportive treatment

### 6.3.1. Acceptable combination and supportive care for hyperglycemia and hypoglycemia

Rescue treatments for hyperglycemia and hypoglycemia (as outlined in Section 6.1.3 Blood Glucose Management) are the only interventions permitted.

### 6.3.2. Other Acceptable Concomitant and Supportive Cares

The administration of medications used in routine clinical practice, including probiotics, antidiarrheals, and intravenous fluids, is permitted.

### 6.3.2. Unacceptable Concomitant and Supportive Therapy

There are no prohibited concomitant therapies or supportive treatments..

# 7. Anticipated Adverse Events

## 7.1. Expected Adverse Events and Defects

### 7.1.1. Adverse Events Associated with Enteral Nutrition (Meiji Main and Glucerna-REX ®)

1. Aspiration
2. diarrhea
3. constipation
4. Bloating
5. stomach ache
6. nausea
7. vomiting
8. Small bowel obstruction
9. Hyperglycemia
10. hypoglycemia

### 7.1.2. Adverse Events Associated with Esophagectomy

1. Postoperative pneumonia
2. Recurrent laryngeal nerve palsy
3. Anastomotic Leakage
4. Chylothorax
5. bleeding
6. Anastomotic stenosis
7. Delayed gastric excretion
8. Pancreatic fistula
9. Wound infection
10. Intra-abdominal abscess
11. Pleural effusion

### 7.1.3. Adverse Events Associated with the Use of FreeStyle Libre Pro

Serious adverse events at the site of sensor fitting

1. Local infections

Adverse events at the site of sensor attachment

1. bleeding
2. erythema
3. edema
4. rash
5. itch
6. Purpura
7. hardening
8. infection
9. pain
10. inflammation

### 7.1.4. Malfunctions Associated with the Use of FreeStyle Libre Pro

1. Malfunction of the software, errors when reading data or displaying information, etc. (Reader)
2. The power does not turn on/electrical system troubles (Reader)
3. Poor adhesion (sensor)

## 7.2. Evaluation of Adverse Events / Adverse Reactions

Adverse events associated with enteral nutrition will be evaluated using CTCAE ver. 5.0, and adverse events associated with esophagectomy will be evaluated using the Clavien Dindo classification.

## 7.3. Adverse Event Observation Period

Observation of adverse events will be performed for 30 days after the end of administration of Meiji Main and Glucerna-REX ®.

## 7.4. Causality Assessment

The causality between the observed adverse events and the administration of enteral nutrition products as well as the use of FreeStyle Libre Pro is determined as “related” when there is a reasonable temporal relationship between the occurrence of the adverse events and the administration of enteral nutrition products and the use of FreeStyle Libre Pro, and when the cause cannot be explained by factors other than the administration of enteral nutrition products and the use of FreeStyle Libre Pro.

Table 7.2.3 Criteria for Determining the Causality between Adverse Events and Treatment

| causal relationship | decision | Concept of judgment |
| --- | --- | --- |
| with | definite | Adverse events are apparent or severe due to protocol treatment and are judged to be unlikely to be due to exacerbation of the underlying disease or other factors |
|  | probable | Adverse events are unlikely to have arisen or become severe due to exacerbation of the underlying disease or other factors, and are likely to be due to protocol treatment. |
|  | possible | It is more plausible to assume that adverse events were caused or severed by protocol treatment, and are less likely to be due to exacerbation of the underlying disease or other factors. |
| without | unlikely | It was judged that it was more plausible to attribute adverse events to an exacerbation of the underlying disease or other factors than to assume that the adverse events were caused or severed by protocol treatment. |
|  | not related | Adverse events are clearly caused or severed by exacerbation of the underlying disease or other factors, and are judged to have little chance of protocol treatment |

# 8. Evaluation items

## 8.1. Endpoints prior to enrollment (within 28 days prior to the date of surgery)

1. Patient Background

Gender, date of birth, height, weight, blood pressure, pulse, anamnesis, complications, allergy history, medications used

1. Subjective symptoms, other findings, general condition PS (ECOG)
2. Peripheral blood count: leukocyte count, neutrophil count (ANC: rod nucleocytes + segmental nucleocytes), lymphocyte count, hemoglobin, platelets
3. Blood biochemistry: total protein, albumin, total cholesterol, total bilirubin, AST (GOT),

ALT (GPT), BUN, Creatinine, eGFR, LDH, Calcium, Sodium, Potassium, CRP, FBS (Fasting Blood Glucose), HbA1c, Prealbumin

1. Urinalysis (occasional urine): urine protein qualitative, urine glucose qualitative, urine specific gravity, urine pH, urine urobilinogen, urine bilirubin
2. X-ray, (contrast-enhanced) CT examination
3. Electrocardiography

## 8.2. Evaluation items on the day of surgery

1. Surgery Information
2. Allocation
3. FreeStyle Libre Pro attachement
4. 24-hour continuous glucose monitoring
5. Confirmation of Survival

## 8.3. Evaluation Items on Postoperative Day 1

1. Conformation of FreeStyle Libre Pro Continues
2. 24-hour continuous glucose monitoring
3. Blood glucose measurement 4 times a day with blood gas
4. Continuous administration of 400 mL of enteral nutrition (Meiji Main and Glucerna®-REX)
5. If necessary, the rescue of insulin for hyperglycemia and hypoglycemia
6. Presence or absence of postoperative infectious complications
7. Presence or absence of other complications
8. Adverse events
9. Confirmation of survival or death

## 8.4. Evaluation Items on Postoperative Days 2 to 3

1. Conformation of FreeStyle Libre Pro Continues
2. 24-hour continuous glucose monitoring
3. Blood glucose measurement 4 times a day with blood gas
4. Continuous administration of enteral nutrition (Meiji Main and Glucerna-REX ®) (postoperative day 2; 800 mL, postoperative day 3: 1200 mL)
5. If necessary, the rescue of insulin for hyperglycemia and hypoglycemia
6. Presence or absence of postoperative infectious complications
7. Presence or absence of other complications
8. Adverse events
9. Confirmation of survival or death

## 8.5. Evaluation Items on Postoperative Days 4 to 8

1. Conformation of FreeStyle Libre Pro Continues
2. 24-hour continuous glucose monitoring
3. Blood glucose measurement 4 times a day with dexter
4. Continuous administration of enteral nutrition (Meiji Main and Glucerna-REX ®) (postoperatively 4 days or later; 1600 mL)

Dosage may be changed or enteral nutrition may be changed depending on the patient's condition (e.g., adverse events) (see 6.2.3.)

1. If necessary, the rescue of insulin for hyperglycemia and hypoglycemia
2. Presence or absence of postoperative infectious complications
3. Presence or absence of other complications
4. Adverse events
5. Confirmation of survival or death

## 8.6. Evaluation Items on Postoperative Day 9

1. Remove the FreeStyle Libre Pro. (Postoperative 1~Collection of blood glucose data on day 8)
2. Enteral nutrition: Change of administration to Heinegel (change timing can be changed depending on the patient's condition)

However, if Meiji Main and Glucerna-REX ® are still administered after the 9th postoperative day, until the end of administration

## 8.7. Evaluation Items at Discharge: Acceptable discharge date±7 days

1. General Condition: PS
2. Peripheral blood count: leukocyte count, neutrophil count (ANC: rod nucleocytes + segmental nucleocytes), lymphocyte count, hemoglobin, platelets
3. Biochemical tests: total protein, total protein, albumin, total cholesterol, total bilirubin, AST (GOT), ALT (GPT), BUN, creatinine, eGFR, LDH, calcium, sodium, potassium, CRP, FBS (fasting blood glucose), HbA1c, prealbumin
4. Presence or absence of postoperative infectious complications
5. Presence or absence of other complications
6. Adverse events
7. Confirmation of survival or death

If discharge occurs later than 14 days after surgery, tests and evaluations should be conducted on postoperative day 14 as the discharge day.

## 8.8. Endpoints 30 days after the last dose of Meiji Main and Glucerna-REX ® (post-observation): acceptable + 14 days

1. Presence or absence of postoperative infectious complications
2. Presence or absence of other complications
3. Adverse events
4. Confirmation of survival or death

## 8.9. Study Calendar

|  | STUDY PERIOD | | | | | | | Close-out | |
| --- | --- | --- | --- | --- | --- | --- | --- | --- | --- |
|  | Enrolment | Allocation | Post-allocation | | | | |  |  |
|  |  |  | ICU | | | General ward | |  |  |
| TIMEPOINT | -28～-1 | 0 | 1 | 2 | 3 | 4～8 | 9 | At discharge | 30 days after the final dose |
| **ENROLMENT**: |  |  |  |  |  |  |  |  |  |
| Eligibility screen | X |  |  |  |  |  |  |  |  |
| Informed consent | X |  |  |  |  |  |  |  |  |
| Allocation |  | X |  |  |  |  |  |  |  |
| **INTERVENTIONS** |  |  |  |  |  |  |  |  |  |
| Meiji Main |  |  | X | X | X | X |  |  |  |
| Glucerna-REX ® |  |  | X | X | X | X |  |  |  |
| CGM |  | X | X | X | X | X | X |  |  |
| Intermittent blood glucose measurement |  |  | X | X | X | X |  |  |  |
| Dosage of enteral nutrition formula (mL) |  |  | 400 | 800 | 1200 | 1600 |  |  |  |
| **ASSESSMENTS**: |  |  |  |  |  |  |  |  |  |
| Infections cmplications |  |  | X | X | X | X | X | X | X |
| Other complications |  |  | X | X | X | X | X | X | X |
| Adverse effects |  |  | X | X | X | X | X | X | X |
| survival |  | X | X | X | X | X | X | X | X |

# 9. Reporting of Adverse Events

The reporting of adverse events shall be in accordance with the provisions of this chapter based on the "Clinical Trials Act" (Act No. 16 of 29), the "Enforcement Regulations of the Clinical Trials Act" (Ordinance No. 17 of the Ministry of Health, Labour and Welfare of Heisei 30), and related notices. "In the event of a serious adverse event ("illness" under the Clinical Trials Act), the co-investigator shall report it to the principal investigator/research office.

1. The severity criteria used for reporting adverse events are "7.2. Assessment of adverse events/adverse reactions" will be used.
2. Adverse events for which a causal relationship with this study cannot be ruled out are called diseases.
3. Diseases that occurred after the start date of protocol treatment and up to 30 days from the date of completion of protocol treatment are reported.
4. When reporting illness, etc., use the latest version of the form obtained from the Ministry of Health, Labour and Welfare website.

[**http://www.mhlw.go.jp/stf/seisakunitsuite/bunya/0000163417.html**](http://www.mhlw.go.jp/stf/seisakunitsuite/bunya/0000163417.html)

## 9.1. Adverse Events Subject to Reporting Requirements

Adverse events (including diseases) that meet any of the following criteria and for which causality with this study cannot be ruled out shall be subject to reporting.

Table 9.1　Reporting Obligation and Reporting Deadline


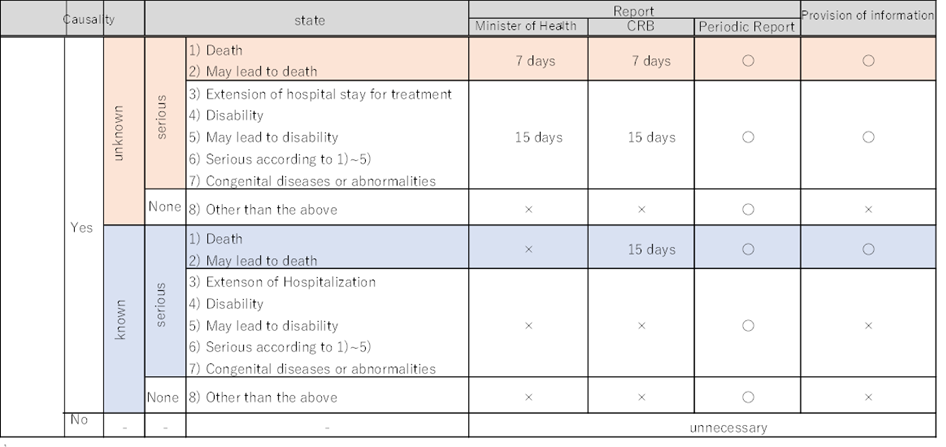


 **"Foreseeable: Unknown"** refers to those not listed in "7. Expected Adverse Events."

 **Death**: All deaths that occur after registration but before the initiation of protocol treatment, as well as all deaths that occur during protocol treatment or within 14 days from the date of treatment.

 **Life-threatening**: CTCAE Grade 4 events that occur during protocol treatment or within 14 days from the date of treatment.

 **Extension of hospitalization**: Events that occur during protocol treatment or within 14 days from the date of treatment.

※ "Hospitalization or extension of hospitalization" refers only to cases where hospitalization or an extension of hospitalization for 24 hours or more is medically necessary for the treatment of adverse events. The following cases are not subject to reporting:

- Hospitalization or extension of hospitalization for follow-up observation when the adverse event has resolved or improved.
- Hospitalization or extension of hospitalization to reduce the burden on the patient, such as in cases where the patient is coming from a remote area.
- Other hospitalizations or extensions of hospitalization that are not medically necessary.

.

## 9.2. Reporting Obligations and Procedures in the Event of Adverse Events or Diseases

### 9.2.1. Responsibilities of Personnel Engaged in This Study

Personnel engaged in this study must promptly report information regarding adverse events (including diseases) for which causality cannot be ruled out to the research office when they become aware of such events. However, reporting is not required if the adverse event is a progression (recurrence) of the underlying disease, a social hospitalization, or a hospitalization determined prior to participation in this study.

Even if the factors contributing to the occurrence of the adverse event are unclear, a preliminary report must be submitted based on the information available at that time. Subsequently, as soon as possible, the information that has been obtained up to that point should be documented and submitted as a follow-up report through the research office to the principal investigator. If it was initially determined that the causality could be ruled out but is later judged to correspond to a disease or other condition, the research office should be notified immediately at that point

.

### 9.2.2. Responsibilities of the Research Office

When the research office receives a report from personnel engaged in this study regarding the occurrence of an adverse event (including diseases) for which causality cannot be ruled out, it shall promptly provide a written report to the site administrator and the principal investigator.

### 9.2.3. Responsibilities of the Principal Investigator and the Research Secretariat

The principal investigator and the research office shall evaluate the urgency, significance, and impact of the reported disease or other conditions submitted to the research office, and take necessary measures such as temporarily suspending enrollment or issuing urgent notifications as needed.

### 9.2.4. Report to the Efficacy and Safety Evaluation Committee

If the principal investigator determines that an opinion from the Efficacy and Safety Evaluation Committee is required for a reportable adverse event, they shall promptly report the event to the committee and seek its opinion on the appropriateness of the principal investigator's perspective and the response to the adverse event.

### 9.2.5. Responsibilities of the Efficacy and Safety Evaluation Committee

The Efficacy and Safety Evaluation Committee will examine the details of the reported adverse events and make written recommendations to the principal investigator and the research office on measures to take, including whether or not to continue the study and whether or not to revise the protocol.

### 9.2.6. Report to the Accredited Clinical Research Review Board and the Minister of Health, Labour and Welfare

"Table 9.1) Refer to the "[Disease Report] Reporting Obligation and Reporting Deadline List", and if it is determined that the disease requires reporting, the report shall be made within the specified period.

**・Report to the Accredited Clinical Research Review Board**

Report to the Certified Clinical Research Board (CRB) using the "Pharmaceutical Disease Report (Unified Form 8)" and the "Detailed Description Form" stipulated in the Clinical Trials Act.

**・Report to the Minister of Health, Labour and Welfare**

Entering information into the "Disease Report" section of jRCT and sending an email to PMDA will serve as a report to the Minister of Health, Labour and Welfare

### 9.2.7. Periodic Reporting

The principal investigator shall report the occurrence of diseases to the CRB, the administrator of the medical institution, and the Minister of Health, Labour and Welfare in a periodic report conducted once a year on all diseases that have occurred in connection with clinical research.

# 10. Determination and endpoint definition

## 10.1. Evaluation items

**Primary endpoint**:

The average value of Time in Range (TIR) measured by Continuous Glucose

Monitoring (CGM) up to the second postoperative day.

**Secondary endpoints**:

1. Incidence rate of infectious complications during hospitalization

2. Incidence rate of infectious complications within 30 days postoperatively

3. Incidence rate of all complications during hospitalization

4. Incidence rate of adverse events

5. Average value of Time Above Range (TAR) during the entire measurement

period (postoperative days 1-8) measured by CGM

6. Daily TAR values measured by CGM up to postoperative day 8

7. Average value of Area Under the Curve (AUC) during the entire

measurement period

(postoperative days 1-8) measured by CGM

8. Daily AUC values measured by CGM up to postoperative day 8

9. Average value of Time in Range (TIR) during the entire measurement period

(postoperative days 1-8) measured by CGM

10. Daily TIR values measured by CGM up to postoperative day 8

11. Rate of change in nutritional indicators during hospitalization compared to

the time of admission (serum albumin, prealbumin, and total protein levels)

12. Number of cases requiring blood glucose control intervention for

hyperglycemia (≥ 300 mg/dL)

13. Number of cases requiring changes to enteral nutrition formulas from

postoperative day 3 onwards

14. Number of cases requiring a reduction of more than 50% in enteral

nutrition from the previous day after postoperative day 3y

# 11. Statistical Matters

Details of the statistical analysis are described in the statistical analysis plan to be prepared separately. For analyses that have been changed among the analyses planned by the protocol, the reason for the change shall be described in the statistical analysis plan.

## 11.1. Definition of Analysis Populations

The definition of the target population for this study is as follows. The handling of each case will be decided through consultation between the principal investigator, the research secretariat, the chief statistical analyst, and the data center before the data is fixed.

The efficacy analysis, including the main analysis, will be conducted in the Full Analysis Set (FAS), and the safety analysis will be performed in all treatment cases. The main analysis is a sensitivity analysis targeting the Per Protocol Set (PPS).

**・All Registered Cases**
All patients who have been registered are referred to as "All Registered Cases."

- **Full Analysis Set (FAS)**
   The group derived from the "All Registered Cases" after excluding patients with significant eligibility criteria violations, patients who violated exclusion criteria, patients who did not receive any protocol treatment, patients with no efficacy-related data, and patients who withdrew consent and refused to allow the use of any data. This group is defined as "FAS."
- **PPS (Per Protocol Set)**
   The group derived from the "FAS" after excluding patients who discontinued protocol treatment by the second postoperative day or had significant protocol violations is defined as "PPS."
- **All Treated Cases**
   Patients who received part or all of the protocol treatment are referred to as "All Treated Cases".

## 11.2. Handling of Missing Data

As a general rule, missing data will not be imputed. However, if necessary, analyses with imputed missing data will be conducted as sensitivity analyses. Details on the handling of missing data will be described in the statistical analysis plan. If the sensor detaches for any reason during the ICU stay, resulting in missing data, a sensitivity analysis will also be performed on cases where the data collected from the time the sensor was attached until the time it detached covers 70% or more (6.3 days or more) of the planned measurement days, based on the previously reported recommendations [8, 12].

## 11.3. Main Analysis

In this study, the primary analysis will focus on the average Time in Range (TIR) measured by CGM (Continuous Glucose Monitoring) up to the second postoperative day, which is the primary endpoint to be evaluated after patient enrollment is complete. Using analysis of covariance (ANCOVA), the adjusted mean difference in the primary endpoint between the two groups (intervention group - control group) will be calculated along with the 95% confidence interval and standard deviation. Additionally, as a reference, the p-value for the null hypothesis that the mean difference between the two groups is zero will be provided, along with the mean value (least-squares mean) and its 95% confidence interval and standard deviation for each group, including the results without adjusting for allocation factors. The average TIR up to the second postoperative day is defined as the proportion of time within the 48-hour period where blood glucose is within the therapeutic range of 70-180 mg/dL.

## 11.4. Sub-evaluation projects (efficacy).

For the following items, calculate the mean values and the corresponding 95% confidence intervals and standard deviations for each group and the intergroup difference (intervention group - control group). If necessary, calculate the p-values using Welch's t-test for comparisons between the two groups.

- Average TAR (Time Above Range) over the entire measurement period (Postoperative Day 1 to Day 8) measured by CGM
- Daily TAR up to Postoperative Day 8 measured by CGM
- Average AUC (Area Under the Curve) over the entire measurement period (Postoperative Day 1 to Day 8) measured by CGM
- Daily AUC up to Postoperative Day 8 measured by CGM
- Average TIR (Time in Range) over the entire measurement period (Postoperative Day 1 to Day 8) measured by CGM
- Daily TIR up to Postoperative Day 8 measured by CGM

## 11.5. Sub-secondary evaluation (safety)

For the following items, calculate the frequency, percentage, and corresponding 95% confidence interval for each group. The 95% confidence intervals should be calculated using Clopper & Pearson's exact confidence interval method. If necessary, perform a chi-squared test to compute the p-values for comparisons between the two groups.

For adverse events, calculate the number of cases, number of occurrences, proportion of cases, and their 95% confidence intervals using Clopper & Pearson's exact method. The analysis should also include a breakdown by grade and causal relationship. Additionally, create a comprehensive list of all adverse events.

- Incidence rate of infectious complications during the hospitalization period
- Incidence rate of infectious complications within 30 days postoperatively
- Incidence rate of all complications during the hospitalization period
- Incidence rate of adverse events
- Number of cases requiring glycemic control intervention for hyperglycemia (≥ 300 mg/dL)
- Number of cases in which enteral nutrition was modified after postoperative Day 3
- Number of cases requiring a reduction of more than 50% of enteral nutrition compared to the previous day after postoperative Day 3

For the following items, calculate the median and interquartile range (IQR) for each group at all measurement points. Also, calculate the median and IQR for changes from baseline values (pre-registration measurements). If necessary, also calculate the mean and standard deviation, as well as p-values using the Wilcoxon test.

- Nutritional indicators during the hospitalization period (serum albumin levels, prealbumin levels, and total protein levels)

)

## 11.6. Planned Registrations

1. **Planned Number of Cases**: 50 cases (25 cases in the control group, 25 cases in the intervention group).
2. **Rationale for the Target Sample Size**
   It is challenging to set the sample size based on a statistical hypothesis (because there are no published studies reporting the glucose-lowering effect of Glucerna®-REX). Assuming a dropout rate, if 48 cases are included, and the standard deviation of the intergroup difference in TIR is 8.5%, the mean intergroup difference in TIR can be estimated with a confidence interval width of approximately ±5%.

Currently, the Esophageal Surgery Department at the Cancer Institute Hospital of JFCR (Japanese Foundation for Cancer Research) performs approximately 110 esophagectomy and reconstruction surgeries annually for esophageal cancer. Considering exclusion criteria, it is expected that patient enrollment will be completed within 1.5 years after approval for implementation.

## 11.7. Interim Analysis

No interim analysis was performed in this study.

## 11.8. Intentional level and multiplicity

The significance level for all analyses in this trial is 5% two-tailed, and the confidence factor for the confidence interval is 95% two-tailed. Neither efficacy nor safety evaluation will be adjusted for multiplicity between endpoints or time points.

## 11.9. Final Analysis

After the end of the follow-up period, the data will be determined by the final survey, and then analysis will be performed on all endpoints.

# 12. Discontinuation of the entire study

## 12.1. Discontinuation criteria for the entire study

 If it is determined that completing the study is difficult due to reasons such as delays in patient enrollment or frequent protocol deviations.

 If it is determined that the risk-to-benefit ratio is unacceptable (including cases where new safety findings from this study or interim analysis results indicate such concerns).

 If an evaluation of related information obtained from sources such as publications or conference presentations outside of this study determines that there are safety concerns regarding this study, or if it is judged that there is no longer any significance in continuing the study

## 12.2. Procedures for Discontinuation of Clinical Research

1. If the Principal Investigator decides to terminate the clinical study based on the "12.1 Criteria for Study Termination," the investigator, along with the co-investigators, shall take appropriate measures for the study participants. If necessary, the opinions of the CRB (Certified Review Board) may be sought regarding the timing and method of concluding the study for the participants.

2. Within 10 days of terminating the clinical study, the Principal Investigator must notify the CRB listed in the study protocol using the "Termination Notification Form (Standard Form 11)" and create a report using [Ministerial Form No. 4] in the jRCT system to notify the Minister of Health, Labour and Welfare.

3. Even if a "Termination Notification Form (Standard Form 11)" is submitted, if any changes occur during the period until the clinical study is officially concluded that correspond to a change in the study's progress, an amendment notification to the study protocol must be submitted.

4. Even after submitting a "Termination Notification Form (Standard Form 11)," disease reports and periodic reports must continue to be submitted until the clinical study is officially concluded.

5. The official termination of the clinical study occurs when all measures for the study participants are completed and the study is concluded.

6. If the clinical study is terminated, and the "Termination Notification Form (Standard Form 11)" has been submitted and all measures for the study participants have been completed, a final report must be submitted within one year, in principle, from the later of either the termination date or the date when data collection for all evaluation items is completed

.

# 13. Ethical Matters

## 13.1. Patient Protection

All researchers involved in this study shall conduct the study in accordance with the following guidelines:

1. **"Ethical Guidelines for Life Sciences and Medical Research Involving Human Subjects"**
   (Issued on March 23, 2021, partially revised on March 27, 2023, by the Ministry of Education, Culture, Sports, Science and Technology, Ministry of Health, Labour and Welfare, and Ministry of Economy, Trade and Industry)
2. **“Declaration of Helsinki"**(Adopted in Fortaleza, Brazil, 2013)
3. **"Clinical Research Act"**　(Act No. 16 of 2017) and "Regulations for the Enforcement of the Clinical Research Act" (Ministry of Health, Labour and Welfare Ordinance No. 17 of 2018) as well as related notifications
4. Prior to the start of the study, the investigator shall submit an implementation plan* to the Minister of Health, Labour and Welfare with the approval of the CRB for the ^implementation^ of the study (registration of the implementation plan and publication of research information at the jRCT).In addition, permission to conduct the research must be obtained from the administrator of the medical institution.

^*^ Ministerial Ordinance Form 1 stipulated in Article 39, Paragraph 1 of the Ordinance for Enforcement of the Clinical Research Act

## 13.2. Informed Consent

### 13.2.1. Explanation to Patients

Prior to patient enrollment, the Principal Investigator or Sub-Investigator shall provide the patient with the informed consent document approved by the CRB and explain the following items in detail verbally.

Information to be Explained to the Patient:

1. That this study is a clinical trial
2. The study design and rationale (the significance, necessity, objectives, etc.)
3. Contents of the protocol treatment
4. Expected effects of the protocol treatment
5. Expected adverse events, complications, sequelae, and their management
6. Costs associated with the study

Explain that the costs of treatment and compensation therapy will be covered under the insurance system and that any compensation for health damage will be handled similarly to general clinical practice.

1. Expected benefits and potential risks

Explain the potential benefits the patient may receive by participating in the study and any possible disadvantages they may experience.

1. Refusal and withdrawal of consent

Explain that the patient is free to refuse participation in the study prior to giving consent, and that even after giving consent, they are free to withdraw it at any time without facing any unjust disadvantages in their medical care.

1. Protection of human rights

Maximum efforts will be made to protect the confidentiality of the patient’s name and personal information.

1. Conflict of interest related to the clinical study
2. Publication of research results

The results obtained from this clinical trial will be published in academic journals and presented at academic conferences. However, no personal information will be included in the published content.

1. Secondary use of data

Data may be used secondarily (e.g., in meta-analyses) without linking it to personal identification information, only if approved by the review committee.

1. Intellectual property rights

Any intellectual property rights resulting from this research will belong to the Cancer Institute of the Japanese Foundation for Cancer Research (JFCR).

1. Research organization
2. Freedom to ask questions

Provide contact information not only for the attending physician but also for the research director at the medical institution and the principal investigator (or research office) in writing so that the patient can freely ask questions regarding the study or treatment details.

### 13.2.2. consent

The Principal Investigator or Sub-Investigator, who is the attending physician, will explain the study to the patient, allowing sufficient time for consideration, and confirm that the patient fully understands the contents of the study before requesting participation. If the patient agrees to participate, the patient or a legal representative will sign the informed consent form using the supplementary consent form attached to the explanation and consent documents. The attending physician must verify that the informed consent document includes the name of the physician who provided the explanation, the name of the patient who received the explanation and consented, and the date of consent.

Two copies of the informed consent document will be prepared: one copy will be handed to the patient, and the other will be kept by the Principal Investigator. The original document will be stored in the patient’s medical record or in a designated storage location within the medical institution.

### 13.2.3. Withdrawal of consent

- After obtaining consent for study participation, if the patient expresses a desire to withdraw their consent for participation, the patient or their legal representative will sign a withdrawal form using the supplementary consent withdrawal document attached to the explanation and consent forms, thereby formalizing the withdrawal of consent. The attending physician must verify that the consent withdrawal document includes the patient’s name and the date of withdrawal. Two copies of the consent withdrawal document will be prepared: one copy will be given to the patient, and the other will be retained by the Principal Investigator. The original document will be stored in the patient’s medical record or in a designated storage location within the medical institution.
- **Withdrawal of consent** means the revocation of consent to participate in the study and is distinct from whether the protocol treatment will be continued (see point ① below). When withdrawal of consent is expressed, it must be clearly categorized as either point ② or point ③ below, and the research office must be promptly notified.
- If the research office confirms ② "partial consent withdrawal," it will stop any further follow-up requests according to the protocol. In the case of ③ "complete consent withdrawal," once complete withdrawal is confirmed, the patient's data will be deleted from the database.
- The procedures for stopping follow-up requests and deleting patient data will be specified separately in the standard operating procedures (SOPs), and the completion of these actions will be reported to both the Principal Investigator and the research office
  - 1. Patient refusal: Refusal to continue further protocol treatment (follow-up continues)
    2. Withdrawal of consent: Withdraw consent to participate in the trial and prevent all subsequent treatment and follow-up according to the protocol. It is possible to use the data on a trial basis before the withdrawal of consent.
    3. Withdrawal of All Consent: Withdraw your consent to participate in the study and make it impossible to use all data from the time of participation in the study, including information from the time of registration.

## 13.3. Personal Information

We recognize that privacy-related information, such as personal information and medical information, should be strictly protected and handled carefully under the principle of respecting the individuality of individuals, and we will take all possible management measures to protect privacy.

### 13.3.1. Policies, Laws, and Norms to Follow

In conducting research, in principle, the following laws and regulations shall be followed. If laws, regulations, norms, or policies other than the following apply, we will additionally comply with them.

1. Act on the Protection of Personal Information (Act No. 57 of 15 years last revised: Law No. 65 of September 9, 27)
2. Ethical Guidelines for Life Science and Medical Research Involving Human Subjects
3. Clinical Research Act, Enforcement Rules of the Clinical Research Act, and Notification of Closure
4. Declaration of Helsinki (translated by the Japan Medical Association)

### 13.3.2. Protection of Personal Information and Patient Identification

In order to protect personal information, the study will anonymize patients using a registration number issued at the time of patient registration. We do not use information that can identify a specific individual, such as initials or medical record IDs. The principal investigator will strictly store and manage the data to prevent leakage of the patient's personal information. In addition, when reporting or announcing the results of research, it will be anonymously processed and made public.

### 13.3.3. Purpose of use of personal information, items to be used, and method of use

- In this study, personal information will be used for the purpose of identifying individual patients and conducting investigations to obtain accurate results from the clinical research.
- The personal information used for patient identification and reference includes age, date of birth, and gender.
- Personal information of patients used in this study will be entered by the Principal Investigator or Sub-Investigators into various case report forms (CRFs) and, in principle, collected via Electronic Data Capture (EDC).
- Reports on notifiable diseases, etc., will be collected by submitting them to the research office via email, postal mail, or by hand. However, for urgent communication of patient information, phone calls may be used. In such cases, the content of the phone communication must be recorded in the medical records.
- When inquiries or reports are exchanged between the research office and medical institution researchers via email or other means, only registration numbers with a higher degree of anonymity should be used; medical record numbers or initials must not be used

## 13.4. Source material

The original materials related to clinical research used in this study refer to all records used for diagnosis and treatment, including medical records (including worksheets, etc.), examination records, images used for diagnosis, pathology certificates, images used for effect judgment, and consent documents of registered patients in this study (if the regulations of the medical institution allow digitized paper documents to be used as originals, digitized documents are treated as original documents). They will be made available for direct inspection during monitoring, audits (conducted as appropriate), and CRB and regulatory investigations.

## 13.5. Storage of samples, information, etc.

Samples and information of registered patients related to this study should be kept in accordance with Article 53 of the "Enforcement Regulations of the Clinical Trials Act (Ministry of Health, Labour and Welfare Ordinance No. 30 of 17)". The retention period for records related to the study and the retention period for the original materials at the participating sites shall be 5 years from the date of completion of the clinical study. It is recommended to store it for as long as possible after the expiration date.

## 13.6. Indemnification

If a health injury occurs to a study participant as a result of participating in this study, the Principal Investigator or Sub-Investigator will provide appropriate treatment and take any other necessary measures. Such treatment and related procedures will be conducted under the health insurance system, and the study participant will be responsible for paying the out-of-pocket portion of the medical expenses. In addition, to prepare for any unforeseen health injuries resulting from this study, clinical research insurance (covering compensation, indemnities, and medical expenses or allowances) will be obtained.

## 13.7. Intellectual Property

1. The intellectual property rights of the results and data obtained from this study belong to the Cancer Institute Hospital.

## 13.8. Protocol Compliance

1. Researchers participating in this study will comply with this protocol as long as it does not compromise patient safety and human rights.

## 13.9. Application to CRB and Notification of Implementation Plan

- In order to conduct this study, it is necessary to obtain the approval of the CRB and the administrator of the site to conduct the study using this protocol and the written explanation to patients.
- At the jRCT, we will submit an implementation plan to the Minister of Health, Labour and Welfare and publish research information such as the outline of this study, progress, and main results.

**【Clinical Research Review Board (CRB)】**

Credential ID CRB3220003
Address: 3-8-31 Ariake, Koto-ku, Tokyo

Phone: 03 3520 0703
Email Address: ganken_crb jfcr.or.jp

# 14. Periodic Reporting

In accordance with the notification titled *"Implementation of the Regulations for the Enforcement of the Clinical Research Act"* (February 28, 2018, Isei Keihatsu No. 0228-1 by the Director of the Economic Affairs Division, Health Policy Bureau, Ministry of Health, Labour and Welfare, and Isei Kenhatsu No. 0228-1 by the Director of the Research and Development Division), the Principal Investigator must report to the administrator of their affiliated medical institution annually, starting from the initial date of public disclosure in jRCT, and within 2 months after the expiration of that period, and then seek the opinion of the Certified Review Board (CRB) regarding the appropriateness of continuing the clinical research.

The periodic report should be prepared using [Periodic Report (Standard Form 5)] and should include a concise description of the following items:

1. Number of participants enrolled in this study
2. Occurrence status of diseases or events related to this study and their subsequent progress
3. Occurrences of non-compliance with the Clinical Research Act Regulations or the study protocol and the subsequent responses
4. Evaluation of the safety and scientific validity of the study
5. Matters related to involvement with pharmaceutical or medical device manufacturers as stipulated by the Conflict of Interest Management Standards

The Principal Investigator must submit a report (using [Periodic Report Form (Annex Form 3)]) to the Minister of Health, Labour and Welfare via jRCT and disclose it publicly within one month from the date of receiving the CRB's decision regarding the appropriateness of study continuation.

# 15. Management of Conflicts of Interest (COI) of Persons Involved in Clinical Research

1. This study is self-funded by the Department of Esophageal Surgery, Ariake Cancer Institute Hospital, and will not be funded by a specific company, and there will be no conflict of interest.
2. The COI related to this study will be managed as follows in accordance with the "Guidance on Managing Conflicts of Interest in the Clinical Trials Act" (November 30, 2011, 1130 No. 17, Notice of the Director of the Research and Development Promotion Division, Medical Policy Bureau, Ministry of Health, Labour and Welfare^)^ (hereinafter referred to as the "Guidance").
   ^*^ [http://www.mhlw.go.jp/stf/seisakunitsuite/bunya/0000163417.html The](http://www.mhlw.go.jp/stf/seisakunitsuite/bunya/0000163417.html)The related companies for this study will not be involved in the planning of the study, data acquisition, data analysis, discussion of the analysis results, presentation, or manuscript preparation. There are no conflicts of interest with companies or other entities related to this study.

# 16. Changes to the contents of documents approved by the CRB

## 16.1 Changes to the Implementation Plan

If there is any change to the implementation plan (Ministerial Form No. 1), it must be submitted as a "Notification" to the Minister of Health, Labour and Welfare via jRCT after obtaining approval from the CRB, and the changes must be made public.

## 16.2 Changes to the Protocol

In this study, protocol changes are categorized into two types: *Revisions* and *Amendments*. Additionally, supplementary explanations that do not correspond to protocol changes are distinguished as *Memorandums*. The categorization of revisions and amendments is determined based on the following principles:

1. **Amendment**
   - A partial change in the protocol that may increase the risk to patients participating in the study or have a substantial impact on the primary endpoint of the study.
   - When a change is deemed an "Amendment," patient enrollment will be temporarily suspended if it is ongoing at that time.
2. **Revision**
   - A change in the protocol that does not increase the potential risk to patients participating in the study and does not have a substantial impact on the primary endpoint.
   - In principle, patient enrollment is not temporarily suspended during a "Revision."
3. **Memorandum**
   - A supplementary explanation distributed by the Principal Investigator or the research office to study-related personnel, intended to reduce variability in the interpretation of the protocol text or to provide a reminder, without changing the protocol content. The format is not restricted.
   - However, if the content corresponds to a protocol change but is necessary to immediately share information among study-related researchers to reduce the risk to enrolled patients, a memorandum can be issued as a preliminary measure before submitting a revision request

.

## 16.3 Changes to the Consent Brief and Explanation and Re-Consent of the Patient

1. Any changes will be reported to the CRB for approval.
2. If there are any changes to the explanatory documents and consent documents, the patient shall be informed of the changes and the patient shall obtain written consent to continue the participation in the clinical study.

## 16.4 Other Approved Documents

1. Any changes will be reported to the CRB for approval.

# 17. Management of nonconformances

## 17.1. Non-Conformances

Non-conformity in the Clinical Trials Act refers to "a state in which clinical research does not conform to the Enforcement Regulations of the Clinical Trials Act or the research plan", and "Enforcement of the Enforcement Regulations of the Clinical Trials Act (February 28, 30)" "Non-compliance with rules, research plans, procedures, etc., falsification, fabrication, etc." of research data is cited as an example.

## 17.2. Material Non-Conformities

Material non-conformities are those that affect the human rights and safety of clinical research subjects and the reliability of research progress and results. Examples of "serious non-conformities" are shown below. If there is a possibility that these serious non-conformities may constitute, the Research Secretariat will report the situation to the CRB as soon as it becomes aware of the situation.

**1) Material non-conformity with respect to eligibility**

Violation Registration

・ Knowingly (falsely) registering while knowing that it does not meet the eligibility criteria

・Patient registration without the necessary informed consent and protocol treatment

・ The original documents for determining eligibility cannot be confirmed (including loss of consent form)

**2) Protocol violations**

Violations that affect an increased risk for enrolled patients or that affect the reliability of

trial results

・Serious violation of the eligibility standards and exclusion standards

・Violation of discontinuation criteria that threatens patient safety

・Serious violations of prohibited concomitant drugs and non-compliance with prohibited

combination therapies

・Intentional or systematic non-compliance with protocol regulations, etc.

**3) Other material non-conformities**

The study was conducted prior to CRB approval or approval by the administrator of the

conducting medical institution.

・ The trial was continued without providing information that may affect the intention to

continue the study.

・Findings that are judged to be research misconduct (fabrication of data, falsification of

data, etc.)

・Leakage of personal information or violation of human rights that has a significant

impact on registered patients

Serious non-conformity does not include those who did not comply with the research protocol in order to avoid immediate danger to the research subject or for other medically unavoidable reasons.

## 17.3. Not suitable for reporting

- - 1. Personnel involved in this study must promptly report to the Principal Investigator at their affiliated medical institution when they become aware of any non-compliance.
    2. The Principal Investigator must promptly report the non-compliance to the administrator of their affiliated medical institution and notify the Principal Investigator.
    3. If it is determined that the non-compliance affects the rights and safety of the study participants or the reliability of the study's progress or results, it must be treated as a serious non-compliance. In such cases, the Principal Investigator must promptly submit a "Serious Non-Compliance Report (Standard Form 7)" to the CRB and seek their opinion.
    4. Measures to prevent recurrence of the serious non-compliance must be implemented, communicated to the Sub-Investigators and other personnel involved in the clinical study at the affiliated medical institution, and efforts must be made to ensure thorough prevention of recurrence.

# 18. Monitoring and Auditing

## 18.1. Periodic monitoring

The study will provide central monitoring to ensure that the study is conducted safely and in accordance with protocols, and that data is being collected accurately. Central monitoring shall be carried out in accordance with the monitoring procedure.

## 18.2. Audits

No audits are conducted.

# 19. Termination of the entire study

## 19.1. Termination of Study

The jRCT will report to the Minister of Health, Labour and Welfare ([Summary of the Summary Report (Notification Appendix Form 3)]) and the date of publication shall be the date on which the clinical study was completed. "Enforcement Notice of the Enforcement Regulations of the Clinical Research Act (Ministry of Health, Labour and Welfare Ordinance No. 17 of February 28, 30) Article 24, Paragraph 1 of the Regulations (24)"

## 19.2. Procedure for Termination of Research

1. When the period for collecting data related to all endpoints described in the research plan has expired, the investigator shall prepare a summary report and a summary of the summary report within one year, starting from that date in principle.

The summary report should include at least the following items: "Enforcement Notice of the Enforcement Regulations of the Clinical Trials Act (Ministry of Health, Labour and Welfare Ordinance No. 17 of February 28, 30) Article 24, Paragraph 2 of the Regulations (25)"

1. Background information of clinical research subjects (age, gender, etc.)
2. Information on the progress of clinical research according to the design (changes in the number of participants, etc.)
3. Summary of the occurrence of diseases
4. Data analysis and results of primary and secondary endpoints
5. After preparing the summary report and the summary of the summary report, submit it to the CRB without delay and listen to the opinions of the committee. "Article 24, Paragraph 4 of the Enforcement Regulations of the Clinical Research Law (Ministry of Health, Labour and Welfare Ordinance No. 17, February 28, 30)"

Documents to be submitted to the CRB

1. Notice of Termination (Uniform Form 12)
2. Summary Report
3. Notice of Termination (Notice Appendix Form 3) (Summary of the Summary Report)
4. Within one month from the date on which the CRB expresses its opinion, the Principal Investigator shall report the summary report and the summary of the summary report to the administrator, and the jRCT shall report to the Minister of Health, Labour and Welfare ([Summary of the Summary Report (Appendix Form 3)]) and make it public. "Article 24, Paragraph 5 of the Enforcement Regulations of the Clinical Trials Act (Ministry of Health, Labour and Welfare Ordinance No. 17, February 28, 30)"

The following documents are attached to the publication of the jRCT.

1. Final version of the research proposal
2. Final version of the explanatory consent document
3. If a statistical analysis plan is prepared, the statistical analysis plan

With regard to the publication of the summary of the summary report, if the results of the research are to be published in a paper, etc., it is acceptable to report to the CRB that the paper is being submitted and after the publication of the paper, etc. Even in this case, the notification and report at the jRCT must be made within the deadline, and the timing of publication should be notified at the time of notification and reporting. However, when a research paper is published, it should immediately be a summary of the summary report, and the items submitted that were not filled in at the time of notification ("date of publication of the first publication" and "URL of the results and publication") should be recorded in the jRCT and made public. Enforcement Notice of the Enforcement Regulations of the Clinical Trials Act (Ministry of Health, Labour and Welfare Ordinance No. 17 of February 28, 30) Article 24, Paragraph 4 of the Regulations (27)"

## 19.3. Handling of Research Results

### 19.3.1. Publication of research results

The primary publication of the study results (the first paper to report on the primary endpoint results) will be submitted to an English-language journal. In principle, the first author of the primary publication will be the research office, and the principal investigator will be the corresponding author. However, considering the contributions of highly involved investigators, the research office will make a comprehensive decision. Other conference presentations and secondary papers may be made multiple times and will be determined in the same manner. All co-authors must review the content of the manuscript before submission and must have approved the content to be listed as co-authors. If agreement cannot be reached through discussion regarding the content of the manuscript, the principal investigator may exclude the researcher from the list of co-authors.

### 19.3.2. Secondary use of data

In the future, the results of this study may be used as valuable data for further research development. In addition, if the data from this study are to be used for another study, the research plan must be consulted with the Ethical Review Committee and approved before use.

# 20. Efficacy and Safety Evaluation Committee

1. In this study, an efficacy and safety evaluation committee will be established.
2. The committee will be established as an independent body of the principal investigator and will consist of three or more experts who are independent of the study.

During the study period, the patient will be monitored by the Efficacy and Safety Evaluation Committee (e.g., adverse event report, monitoring report review, protocol revision review, etc.).

# 21. Research Organizations

## 21.1. Principal investigator

**Masayuki Watanabe**

Department of Gastroenterological Surgery, Cancer Institute Hospital, Japanese Foundation for Cancer Research

〒135-8550

3-8-31 Ariake, Koto-ku, Tokyo

TEL：03-3520-0111

FAX：03-3570-0343

E-mail：masayuki.watanabe@jfcr.or.jp

## 21.2. Research Secretariat

**Yu Imamura**

Department of Gastroenterological Surgery, Cancer Institute Hospital, Japanese Foundation for Cancer Research

〒135-8550

3-8-31 Ariake, Koto-ku, Tokyo

TEL：03-3520-0111

FAX：03-3570-0343

Email:[yu.imamura@jfcr.or.jp](mailto:yu.imamura@jfcr.or.jp)

**Masayoshi Terayama**

Department of Gastroenterological Surgery, Cancer Institute Hospital, Japanese Foundation for Cancer Research

〒135-8550

3-8-31 Ariake, Koto-ku, Tokyo

TEL：03-3520-0111

FAX：03-3570-0343

E-mail：masayoshi.terayama@jfcr.or.jp

## 21.3. Co-Investigators

Ko Kitazawa Deputy Director, Department of Diabetes, Metabolism and Endocrinology, Cancer Institute Hospital, Japanese Foundation for Cancer Research

Jun Kanamori Department of Gastroenterological Surgery, Cancer Institute Hospital, Japanese Foundation for Cancer Research

Akihiko Okamura Department of Gastroenterological Surgery, Cancer Institute Hospital, Japanese Foundation for Cancer Research

Kengo Kuriyama Department of Gastroenterological Surgery, Cancer Institute Hospital, Japanese Foundation for Cancer Research

Naoki Takahashi Department of Gastroenterological Surgery, Cancer Institute Hospital, Japanese Foundation for Cancer Research

Naohiro Tamura Department of Gastroenterological Surgery, Cancer Institute Hospital, Japanese Foundation for Cancer Research

Misuzu Ishii Department of Nutrition, Cancer Institute Hospital, Japanese Foundation for Cancer Research

Kumi Takagi Department of Nutrition, Cancer Institute Hospital, Japanese Foundation for Cancer Research

## 21.4. Head of Statistical Analysis

Naoki Miyazaki, Planning and Strategy Department, Advanced Cancer Treatment Development Center, Cancer Institute Hospital, Japanese Foundation for Cancer Research

〒135-8550

3-8-31 Ariake, Koto-ku, Tokyo

TEL：03-3520-0111

FAX：03-3570-0343

E-mail：naoki.miyazaki@jfcr.or.jp

## 21.5. Person in charge of monitoring

Yoshiko Matsui, Planning and Strategy Department, Advanced Cancer Treatment Development Center, Ariake Hospital

〒135-8550

3-8-31 Ariake, Koto-ku, Tokyo

TEL：03-3520-0111

FAX：03-3570-0343

E-mail：yoshiko.matsui@jfcr.or.jp

## 21.6. Data Centers

Yoshiko Matsui, Planning and Strategy Department, Advanced Cancer Treatment Development Center, Cancer Institute Hospital, Japanese Foundation for Cancer Research

〒135-8550

3-8-31 Ariake, Koto-ku, Tokyo

TEL：03-3520-0111

FAX：03-3570-0343

E-mail：yoshiko.matsui@jfcr.or.jp

## 21.7. Efficacy and Safety Evaluation Committee

Department of Hepatobiliary and Pancreatic Surgery, Cancer Institute Hospital, Japanese Foundation for Cancer Research

Hiromichi Ito

〒135-8550

3-8-31 Ariake, Koto-ku, Tokyo

TEL：03-3520-0111

FAX：03-3570-0343

[hiromichi.ito@jfcr.or.jp](mailto:hiromichi.ito@jfcr.or.jp)

Department of Gastric Surgery, Cancer Institute Hospital, Japanese Foundation for Cancer

Research

Tomoyuki Irino

〒135-8550

3-8-31 Ariake, Koto-ku, Tokyo

TEL：03-3520-0111

FAX：03-3570-0343

[tomoyuki.irino@jfcr.or.jp](mailto:tomoyuki.irino@jfcr.or.jp)

Department of Colorectal Surgery, Cancer Institute Hospital, Japanese Foundation for Cancer

Research

Shinpei Matsui

〒135-8550

3-8-31 Ariake, Koto-ku, Tokyo

TEL：03-3520-0111

FAX：03-3570-0343

[shimpei.mtsui@jfcr.or.jp](mailto:shimpei.mtsui@jfcr.or.jp)

# 22. References

1. Cancer statistics 2021. National Cancer Center Japan. <https://ganjoho.jp/data/reg_stat/statistics/brochure/2021/cancer_statistics_2021.pdf>
2. Kataoka K, Takeuchi H, Mizusawa J, Igaki H, Ozawa S, Abe T, et al. Prognostic Impact of Postoperative Morbidity After Esophagectomy for Esophageal Cancer: Exploratory Analysis of JCOG9907. Ann Surg. 2017; 265(6):1152-1157.
3. May AK, Kauffmann RM, Collier BR. The place for glycemic control in the surgical patient. SurgInfect (Larchmt). 2011; 12:405-18
4. Hori S, Imamura Y, Watanabe M, et al. Early postoperative hyperglycemia as a predictor of postoperative infectious complications and overall survival in non-diabetic patients with esophageal cancer. J Gatrointest Surg (Accepted).
5. Riddle MC, Gerstein HC, Cefalu WT. Maturation of CGM and Glycemic Measurements Beyond HbA(1c)-A Turning Point in Research and Clinical Decisions. Diabetes Care. 2017; 40(12):1611-3.
6. Laffel LM, Kanapka LG, Beck RW, Bergamo K, Clements MA, Criego A, et al. Effect of Continuous Glucose Monitoring on Glycemic Control in Adolescents and Young Adults With Type 1 Diabetes: A Randomized Clinical Trial. Jama. 2020; 323(23):2388-96.
7. Beck RW, Riddlesworth TD, Ruedy K, Ahmann A, Haller S, Kruger D, et al. Continuous Glucose Monitoring Versus Usual Care in Patients With Type 2 Diabetes Receiving Multiple Daily Insulin Injections: A Randomized Trial. Ann Intern Med. 2017; 167(6):365-74.
8. Battelino T, Danne T, Bergenstal RM, et al. Clinical Targets for Continuous Glucose Monitoring Data Interpretation: Recommendations From the International Consensus on Time in Range. Diabetes Care. 2019; 42(8):1593-603.
9. Beck RW, Bergenstal RM, Cheng P, et al. The Relationships Between Time in Range, Hyperglycemia Metrics, and HbA1c. J Diabetes Sci Technol. 2019; 13(4):614-26.
10. Le Floch JP, Escuyer P, Baudin E, Baudon D, Perlemuter L. Blood glucose area under the curve. Methodological aspects. Diabetes Care. 1990; 13(2):172-5.
11. Siegelaar SE, Holleman F, Hoekstra JB, DeVries JH. Glucose variability; does it matter? Endocr Rev. 2010; 31(2):171-82.
12. Battelino T, Alexander CM, Amiel SA, Arreaza-Rubin G, Beck RW, Bergenstal RM, et al. Continuous glucose monitoring and metrics for clinical trials: an international consensus statement. Lancet Diabetes Endocrinol. 2023; 11(1):42-57.
