# Supplementary material for: Study protocol: The effect of a low-carbohydrate enteral nutrition formula on postoperative hyperglycemia in non-diabetic patients with esophageal cancer: A randomized exploratory phase II trial (ENLICHE study)
Source: PLoS One. 2025 May 28;20(5):e0325039. doi: 10.1371/journal.pone.0325039 (PMC12118858; doi:10.1371/journal.pone.0325039)
Supplement: S2 Study Protocol — (DOCX) [file pone.0325039.s002.docx]

非糖尿病患者における食道癌術後の糖質制限経腸栄養剤投与による

術後高血糖抑制効果を評価するランダム化比較第II相試験

研究計画書

A Randomized Exploratory phase II Trial evaluating the Effect of Enteral Nutrition formula with Low Carbohydrate on Postoperative Hyperglycemia in Non-Diabetic Patients with Esophageal Cancer

(ENLICHE study)

**研究責任医師：渡邊　雅之**

公益財団法人がん研究会有明病院　食道外科

〒135-8550

東京都江東区有明3-8-31

TEL：03-3520-0111 （内線）8504

FAX：03-3570-0343

E-mail：masayuki.watanabe@jfcr.or.jp

**研究事務局 ：今村　裕**

公益財団法人がん研究会有明病院　食道外科

〒135-8550

東京都江東区有明3-8-31

TEL：03-3520-0111 （内線）7436

FAX：03-3570-0343

E-mail：[yu.imamura@jfcr.or.jp](mailto:yu.imamura@jfcr.or.jp)

**：寺山　仁祥**

公益財団法人がん研究会有明病院　食道外科

〒135-8550

東京都江東区有明3-8-31

TEL：03-3520-0111 （内線）8001

FAX：03-3570-0343

E-mail：masayoshi.terayama@jfcr.or.jp

2024年 3月 15日 ver1.0 作成

改訂履歴表

| 改訂番号 | 年月日 | 改訂内容 | 改訂理由 |
| --- | --- | --- | --- |
| 1.0 | 2024.3.15 | 制定 | - |
|  |  |  |  |

# 0. 概要

## 0.1. シェーマ


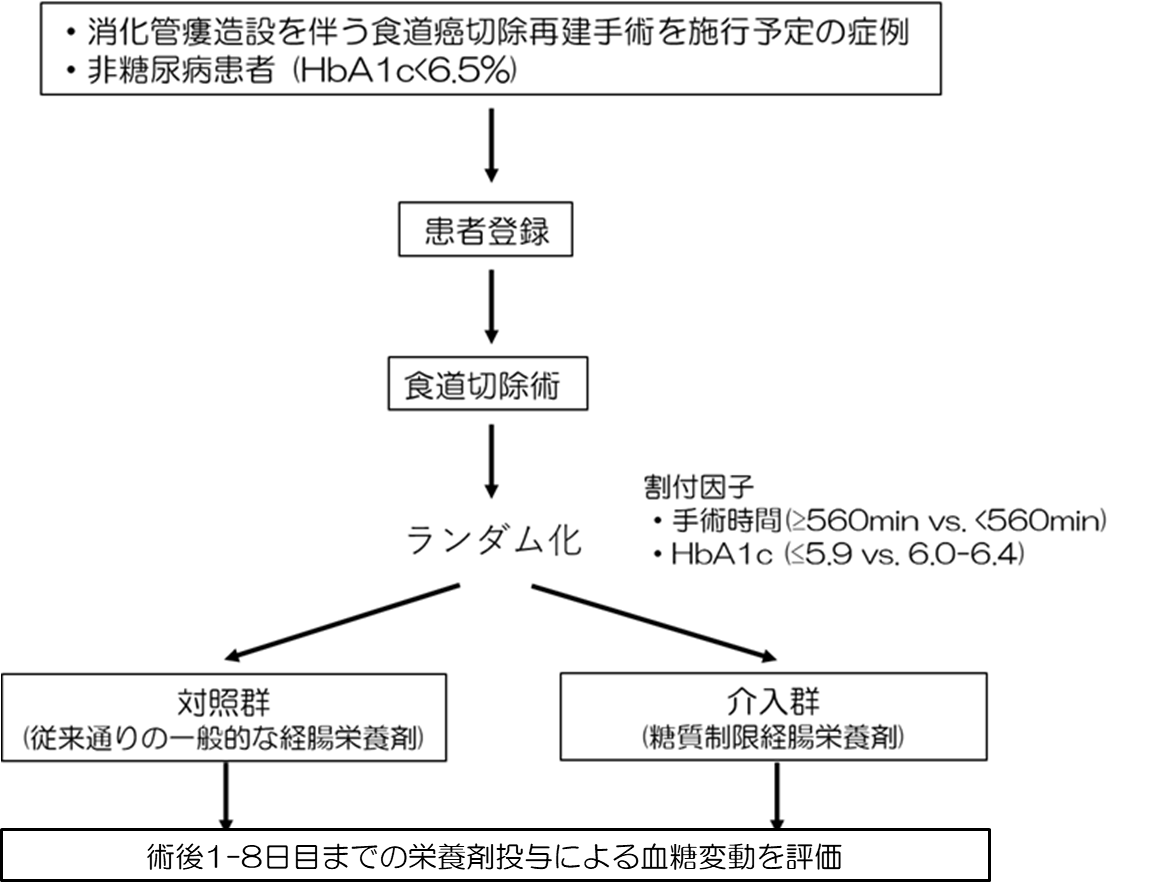


## 0.2. 目的

本試験では、食道癌の術後経腸栄養剤として糖質制限経腸栄養剤であるグルセルナ^®^-REX(アボットジャパン)を使用し、従来の糖質制限のない一般的な経腸栄養剤（明治メイン）と比較して、その高血糖抑制効果を検討することを目的とする。

主要評価項目： 持続血糖測定装置 (Continuous Glucose Monitoring：CGM) で測定した術後

2日目までのTime in range (TIR)の平均値

副次評価項目： 1. 入院期間中の感染性合併症の発生割合

2. 術後30日以内の感染性合併症の発生割合

3. 入院期間中の全合併症の発生割合

4. 有害事象発生割合

5. CGMで測定した全測定期間（術後1-8日目）におけるTARの平均値

6. CGMで測定した術後8日目までの各日毎のTAR

7. CGMで測定した全測定期間（術後1-8日目）におけるAUCの平均値

8. CGMで測定した術後8日目までの各日毎のAUC

9. CGMで測定した全測定期間(術後1-8日目)におけるTIRの平均値

10. CGMで測定した術後8日目までの各日毎のTIR

11. 入院期間中の栄養学的指標の入院時に対する変化率

（血清のアルブミン値、プレアルブミン値、総蛋白値）

12. 高血糖基準(≧300mg/dL)に対する血糖コントロール介入症例数

13. 術後3日目以降の経腸栄養剤変更症例数

14. 術後3日目以降、前日比で50%以上の経腸栄養剤減量を要した症例数

## 0.3. 対象

以下の選択基準をすべて満たし、かつ除外基準のいずれにも該当しない患者を対象とする。

### 0.3.1. 選択基準

1. 組織学的に食道癌と診断されている。組織型は問わない。
2. 糖尿病既往を有さない（初診時HbA1c6.5未満）。
3. 予定手術が胃管再建を伴う食道亜全摘術である。
4. 登録時の年齢が20歳以上である。
5. 本試験内容の十分な説明が行われた上で、試験参加の同意が得られている。
6. 臓器機能が保たれ、全身麻酔に対する耐用性を有する。

### 0.3.2. 除外基準

1. 遠隔転移を有する患者。
2. 緩和的切除を施行した患者。
3. 二期再建を施行した患者。
4. 咽頭喉頭食道全摘を施行した患者。
5. 胸管合併切除を要した患者。
6. 術前化学放射線治療を行った患者。
7. やむを得ない理由によりステロイドの予防投与を行わなかった患者。
8. ペースメーカー等の埋め込み型医療機器を使用している患者。

## 0.4. 治療

食道切除術後１日目から、対照群には一般的な経腸栄養剤である明治メインを、介入群には糖質制限経腸栄養剤であるグルセルナ^®^-REXを、術後1日目400mL、術後2日目800mL、術後3日目1200mL、術後4日目以降1600mLで24時間持続投与する。術後4-8日目においては1600mLのまま継続する。術後9日目には、通常の術後管理へ切り替え、両群ともに濃厚流動食品であるハイネックス_®_イーゲル1000ｍLへ切り替え、食事摂取を開始する。

## 0.5. 予定登録数と研究期間

予定登録患者数：計50名 (対照群 25例、介入群 25例)

登録期間：jRCT公開～1.5年

追跡期間：登録終了後0.5年

解析期間：登録終了後1年

総研究期間：2.5年

## 0.6. 問い合せ先

【患者登録の連絡先および患者選択規準に関する問い合わせ先】

- 研究事務局：今村 裕

公益財団法人がん研究会有明病院　食道外科

〒135-8550　東京都江東区有明3-8-31

TEL:03-3520-0111 (内線7436)　FAX:03-3570-0343

E-mail: yu.imamura@jfcr.or.jp

- 研究事務局：寺山 仁祥

公益財団法人がん研究会有明病院　食道外科

〒135-8550　東京都江東区有明3-8-31

TEL:03-3520-0111 (内線8001)　FAX:03-3570-0343

E-mail: masayoshi.terayama@jfcr.or.jp

【EDCの操作など臨床的判断を有さない問い合わせ先】

- データセンター：松井 美子

公益財団法人がん研究会有明病院　先進がん治療開発センター　企画戦略部

〒135-8550　東京都江東区有明3-8-31

TEL：03-3520-0111 (内線7512)　FAX:：03-3570-0701

E-mail：yoshiko.matsui@jfcr.or.jp

# 目次

[0. 概要 3](#_Toc161155319)

[0.1. シェーマ 3](#_Toc161155320)

[0.2. 目的 3](#_Toc161155321)

[0.3. 対象 4](#_Toc161155322)

[0.3.1. 選択基準 4](#_Toc161155323)

[0.3.2. 除外基準 4](#_Toc161155324)

[0.4. 治療 4](#_Toc161155325)

[0.5. 予定登録数と研究期間 4](#_Toc161155326)

[0.6. 問い合せ先 4](#_Toc161155327)

[目次 6](#_Toc161155328)

[1. 目的 9](#_Toc161155329)

[2. 背景と試験計画の根拠 9](#_Toc161155330)

[2.1. 背景 9](#_Toc161155331)

[2.2. 当科の現状 10](#_Toc161155332)

[2.2.1 経腸栄養剤の現状 10](#_Toc161155333)

[2.2.2. 当科の血糖測定方法および血糖管理の現状 10](#_Toc161155334)

[2.3. 試験介入の概要 11](#_Toc161155335)

[2.3.1. 経腸栄養剤 11](#_Toc161155336)

[2.3.2. 栄養剤の組成と特性 11](#_Toc161155337)

[2.3.3. FreeStyleリブレPro 12](#_Toc161155338)

[2.4. 試験デザイン 12](#_Toc161155339)

[2.4.1. CGMを用いた血糖測定 12](#_Toc161155340)

[2.4.2. 臨床的仮説 13](#_Toc161155341)

[2.4.3. 患者登録見込み 13](#_Toc161155342)

[2.5. 試験参加に伴って予想される利益と不利益の要約 14](#_Toc161155343)

[2.5.1. 予想される利益 14](#_Toc161155344)

[2.5.2. 予想される負担と不利益 14](#_Toc161155345)

[2.6. 本試験の意義 14](#_Toc161155346)

[2.7. 附随研究 14](#_Toc161155347)

[3. 本試験で用いる基準・定義 14](#_Toc161155348)

[3.1. 感染性合併症 14](#_Toc161155349)

[3.2 食道癌の診断基準 14](#_Toc161155350)

[4. 患者適格基準 14](#_Toc161155351)

[4.1. 選択基準 15](#_Toc161155352)

[4.2. 除外基準 15](#_Toc161155353)

[5. 登録・割付 15](#_Toc161155354)

[5.1. 登録の手順 15](#_Toc161155355)

[5.1.1. 登録に関しての注意事項 16](#_Toc161155356)

[5.2. 割付と割付因子 16](#_Toc161155357)

[6. 治療計画と治療変更基準 17](#_Toc161155358)

[6.1. プロトコル治療 17](#_Toc161155359)

[6.1.1. 使用する経腸栄養剤: 17](#_Toc161155360)

[6.1.２. 投与スケジュール 、用量・用法 17](#_Toc161155361)

[6.1.3. 患者の血糖管理 17](#_Toc161155362)

[6.2. プロトコル治療完了・中止・変更基準 18](#_Toc161155363)

[6.2.1. プロトコル治療完了の定義 18](#_Toc161155364)

[6.2.2. プロトコル治療中止の基準 18](#_Toc161155365)

[6.2.3. プロトコル治療の変更基準 18](#_Toc161155366)

[6.3. 併用療法・支持療法 18](#_Toc161155367)

[6.3.1. 高血糖・低血糖に対する許容される併用・支持療法 18](#_Toc161155368)

[6.3.2. その他の許容される併用・支持療法 19](#_Toc161155369)

[6.3.2. 許容されない併用療法・支持療法 19](#_Toc161155370)

[7. 予期される有害事象 19](#_Toc161155371)

[7.1. 予期される有害事象・不具合 19](#_Toc161155372)

[7.1.1. 経腸栄養剤投与(明治メイン、グルセルナ^®^-REXともに)に伴う有害事象 19](#_Toc161155373)

[7.1.2. 食道切除術に伴う有害事象 19](#_Toc161155374)

[7.1.3. FreeStyleリブレPro使用に伴う重大な有害事象/有害事象 19](#_Toc161155375)

[7.1.4. FreeStyleリブレPro使用に伴う不具合 20](#_Toc161155376)

[7.2. 有害事象/有害反応の評価 20](#_Toc161155377)

[7.3. 有害事象観察期間 20](#_Toc161155378)

[7.4. 因果関係の判定 20](#_Toc161155379)

[8. 評価項目 20](#_Toc161155380)

[8.1. 登録前(手術日より28日前以内)の評価項目 20](#_Toc161155381)

[8.2. 手術当日の評価項目 21](#_Toc161155382)

[8.3. 手術後1日目の評価項目 21](#_Toc161155383)

[8.4. 手術後2～3日目の評価項目 21](#_Toc161155384)

[8.5. 手術後4～8日目の評価項目 21](#_Toc161155385)

[8.6. 手術後9日目の評価項目 22](#_Toc161155386)

[8.7. 退院時^※（^後観察）の評価項目：許容範囲　退院日±7日 22](#_Toc161155387)

[8.8. 明治メインおよびグルセルナ^®^-REX の最終投与30日後（後観察）の評価項目：許容範囲+14日 22](#_Toc161155388)

[8.9. スタディカレンダー 23](#_Toc161155389)

[9. 有害事象の報告 25](#_Toc161155390)

[9.1. 報告義務のある有害事象 25](#_Toc161155391)

[9.2. 有害事象又は疾病等が発生した場合の報告義務と報告手順 26](#_Toc161155392)

[9.2.1. 本試験に従事する者の責務 26](#_Toc161155393)

[9.2.2. 研究事務局の責務 26](#_Toc161155394)

[9.2.3. 研究責任医師および研究事務局の責務 26](#_Toc161155395)

[9.2.4. 効果・安全性評価委員会への報告 26](#_Toc161155396)

[9.2.5. 効果・安全性評価委員会の責務 26](#_Toc161155397)

[9.2.6. 認定臨床研究審査委員会および厚生労働大臣への報告 26](#_Toc161155398)

[9.2.7. 定期報告 26](#_Toc161155399)

[10. 効果判定とエンドポイントの定義 27](#_Toc161155400)

[10.1. 評価項目 27](#_Toc161155401)

[11. 統計的事項 27](#_Toc161155402)

[11.1. 解析対象集団の定義 27](#_Toc161155403)

[11.２. データ欠測の扱い 28](#_Toc161155404)

[11.３. 主たる解析 28](#_Toc161155405)

[11.4. 副次評価項目（有効性） 28](#_Toc161155406)

[11.5. 副次評価項目（安全性） 28](#_Toc161155407)

[11.6. 予定登録数 29](#_Toc161155408)

[11.7. 中間解析 29](#_Toc161155409)

[11.8. 有意水準及び多重性 29](#_Toc161155410)

[11.9. 最終解析 29](#_Toc161155411)

[12. 研究全体の中止 29](#_Toc161155412)

[12.1. 研究全体の中止基準 29](#_Toc161155413)

[12.2. 臨床研究の中止の手続き 30](#_Toc161155414)

[13. 倫理的事項 30](#_Toc161155415)

[13.1. 患者の保護 30](#_Toc161155416)

[13.2. インフォームドコンセント 30](#_Toc161155417)

[13.2.1. 患者への説明 30](#_Toc161155418)

[13.2.2. 同意 31](#_Toc161155419)

[13.2.3. 同意撤回 31](#_Toc161155420)

[13.3. 個人情報 32](#_Toc161155421)

[13.3.1. 従うポリシー、法令、規範 32](#_Toc161155422)

[13.3.2. 個人情報の保護と患者識別 32](#_Toc161155423)

[13.3.3. 個人情報の利用目的と利用する項目、及び利用方法 32](#_Toc161155424)

[13.4. 原資料 32](#_Toc161155425)

[13.5. 試料・情報などの保管 33](#_Toc161155426)

[13.6.補償について 33](#_Toc161155427)

[13.7. 知的財産について 33](#_Toc161155428)

[13.8. プロトコルの遵守 33](#_Toc161155429)

[13.9. CRBへの申請及び実施計画の届出 33](#_Toc161155430)

[14. 定期報告 33](#_Toc161155431)

[15. 臨床研究に関わる者の利益相反(COI)の管理について 34](#_Toc161155432)

[16. CRBに承認された書類の内容変更について 34](#_Toc161155433)

[16.1 実施計画の変更 34](#_Toc161155434)

[16.2 プロトコールの内容変更について 34](#_Toc161155435)

[16.3 同意説明文書の内容変更及び、患者説明・再同意について 34](#_Toc161155436)

[16.4 その他の承認が得られた書類について 35](#_Toc161155437)

[17. 不適合の管理 35](#_Toc161155438)

[17.1. 不適合 35](#_Toc161155439)

[17.2. 重大な不適合 35](#_Toc161155440)

[17.3. 不適合報告の手順 35](#_Toc161155441)

[18. モニタリングと監査 36](#_Toc161155442)

[18.1. 定期モニタリング 36](#_Toc161155443)

[18.2. 監査 36](#_Toc161155444)

[19. 研究全体の終了 36](#_Toc161155445)

[19.1. 研究の終了 36](#_Toc161155446)

[19.2. 研究終了の手順 36](#_Toc161155447)

[19.3. 研究結果の扱い 37](#_Toc161155448)

[19.3.1. 研究結果の公表 37](#_Toc161155449)

[19.3.2. データの二次利用 37](#_Toc161155450)

[20. 効果・安全性評価委員会 37](#_Toc161155451)

[21. 研究組織 38](#_Toc161155452)

[21.1. 研究責任医師 38](#_Toc161155453)

[21.2. 研究事務局 38](#_Toc161155454)

[21.3. 研究分担者 38](#_Toc161155455)

[21.4. 統計解析責任者 39](#_Toc161155456)

[21.5. モニタリング責任者 39](#_Toc161155457)

[21.6. データセンター 39](#_Toc161155458)

[21.7. 効果・安全性評価委員会 39](#_Toc161155459)

[22. 参考文献 40](#_Toc161155460)

# 1. 目的

本試験では、食道癌に対する食道切除術を受けた患者を対象に，糖質制限経腸栄養剤グルセルナ^®^-REX（アボットジャパン）の高血糖抑制効果を，糖質制限のない一般的な経腸栄養剤（明治メイン）を対照に比較検討することを目的とする。

主要評価項目： 持続血糖測装置(Continuous Glucose Monitoring: CGM)で測定した術後2日目までのTime in range (TIR)の平均値

副次評価項目：1. 入院期間中の感染性合併症の発生割合

2. 術後30日以内の感染性合併症の発生割合

3. 入院期間中の全合併症の発生割合

4. 有害事象発生割合

5. CGMで測定した全測定期間（術後1-8日目）におけるTARの平均値

6. CGMで測定した術後8日目までの各日毎のTAR

7. CGMで測定した全測定期間（術後1-8日目）におけるAUCの平均値

8. CGMで測定した術後8日目までの各日毎のAUC

9. CGMで測定した全測定期間(術後1-8日目)におけるTIRの平均値

10. CGMで測定した術後8日目までの各日毎のTIR

11.入院期間中の栄養学的指標の入院時に対する変化率

（血清のアルブミン値、プレアルブミン値、総蛋白値）

12. 高血糖基準(≧300mg/dL)に対する血糖コントロール介入症例数

13. 術後3日目以降の経腸栄養剤変更症例数

14. 術後3日目以降、前日比で50%以上の経腸栄養剤減量を要した症例数

# 2. 背景と試験計画の根拠

## 2.1. 背景

食道癌は、5年生存率が40.6%と依然として予後不良な悪性腫瘍である[1］。近年の医療技術の進歩により、手術単独の他、化学療法や放射線療法を組み合わせた集学的治療による予後改善が期待されているが、手術は依然として根治を目指すことの可能な唯一の治療法である。しかし、食道癌手術は消化器外科手術のなかでも術後合併症の発生頻度が高いことで知られる。術後合併症の中でも、感染性合併症の頻度は高く、肺炎や縫合不全といった代表的な感染性合併症は重篤化しやすい上、長期予後にも影響する。従って、術後感染性合併症の発症リスクを軽減することは食道癌の診療上きわめて重要な課題である[2]。当院で2014年～2018年に食道癌に対して食道切除・再建術を施行した患者430名のうち，約3割（127名）に術後感染性合併症が認められた。この原因として、外科手術後の高血糖が、好中球の走化性・貪食能に機能低下を来し、術直後の炎症性サイトカインの増加と相まって感染性合併症のリスクとなることが分かっており、特に糖尿病合併症例では術後の感染性合併症のリスクが高いことが知られている[３]。

我々は、本試験に先立ち食道癌の術後血糖と感染性合併症の関係性を後方視的に検討し、糖尿病患者のみならず非糖尿病患者においても、術後の高血糖が感染性合併症を増加させること、更に術後侵襲が術直後の血糖上昇に寄与することを報告した [4]。術後平均血糖値と術後感染性合併症の関連性の検討結果では、術後1日目の平均血糖が200mg/dL以上であった高血糖群において、平均血糖が200mg/dL以下であった通常血糖群（高血糖なし群）と比較して、感染性合併症の発生頻度が有意に高いことが示された（高血糖群39.3％ vs. 高血糖なし群25.6%, *P*=0.0068）。サブグループ解析の結果では、この傾向は糖尿病症例(高血糖群39.3％ vs. 高血糖なし群25.6%, *P* =0.0068)と同様に、非糖尿病症例においても顕著に認められた（高血糖群42.9％ vs. 高血糖なし群25.2%, *P* =0.0022）。また、サブグループ別の多変量解析では、術後1, 2, 4日目における平均血糖が200mg/dLの高血糖状態が、非糖尿病症例における感染性合併症の独立した危険因子であることを報告した[高血糖なし群に対する高血糖群の多変量ハザード比: 術後1日目1.94, 術後2日目 3.68, 術後4日目 3.07]。

術後高血糖になる1つの要因が、栄養管理である。食道癌の術後は、十分な経口食事摂取が可能となるまで時間を要するため、術直後より消化管瘻を介した半消化態栄養剤を用いる経腸栄養管理が行われることが多い。その際、経腸栄養剤は、その目的に合わせて適切に選択することが重要である。特に糖尿病症例では、周術期血糖の乱高下を抑制する目的で、一般的な経腸栄養剤と比して低炭水化物・高脂質で組成される糖質制限栄養剤が望ましい。本試験にて使用する経腸栄養剤グルセルナ^®^-REXは、糖質制限経腸栄養剤として開発され、糖質のエネルギー比率が25％に制限された低糖質タイプの経腸栄養剤であり（一般的な経腸栄養剤(明治メイン)では糖質のエネルギー比率が60%）、その代わりにエネルギー源として脂質を50%へ増やしている。ただしその脂質は、約90％以上がオレイン酸、リノール酸、α-リノレン酸などの不飽和脂肪酸組成であり、飽和脂肪酸に比べ、脂質代謝に悪影響を与えにくい脂肪酸で構成されている。従って、一般的な経腸栄養剤(明治メイン)では術後の血糖管理が難しい場合においても、グルセルナ^®^-REXを用いることで、一般的な経腸栄養剤よりも血糖上昇を抑える効果が期待されることから、実臨床では既に糖尿病患者の術後経腸栄養剤として広く用いられている。

しかしながら、非糖尿病患者に対するグルセルナ^®^-REXを用いた術後栄養管理は、これまで一般的ではなく、グルセルナ^®^-REXが食道癌手術を受ける非糖尿病患者において、術後早期の血糖上昇を抑制できるかどうかは明らかにされていない。そこで今回我々は、食道癌術後の非糖尿病病患者において、グルセルナ^®^-REXが術後早期の血糖上昇を抑制できるかどうかを、一般的な半消化熊栄養剤である明治メインを対照として比較検討する目的で、単施設、非盲検、ランダム化比較試験を計画することとした。なお、本試験では、術後の血糖値測定に、24時間の血糖変動を可視化した持続血糖測定装置(continuous glucose monitoring: CGM)であるFreeStyleリブレProを用いる[5-7]。CGMを使用することで、血糖変動の詳細を把握することが出来るため、HbA1cや定時血糖測定といった、従来の間欠的測定では検出できない潜在的な高血糖や低血糖を、正確かつ詳細に把握することが可能となる。グルセルナ^®^-REXだけでなく、従来の一般的な明治メイン投与後の血糖変動自体、これまで報告されておらず、そのデータ取得も本研究の目的である。

## 2.2. 当科の現状

### 2.2.1 経腸栄養剤の現状

当科では、従来、非糖尿病患者の術後経腸栄養剤として一般的な半消化態栄養剤である明治メインを用い、基本的に術後1日400mL、術後2日目800mL、術後3日目1200mL、術後4日目以降1600mLで投与し、術後9日目には濃厚流動食品であるハイネックス_®_イーゲル1000ｍLへ切り替え、食事が開始される。食事摂取開始後は摂取量に合わせて漸減している。なお、明治メインの投与量、ハイネックス_®_イーゲルへの切り替えおよび食事開始時期については、患者状態により適宜変更する。

### 2.2.2. 当科の血糖測定方法および血糖管理の現状

血糖測定については、集中治療室(Intensive Care Unit: 以下ICU)在室期間中は橈骨動脈に挿入されている動脈ラインより採取した血液ガスで、6時間おきに血糖値を測定している。術後4日目以降の一般病棟在室時においては、それまでの血糖推移に応じて、介入が必要と判断された場合において、指先での間欠的血糖測定(通常毎食前眠前の4回)を行っている。

血糖値に対する介入（高血糖および低血糖に対するレスキュー治療）は、下記のとおりである。

| 血糖値 (mg/dL) | | 即効型インスリン | 50%ブドウ糖 |
| --- | --- | --- | --- |
| 高血糖 | 201以上～245以下 | 2単位 | - |
|  | 250以上～299以下 | 4単位 | - |
|  | 300以上～349以下 | 6単位 | - |
|  | 350以上～399以下 | 8単位 | - |
|  | 400以上 | 10単位 | - |
| 低血糖 | 69以下 | - | 20mL |

当科の現状の術後管理

| 場所 　　　　　　　　時期  項目 | 手術室 | ICU | | | 一般病棟 | | |
| --- | --- | --- | --- | --- | --- | --- | --- |
|  | Day0 | Day1 | Day2 | Day3 | Day4-8 | Day9 | Day10以降 |
| 手術 | ○ |  |  |  |  |  |  |
| 明治メイン |  | 400mL | 800mL | 1200mL | 1600mL |  |  |
| ハイネックス_®_イーゲル |  |  |  |  |  | 1000mL | 適宜漸減 |
| 食事摂取 |  |  |  |  |  | ○ | ○ |
| 血糖測定（4回/日） |  | ○ | ○ | ○ | ○ | ○ | ○ |
| 血糖値に対する介入 |  | ○ | ○ | ○ | ○ | ○ | ○ |

## 2.3. 試験介入の概要

### 2.3.1. 経腸栄養剤

本試験にて使用するグルセルナ^®^-REXは、糖質制限経腸栄養剤として開発され、既に実臨床で広く使用されている。一般的な経腸栄養剤では糖質のエネルギー比率が60％で組成されているのに対し、グルセルナ^®^-REXは25％に制限された低糖質タイプの経腸栄養剤である（明治メインに比して糖質が66％に制限）。その代わりにエネルギー源として脂質を50%へ増やしている。ただしその脂質は、約90％以上がオレイン酸、リノール酸、α-リノレン酸などの不飽和脂肪酸組成であり、飽和脂肪酸に比べ、脂質代謝に悪影響を与えにくい脂肪酸で構成されている。従って、グルセルナ^®^-REXを用いることで、一般的な経腸栄養剤よりも血糖上昇を抑える効果が期待される。

### 2.3.2. 栄養剤の組成と特性

以下に、本試験で使用する2種類の経腸栄養剤の組成表を示す。

本試験において、両群へ投与する総カロリーは同じである。

|  | 単位 | 従来型経腸栄養剤  (明治メイン) | 試験経腸栄養剤  (グルセルナ^®^-REX) |
| --- | --- | --- | --- |
| 容量 | mL | 100 | 100 |
| エネルギー | kcal | 100 | 100 |
| タンパク質 | g | 5 | 4.2 |
| 脂質 | g | 2.8 | 5.6 |
| 炭水化物 | g | 15 | 9.7 |
| 糖質 | g | 13.2 | 8.8 |
| 食物繊維 | g | 1.8 | 0.9 |
| 水分 | g | 84.1 | 85 |
| ビタミンA | μgRAE | 150 | 104 |
| ビタミンB1 | mg | 0.25 | 0.12 |
| ビタミンB2 | mg | 0.3 | 0.18 |
| ビタミンB12 | μg | 0.6 | 0.3 |
| ビタミンD | μg | 0.75 | 0.9 |
| 葉酸 | μg | 50 | 20 |
| ビタミンC | mg | 50 | 11 |
| ナトリウム | mg | 80 | 94 |
| カリウム | mg | 120 | 100 |
| カルシウム | mg | 100 | 70 |
| 浸透圧 | mOsm/L | 640 | 560 |

### 2.3.3. FreeStyleリブレPro

本試験で使用するFreeStyleリブレProは血糖測定センサーとReaderが含まれている（図1）。通常、センサーは快適さや活動のしやすさを考慮し、上腕の外側部位に装着する。この際、傷跡、ほくろ、ストレッチマークまたは瘤のある場所や、インスリン注射の部位は避ける。本試験では、術直後病棟に帰室した段階で、研究分担医師がセンサーを患者の上腕外側に貼付し、術後1-8日目において、血糖を持続的に測定する。術後9日目に、専用のReaderを用いてスキャンを行い、データを抽出する。その後、センサーは廃棄する。

なお、術後ルーチンで行うレントゲン撮影時には、センサーは剥がさなくともよいものとする。

**【図1】本**試験**で用いる持続血糖測定装置(CGM)：FreeStyleリブレPro (アボットジャパン)**

（出典：<https://www.myfreestyle.jp/hcp/>）


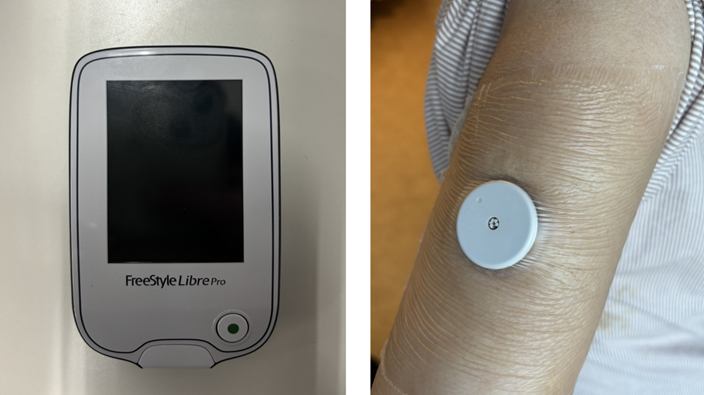


## 2.4. 試験デザイン

本試験は、単施設、非盲検、ランダム化並行群間比較試験として実施する。

### 2.4.1. CGMを用いた血糖測定

糖質制限経腸栄養剤であるグルセルナ^®^-REXの効果を確認するために、血糖上昇抑制作用の指標としてCGMで得られる下記の項目を評価する。

主要評価項目：Time in Range (TIR)

TIR：24時間の血糖値の経時的測定のうち、血糖値が70～180mg/dLの治療域

（target range）内にある時間の割合と定義される（図2の赤枠内に曲線がある時間

(h)/24h）。

副次評価項目：Time Above Range (TAR)、Area Under Curve (AUC)

TAR：24時間の血糖値の経時的測定のうち、血糖≧180mg/dL以上の高血糖域の時

間割合(図2内の①の総和(h)/24h) と定義[7]

AUC：24時間の血糖値の経時的測定により得られる血糖変動曲線における、血糖

≧180mg/dL以上の曲線下面積(図2内の斜線部分②の総和)と定義[9]

・TIRを主要評価項目とした根拠TIRは、2019年6月に開催された米国糖尿病学会学術集会において“血糖管理目標に関する国際的なコンセンサス”として発表された新たな血糖管理指標である[8]。この指標は、従来血糖管理の指標とされてきたHbA1c値と対応することが知られており、TIRが10%増加するごとにHbA1c値が約0.5%減少する（図2）[9]。加えて、間欠的測定では知り得ない、隠れた高血糖や低血糖を検出でき、正確かつ詳細な指標として昨今注目されていることから、本研究の評価項目として妥当と判断した。

**【図2】持続血糖測定装置(CGM)による血糖データの解釈**

（出典：Battelino T　et al. Diabetes Care. 2019［7］）


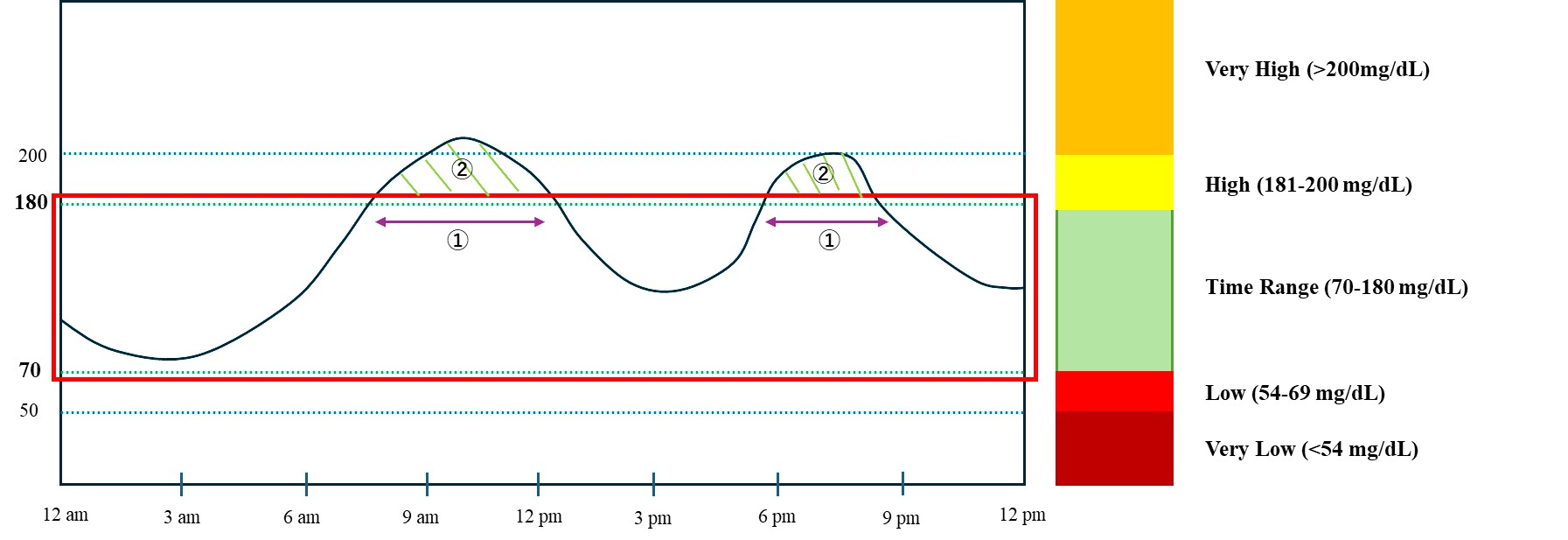


**【図3】持続血糖測定装置(continuous glucose monitoring: CGM)で得られるTime in range(TIR)rate(%)とHbA1cの対応表**

（出典：小出ら　「いま読んでおきたい血糖データの活かし方（南山堂）」2020）


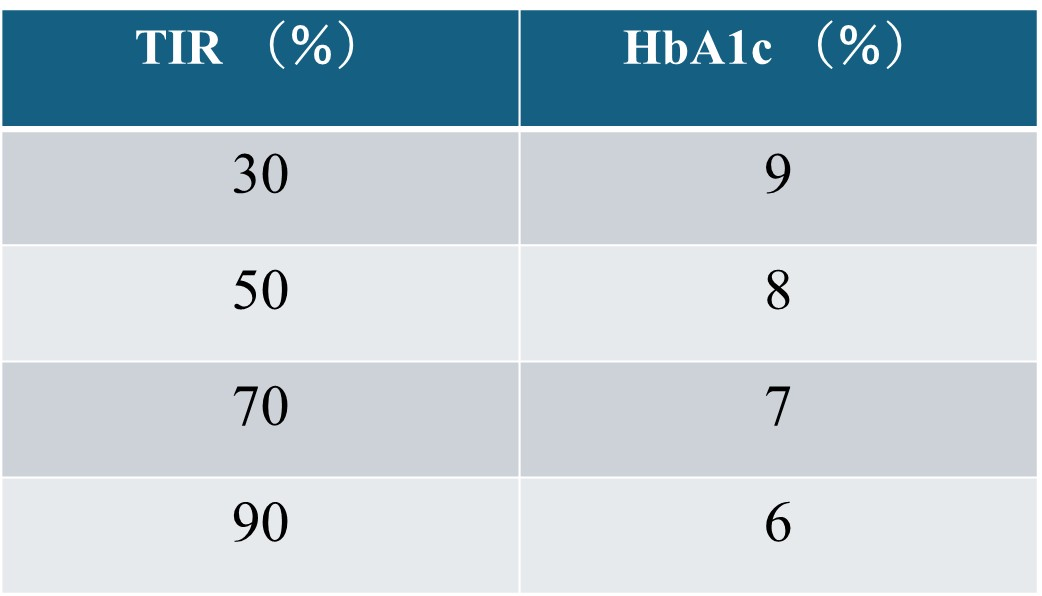


### 2.4.2. 臨床的仮説

本試験の臨床的仮説は、「グルセルナ^®^-REXは、非糖尿病患者における食道癌術後の血糖上昇を抑制する」である。本試験では目標症例数を５０例とする。なお、統計学的仮説に基づく症例数の設定は困難であり、設定研究期間に収集可能な登録症例数とした。脱落を見込み、48例を対象にTIRの群間差の標準偏差を8.5%とすると信頼区間幅±5%程度でTIRの群間差の平均値を推定できる。

### 2.4.3. 患者登録見込み

現在がん研有明病院食道外科では、食道癌に対する切除・再建手術を年間110例/年程度行っている。除外症例も考慮し、jRCT公開後～1.5年以内に症例登録が完了する見込みである。

予定登録数：50名 (25名／群)

登録期間：jRCT公開～1.5年

追跡期間：登録終了後0.5年

解析期間：登録終了後1.0年

総研究期間：2.5年

## 2.5. 試験参加に伴って予想される利益と不利益の要約

### 2.5.1. 予想される利益

研究段階であるため、基本的には期待される利益はない。

### 2.5.2. 予想される負担と不利益

本試験に使用するグルセルナ^®^-REX、明治メインおよびFreeStyleリブレProは、2.1に示す通り既に日常診療で広く使用されている。グルセルナ^®^-REX及び明治メインは食事として提供され、FreeStyleリブレProのセンサーとReaderは共に食道外科研究費より購入する。従って、患者が本試験に参加することにより、特別な身体的負担および経済上の不利益はない。

本試験のプロトコルでは、血液検査の回数や項目が通常の術後管理に比して増えることはない。しかし、FreeStyleリブレProのセンサーの装着にあたり、粘着剤の影響で装着部位やその周囲に皮膚の炎症を生じる可能性が稀にある。

## 2.6. 本試験の意義

本試験の意義は、糖質制限経腸栄養剤グルセルナ^®^-REXを用いた栄養管理が、糖質制限のない一般的な経腸栄養剤（明治メイン）と比較して、非糖尿病患者における食道癌術後の血糖上昇を抑制効果がより高いかどうかを明らかにし，その結果をもって次相の検証的試験での臨床仮説を生成することである。

## 2.7. 附随研究

本試験では付随研究は行わない。

# 3. 本試験で用いる基準・定義

## 3.1. 感染性合併症

感染性合併症の評価には「有害事象共通用語規準v5.0日本語訳JCOG版（NCI-Common Terminology Criteria for Adverse Events v5.0（CTCAE v5.0）の日本語訳）」（以下、CTCAE v5.0-JCOG）を用いる。有害事象のgradingに際しては、それぞれGrade 0～4の定義内容にもっとも近いものへ判定する。

## 3.2 食道癌の診断基準

食道癌治療に関連する事項については、食道癌診療ガイドライン(2022年度版)に準拠する。

# 4. 患者適格基準

本研究の対象は，公益財団法人がん研究会有明病院食道外科を受診し，経腸栄養ルート（胃管瘻、十二指腸瘻、空腸瘻）増設を伴う食道切除術を施行される患者で，以下の4.1選択基準をすべて満たし，かつ4.2除外基準のいずれにも該当しない患者である。

## 4.1. 選択基準

1. 組織学的に食道癌と診断されている。組織型は問わない。
2. 糖尿病既往を有さない（初診時HbA1c6.5未満）。
3. 予定手術が胃管再建を伴う食道亜全摘術である。
4. 登録時の年齢が20歳以上である。
5. 本試験内容の十分な説明が行われた上で、本人の自由意思により 試験参加の同意が得られている。
6. 臓器機能が保たれ、全身麻酔に対する耐用性を有する。

## 4.2. 除外基準

1. 遠隔転移を有する患者。
2. 緩和的切除を施行した患者。
3. 二期再建を施行した患者。
4. 咽頭喉頭食道全摘を施行した患者。
5. 胸管合併切除を要した患者。
6. 術前化学放射線治療を行った患者。
7. やむをえない理由によりステロイドの予防投与を行わなかった患者。
8. ペースメーカー等の埋め込み型医療機器を使用している患者。

主な選択・除外基準の設定根拠：

除外基準⑦ステロイドの予防投与未施行患者を除外する理由について

通常、術後の全身性炎症反応症候群の予防目的で、執刀前にステロイド(ソルメドロール250mg)を投薬しているが、ステロイド投与の有無が術後血糖変動に影響を及ぼす可能性があるため、除外とした。

# 5. 登録・割付

## 5.1. 登録の手順

本試験の選択基準を全て満たし、全ての除外基準に該当しないことを確認し、患者同意を取得の上、がん研究会が管理する臨床情報収集システムElectronic Data Capture (EDC)に必要事項を全て入力し登録する。

EDCシステム：Viedoc

URL：https://v4jp.viedoc.net/Login

【患者登録の連絡先、患者選択基準に関する問い合わせ先】

研究事務局**：** 今村　裕

公益財団法人がん研究会有明病院　食道外科

〒135-8550

東京都江東区有明3-8-31

TEL：03-3520-0111

FAX：03-3570-0343

E-mail：[yu.imamura@jfcr.or.jp](mailto:yu.imamura@jfcr.or.jp)

【EDCの操作など臨床的判断を有さない問い合わせ先】

データセンター：松井 美子

公益財団法人がん研究会有明病院　先進がん治療開発センター　企画戦略部

〒135-8550

東京都江東区有明3-8-31

TEL：03-3520-0111 (内線7512)

FAX:：03-3570-0701

### 5.1.1. 登録に関しての注意事項

プロトコル治療開始後の登録は例外なく許容されない。

入力データが不十分な時は、すべて満たされるまで登録は受け付けられない。登録番号が発行されたことをもって、登録完了とする。

データの研究利用の拒否を含む同意撤回があった場合を除いて、一度登録された患者は登録取り消し（データベースから抹消）はなされない。重複登録の場合は、いかなる場合も初回の登録情報（登録番号、割付群）を採用する。

誤登録・重複登録が判明した際には速やかに研究事務局に連絡する**。**

## 5.2. 割付と割付因子

EDCシステムへ症例を登録後、EDCシステムにより、**いずれ**かの群へランダム割付される。

割付因子は、主要評価項目への影響が特に懸念される、手術時間（≧560 min or ＜560 min）、HbA1c値（HbA1c 6.0未満 or 6.1-6.4）とし大きな偏りが生じないように最小化法を用いる。なお、割付因子の詳細は患者に対し盲検化する。

# 6. 治療計画と治療変更基準

## 6.1. プロトコル治療

### 6.1.1. 使用する経腸栄養剤:

食道癌術後に、経腸栄養剤として対照群には明治メインを、介入群にはグルセルナ^®^-REXを使用する。

グルセルナ^®^-REX(アボットジャパン),乳白色液体状

明治メイン(明治),乳白色液体状


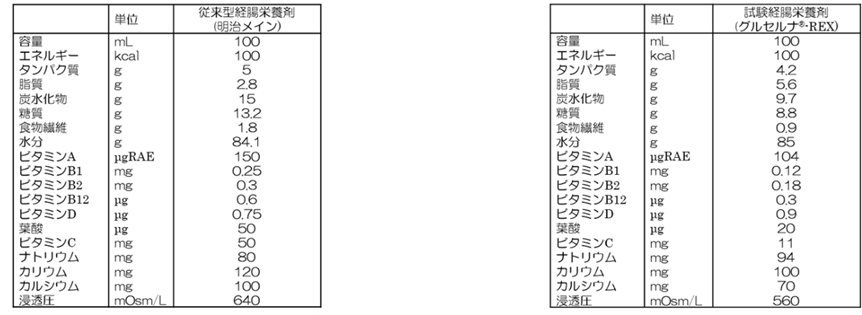


### 6.1.２. 投与スケジュール 、用量・用法

食道切除術後１日目から、対照群には一般的な経腸栄養剤である明治メインを、介入群には糖質制限経腸栄養剤であるグルセルナ^®^-REXを、術後1日目400mL、術後2日目800mL、術後3日目1200mL、術後4日目以降1600mLで24時間持続投与する。術後4-8日目においては1600mLのまま継続する。術後9日目には、通常の術後管理へ切り替え、両群ともに濃厚流動食品であるハイネックス_®_イーゲル1000ｍLへ切り替え、食事摂取を開始する。

### 6.1.3. 患者の血糖管理

本試験においては、術直後病棟に帰室後、研究分担医師が患者の上腕の外側部位にFreeStyleリブレProを貼付しCGMモニタリングを実施する。研究分担医師は、術後9日目に患者よりFreeStyleリブレProを外す。加えて、術直後病棟に帰室以降、1日4回の血液ガスもしくはデキスターによる定時血糖測定を行う。

高血糖および低血糖に対するレスキュー治療に用いる血糖管理は、血液ガスもしくはデキスターによる定時測定で得られた血糖値に対して行う。FreeStyleリブレProで測定した血糖値情報は、高血糖および低血糖に対するレスキュー治療を行うかどうかを決める際の指標値とはしない。

高血糖および低血糖に対するレスキュー治療は、下記のとおりとする。

なお、術後2日目までは、血糖値201-299mg/dLに対するレスキュー治療は許容しない。

術後の高血糖および低血糖に対するレスキュー治療の基準

| 血糖値 (mg/dL) | | 時期 | 即効型インスリン | 50%ブドウ糖 |
| --- | --- | --- | --- | --- |
| 高血糖 | 201以上～249以下 | 術後2日目まで | 不可 | - |
|  |  | 術後3日目以降 | 2単位 | - |
|  | 250以上～299以下 | 術後2日目まで | 不可 | - |
|  |  | 術後3日目以降 | 4単位 | - |
|  | 300以上～349以下 | 術後1日目以降 | 6単位 | - |
|  | 350以上～399以下 | 術後1日目以降 | 8単位 | - |
|  | 400以上 | 術後1日目以降 | 10単位 | - |
| 低血糖 | 69以下 | 術後1日目以降 | - | 20mL |

＜高血糖および低血糖に対するレスキュー治療の根拠＞

対象が非糖尿病患者であること、主要評価項目が術後2日目までのTIRの平均値であることから、術後２日目までの期間に限り、血糖値が201mg/dL以上299mg/dL以下の場合においては、即効型インスリンの非介入を許容することとした。一方、術後2日目までの血糖値が69mg/dL以下、もしくは300mg/dL以上の場合は、血糖コントロール非介入による患者の不利益が無視できないと判断し、各々、グルコースや即効型インスリン投与による介入を行うこととした。

## 6.2. プロトコル治療完了・中止・変更基準

### 6.2.1. プロトコル治療完了の定義

プロトコル治療の開始はFreeStyleリブレProの貼付として、明治メイン^、^グルセルナ^®^-REXまたはその他の栄養剤の投与終了し、FreeStyleリブレProを回収した時点を終了とする。

術後8日目まで 明治メイン、グルセルナ^®^-REXまたはその他の栄養剤を投与し、かつ、術後9日目までFreeStyleリブレProを貼付しつづけた場合、プロトコル治療完了とする。

### 6.2.2. プロトコル治療中止の基準

以下のいずれかの場合、プロトコル治療を中止する。なお、プロトコル治療中止日は、死亡による中止の場合は死亡日、それ以外の場合は担当医師がプロトコル治療中止と判断した日とする。

術後経過に多大な影響を及ぼすような、術中偶発症が生じた場合。

有害事象などで試験の継続が困難になった場合。

1. 患者がプロトコル治療の中止を申し出た場合。
2. プロトコル治療中の死亡。
3. 研究責任医師または分担医師の判断により中止を必要と判断した場合。
4. ICU在室期間中(術後4日以内)に、センサーが剝がれ、データの欠損が明らかとなった場合。
5. 術後2日目までに、インスリン投与が必要とされる高血糖状態 (300mg/dL以上)となった場合。
6. 術後2日目までに、糖投与が必要な低血糖状態 (69mg/dL以下)となった場合。
7. FreeStyleリブレProセンサーのシールが原因と考えられる皮膚トラブルが生じた場合。
8. MRI検査のためにFreeStyleリブレProセンサーのシールをはがす必要性が生じた場合。
9. その他、プロトコル違反、登録後の術式、治療方針変更などプロトコルからの重大な逸脱が判明した場合。

### 6.2.3. プロトコル治療の変更基準

栄養剤投与による有害事象(7.1.1参照)により、対照群・介入群双方に、プロトコル治療以外の別の栄養剤（既存の栄養剤の中から，責任医師もしくは分担医師が適切と判断したもの）への変更種類変更、もしくは栄養剤の投与量減量を要する可能性がある。Grade3以上(CTCAE ver.5.0)の症状を認めた場合は、対照群・介入群ともに、責任医師もしくは分担医師により適切と判断されるプロトコル治療以外の栄養剤への変更、もしくはプロトコル治療の栄養剤は変更せず、前日比として50%以上の投与量減量を行う。

## 6.3. 併用療法・支持療法

### 6.3.1. 高血糖・低血糖に対する許容される併用・支持療法

高血糖および低血糖に対するレスキュー治療（6.1.3血糖管理）以外は認めない。

### 6.3.2. その他の許容される併用・支持療法

整腸剤や止痢剤、輸液も含め日常診療で使用する薬剤投与は許容される。

### 6.3.2. 許容されない併用療法・支持療法

許容されない併用療法、支持療法は特にない。

# 7. 予期される有害事象

## 7.1. 予期される有害事象・不具合

### 7.1.1. 経腸栄養剤投与(明治メイン、グルセルナ^®^-REXともに)に伴う有害事象

1. 誤嚥
2. 下痢
3. 便秘
4. 腹部膨満感
5. 腹痛
6. 悪心
7. 嘔吐
8. 小腸閉塞
9. 高血糖
10. 低血糖

### 7.1.2. 食道切除術に伴う有害事象

1. 術後肺炎
2. 反回神経麻痺
3. 縫合不全
4. 乳び胸
5. 出血
6. 吻合部狭窄
7. 胃内容排泄遅延
8. 膵液瘻
9. 創感染
10. 腹腔内膿瘍
11. 胸水貯留

### 7.1.3. FreeStyleリブレPro使用に伴う重大な有害事象/有害事象

センサーの装着部位における重大な有害事象

1. 局所感染

センサーの装着部位における有害事象

1. 出血
2. 紅斑
3. 浮腫
4. 発疹
5. かゆみ
6. 紫斑
7. 硬化
8. 感染
9. 疼痛
10. 炎症

### 7.1.4. FreeStyleリブレPro使用に伴う不具合

1. ソフトウェアの動作不良/データ読み取り時あるいは情報表示時におけるエラー等（Reader）
2. 電源が入らない/電気系統のトラブル等（Reader）
3. 接着不良（センサー）

## 7.2. 有害事象/有害反応の評価

評価について、経腸栄養に伴う有害事象はCTCAE ver.5.0を用いて行い、食道切除術に伴う有害事象については、Clavien Dindo分類を用いて行う。

## 7.3. 有害事象観察期間

明治メインおよびグルセルナ^®^-REX投与終了後 から 30日間、有害事象の観察を行う。

## 7.4. 因果関係の判定

発現した有害事象と、経腸栄養剤投与及びFreeStyleリブレPro使用との因果関係は、「経腸栄養剤投与並びにFreeStyleリブレPro使用から有害事象発生までの時間的関連性に妥当性があり、かつ経腸栄養剤投与並びにFreeStyleリブレPro使用以外から原因を説明できないもの」を、関連ありと判定する。

1. 有害事象とプロトコル治療との因果関係の判定に際しては、”definite、probable、possible、unlikely、not related”の5カテゴリーに分類する。それぞれ”definite、probable、possible”のいずれかと判断された場合は「因果関係あり」とし、”unlikely、not related”のいずれかと判断された場合は「因果関係なし」と定義する。
2. 有害事象のGradeにより「10.1.緊急報告義務のある有害事象」に該当する場合には、「10.2.研究責任医師の報告義務と報告手順」に従い、研究事務局へ報告する。

表7.2.3有害事象と治療との因果関係の判断基準

| 因果関係 | 判定 | 判定の考え方 |
| --- | --- | --- |
| あり | definite | 有害事象がプロトコル治療により生じた、または重症化したことが明らかで、原病増悪や他の要因による可能性がほとんどないと判断 |
|  | probable | 有害事象が、原病増悪や他の要因により生じた、または重症化した可能性はありそうになく、プロトコル治療による可能性が高いと判断 |
|  | possible | 有害事象がどちらかといえばプロトコル治療により生じた、または重症化したと考える方がもっともらしく、原病増悪や他の要因による可能性は低いと判断 |
| なし | unlikely | 有害事象が、プロトコル治療により生じた、または重症化したと考えるよりも、どちらかといえば原病増悪や他の要因によると考える方がもっともらしいと判断 |
|  | not related | 有害事象が、原病増悪や他の要因により生じた、または重症化したことが明らかで、プロトコル治療による可能性がほとんどないと判断 |

# 8. 評価項目

## 8.1. 登録前(手術日より28日前以内)の評価項目

1. 患者背景

　　性別、生年月日、身長、体重、血圧、脈拍、既往歴、合併症、アレルギー歴、使用している薬剤

1. 自覚症状、他覚所見、全身状態 PS （ECOG）
2. 末梢血算:　白血球数、好中球数(ANC:桿状核球+分節核球)、リンパ球数、ヘモグロビン、血小板
3. 血液生化学:　総蛋白、アルブミン、総コレステロール、総ビリルビン、AST(GOT)、

ALT(GPT)、BUN、クレアチニン、eGFR、LDH、カルシウム、ナトリウム、カリウム、CRP、FBS(空腹時血糖)、HbA1c、プレアルブミン

1. 尿検査(随時尿): 尿蛋白定性、尿糖定性、尿比重、尿pH、尿ウロビリノーゲン、尿ビリルビン
2. X線、（造影）CT検査
3. 心電図検査

## 8.2. 手術当日の評価項目

1. 手術情報
2. 割付
3. FreeStyleリブレPro貼付
4. 24時間持続血糖
5. 生存確認

## 8.3. 手術後1日目の評価項目

1. FreeStyleリブレPro貼付継続
2. 24時間持続血糖
3. 血液ガスによる1日4回の血糖測定
4. 経腸栄養剤（明治メインおよびグルセルナ^®^-REX）400ｍLの持続投与
5. 必要に応じて、高血糖や低血糖に対してインスリンのレスキュー
6. 術後感染性合併症の有無
7. その他の合併症の有無
8. 有害事象
9. 生死の確認

## 8.4. 手術後2～3日目の評価項目

1. FreeStyleリブレPro貼付継続
2. 24時間持続血糖
3. 血液ガスによる1日4回の血糖測定
4. 経腸栄養剤（明治メインおよびグルセルナ^®^-REX）の持続投与（術後2日目；800ｍL、術後3日目：1200ｍL）
5. 必要に応じて、高血糖や低血糖に対してインスリンのレスキュー
6. 術後感染性合併症の有無
7. その他の合併症の有無
8. 有害事象
9. 生死の確認

## 8.5. 手術後4～8日目の評価項目

1. FreeStyleリブレPro貼付継続
2. 24時間持続血糖
3. デキスターによる1日4回の血糖測定
4. 経腸栄養剤（明治メインおよびグルセルナ^®^-REX）の持続投与（術後4日目以降；1600ｍL）

患者の状態（有害事象など）により投与量の変更およひ経腸栄養剤の変更可（6.2.3.参照）

1. 必要に応じて、高血糖や低血糖に対してインスリンのレスキュー
2. 術後感染性合併症の有無
3. その他の合併症の有無
4. 有害事象
5. 生死の確認

## 8.6. 手術後9日目の評価項目

1. FreeStyleリブレProを外す。(術後1～8日目の血糖データの回収)
2. 経腸栄養剤：ハイネイーゲルへの投与変更（患者の状態により変更時期の変更可）

ただし、術後9日目以降も明治メインおよびグルセルナ^®^-REXを投与する場合、投与終了するまで

## 8.7. 退院時^※（^後観察）の評価項目：許容範囲　退院日±7日

1. 全身状態:PS
2. 末梢血算:白血球数、好中球数(ANC:桿状核球+分節核球)、リンパ球数、ヘモグロビン、血小板
3. 生化学検査:総蛋白、総蛋白、アルブミン、総コレステロール、総ビリルビン、AST(GOT)、ALT(GPT)、BUN、クレアチニン、eGFR、LDH、カルシウム、ナトリウム、カリウム、CRP、FBS(空腹時血糖)、HbA1c、プレアルブミン
4. 術後感染性合併症の有無
5. その他の合併症の有無
6. 有害事象
7. 生死の確認

※退院が術後14日を超える場合、術後14日目を退院日として検査・評価を実施すること。

## 8.8. 明治メインおよびグルセルナ^®^-REX の最終投与30日後（後観察）の評価項目：許容範囲+14日

1. 術後感染性合併症の有無
2. その他の合併症の有無
3. 有害事象
4. 生死の確認

|  | スクリーニング | プロトコル治療期間 | | | | | | 中止^※7^ | 後観察期間 | |
| --- | --- | --- | --- | --- | --- | --- | --- | --- | --- | --- |
|  |  | 手術 | ICU | | | 一般病棟^※6^ | |  |  |  |
| 期間 | Day-28～-1 | Day0 | Day1 | Day2 | Day3 | Day4～8 | Day9 | - | 退院^※8^ | 最終  投与  30日後^※9^ |
| 許容範囲 |  |  |  |  |  |  |  |  | ±7日 | +14日 |
| 同意 | ● |  |  |  |  |  |  |  |  |  |
| 患者背景 | ● |  |  |  |  |  |  |  |  |  |
| 自覚症状 | ● |  |  |  |  |  |  |  |  |  |
| 他覚所見 | ● |  |  |  |  |  |  |  |  |  |
| 全身状態 PS （ECOG） | ● |  |  |  |  |  |  |  | ● |  |
| 末梢血算 | ● |  |  |  |  |  |  |  | ● |  |
| 血液生化学 | ● |  |  |  |  |  |  |  | ● |  |
| 尿検査 | ● |  |  |  |  |  |  |  |  |  |
| X線 | ● |  |  |  |  |  |  |  |  |  |
| CT検査 | ● |  |  |  |  |  |  |  |  |  |
| 心電図検査 | ● |  |  |  |  |  |  |  |  |  |
| 割付 |  | ● |  |  |  |  |  |  |  |  |
| 手術情報 |  | ● |  |  |  |  |  |  |  |  |
| FreeStyleリブレPro^※1^ |  | ● | ● | ● | ● | ● | ● |  |  |  |
| 24時間持続血糖 |  | ● | ● | ● | ● | ● |  |  |  |  |
| 血糖  （血液ガスもしくはデキスター） |  |  | ● | ● | ● | ● |  |  |  |  |
| 経腸栄養剤の  投与^※2^ |  |  | 400mL | 800ｍL | 1200ｍL | 1600mL |  | (●) |  |  |
| レスキュー |  |  | ● | ● | ● | ● | ● | ● |  |  |
| 術後  感染性合併症^※3^ |  |  | ● | ● | ● | ● | ● | ● | ● | ● |
| その他の合併症^※4^ |  |  | ● | ● | ● | ● | ● | ● | ● | ● |
| 有害事象^※5^ |  |  | ● | ● | ● | ● | ● | ● | ● | ● |
| 生存 |  | ● | ● | ● | ● | ● | ● | ● | ● | ● |

## 8.9. スタディカレンダー

※1：FreeStyleリブレProは、術後直後病棟に帰室後、研究分担医師により患者の上腕外側に付与する。

術後9日目に、術後8日目の経腸栄養剤等を投与終了後に FreeStyleリブレProを外す。

※2：明治メインおよびグルセルナ^®^-REXの投与開始や投与量については患者の状況に応じて変更可。

中止症例を含め、術後1～8日目の経腸栄養剤等を投与した際の情報は収集する。

※3：術後感染性合併症の評価は Clavien Dindo分類を用いる。

※4：その他の合併症の評価は、 Clavien Dindo分類を用いる。

※5：有害事象の評価は、 CTCAE ver.5.0を用いて評価する。

※6：ICU一般病棟への移動は、患者の状態により実施する。（術後4日目は問わない）

※7：中止決定時に FreeStyleリブレProを外す。

※8：退院が術後14日を超える場合、術後14日目を退院日として検査・評価を実施する。

※9：退院時検査と最終投与３０日後の検査は同日に実施も可。

# 9. 有害事象の報告

有害事象の報告は、「臨床研究法」（平成29 年法律第16 号）、「臨床研究法施行規則」（平成30 年厚生労働省令第17 号）並びにその関連通知に基づく本章の規定に従う。「重篤な有害事象（臨床研究法上の「疾病等」）が生じた場合、研究分担医師は研究責任医師/研究事務局に報告する。

1. 有害事象の報告に用いる重症度基準は「7.2. 有害事象／有害反応の評価」に記載の方法を用いる。
2. 本試験との因果関係が否定できない有害事象を疾病等という。
3. プロトコール治療開始日以降、プロトコール治療完了日から30日までに生じた疾病等を報告する。
4. 疾病等報告に際しては、厚生労働省ウェブサイトにて入手した、最新版の書式を用いること。

[**http://www.mhlw.go.jp/stf/seisakunitsuite/bunya/0000163417.html**](http://www.mhlw.go.jp/stf/seisakunitsuite/bunya/0000163417.html)

## 9.1. 報告義務のある有害事象

表9.1）に記載のいずれかに該当する、本試験との因果関係が否定できない有害事象（疾病等）を、報告の対象とする。

表9.1）［疾病等報告］報告義務と報告期限一覧

・「予期可能：未知」とは「7.予期される有害事象」に記載されていないものを指す。

・死亡：登録後、プロトコール治療開始前に発生したすべての死亡

プロトコール治療中または治療日から14日以内のすべての死亡

・死亡につながるおそれ：プロトコール治療中または治療日から14日以内に発生した、CTCAE-Grade 4 の事象

・治療のための入院または入院期間の延長^※^：プロトコール治療中または治療日から14日以内に発生した事象

^※^「入院または入院期間の延長」については、有害事象の治療のために24 時間以上の入院または入院期間の

延長が医学的に必要となるもののみを指し、次のような場合は報告対象外とする。

1. 有害事象が消失または軽快しているものの経過観察のために行われた入院または入院期間の延長
2. 遠隔地から受診する場合等、患者の負担を軽減する目的の入院または入院期間の延長
3. その他、医学的には必要のない入院または入院期間の延長

・研究責任医師が事象を知り得た日を起点(Day0)とする。

## 9.2. 有害事象又は疾病等が発生した場合の報告義務と報告手順

### 9.2.1. 本試験に従事する者の責務

本試験に従事する者は、因果関係が否定できない有害事象（疾病等）を知り得た場合、当該事象に関する情報を研究事務局へ速やかに報告する。

ただし、当該有害事象が原疾患の悪化（再発）である場合、社会的入院である場合、本試験の参加前から決定していた入院である場合は、報告は不要とする。

有害事象の発生の要因等が明らかではない場合であっても、それまでに判明している範囲で第1報として報告を行う。その後、可能な限り速やかに、当該有害事象に関してその時点までに把握できている情報を記載し、続報として研究事務局を通して研究責任医師に報告を行う。因果関係が否定できると判断していた場合でも、後に疾病等に該当すると判断した場合は、その時点で研究事務局に報告する。

### 9.2.2. 研究事務局の責務

研究事務局は、本試験に従事する者から、因果関係が否定できない有害事象（疾病等）の発現の報告を受けた場合には、速やかに施設管理者および研究責任医師へ文書による報告を行う。

### 9.2.3. 研究責任医師および研究事務局の責務

研究責任医師および研究事務局は、研究事務局に報告があった疾病等に関し、報告内容の緊急性、重要性、影響の程度などを判断し、必要に応じて登録の一時停止や周知事項の緊急連絡などの対策を講ずる。

### 9.2.4. 効果・安全性評価委員会への報告

研究責任医師は、報告義務がある有害事象について効果・安全性評価委員会の意見が必要と判断した場合、速やかに効果・安全性評価委員会に報告し、研究責任医師の見解および有害事象への対応に関する妥当性について意見を求める。

### 9.2.5. 効果・安全性評価委員会の責務

効果・安全性評価委員会は、報告があった有害事象の内容を検討し、試験継続の可否や、プロトコル改訂の要否などを含む対応について、研究責任医師・研究事務局に文書で勧告する。

### 9.2.6. 認定臨床研究審査委員会および厚生労働大臣への報告

「表9.1）［疾病等報告］報告義務と報告期限一覧」を参照の上、報告が必要な疾病等と判断された場合は、定められた期間内に報告を行う。

**・認定臨床研究審査委員会への報告**

臨床研究法で定める「医薬品疾病等報告書(統一書式8)」及び「詳細記載用書式」を用いて、認定臨床研究審査委員会（Certified Reviewed Board：CRB）への報告を行う。

**・厚生労働大臣への報告**

臨床研究等提出・公開システム^※1^（Japan Registry Clinical Trials：jRCT

＜https://jrct.niph.go.jp/＞）の「疾病等報告」へ入力（［別紙様式2－1］を作成）し、「PMDA^※2^

にメール送信」することで、厚生労働大臣への報告とする。

^※1^臨床研究法施行規則第24条第1項に規定する厚生労働省が整備するデータベース

^※2^Pharmaceuticals and Medical Devices Agency：独立行政法人医薬品医療機器総合機構

### 9.2.7. 定期報告

研究責任医師は、臨床研究に関して発生した全ての疾病等について、年に１回実施する定期報告において、CRB及び実施医療機関の管理者、および厚生労働大臣に疾病等の発生状況を報告する。

# 10. 効果判定とエンドポイントの定義

## 10.1. 評価項目

主要評価項目：CGMで測定した術後2日目までのTIRの平均値

副次評価項目：1. 入院期間中の感染性合併症の発生割合

2. 術後30日以内の感染性合併症の発生割合

3. 入院期間中の全合併症の発生割合

4. 有害事象発生割合

5. CGMで測定した全測定期間（術後1-8日目）におけるTARの平均値

6. CGMで測定した術後8日目までの各日毎のTAR

7. CGMで測定した全測定期間（術後1-8日目）におけるAUCの平均値

8. CGMで測定した術後8日目までの各日毎のAUC

9. CGMで測定した全測定期間(術後1-8日目)におけるTIRの平均値

10. CGMで測定した術後8日目までの各日毎のTIR

11.入院期間中の栄養学的指標の入院時に対する変化率

（血清のアルブミン値、プレアルブミン値、総蛋白値）

12. 高血糖基準(≧300mg/dL)に対する血糖コントロール介入症例数

13. 術後3日目以降の経腸栄養剤変更症例数

14. 術後3日目以降、前日比で50%以上の経腸栄養剤減量を要した症例数

・定義（2.4.1参照）

TIR：24時間の血糖値の経時的測定のうち、血糖値が70～

180mg/dLの治療域(target range)内にある時間の割合。

TAR：24時間の血糖値の経時的測定のうち、血糖≧180mg/dL以上の高血糖域の時

間割合。

AUC：24時間の血糖値の経時的測定により得られる血糖変動曲線における、血糖≧180mg/dL以上の

曲線下面積。

# 11. 統計的事項

統計解析の詳細については、別途作成する統計解析計画書に記載する。プロトコルで計画した解析のうち、変更があった解析については、変更理由を統計解析計画書に記載する。

## 11.1. 解析対象集団の定義

本研究試験における解析対象集団の定義は以下の通りとする。各症例の取り扱いは、データ固定前に研究責任医師、研究事務局、統計解析責任者およびデータセンターが協議を行い決定する。

主たる解析を含む有効性に関する解析はFull Analysis Set （FAS）を対象とし、安全性に関する解析は全治療例を対象として解析を行う。主たる解析は感度分析としてPer Protocol Set（PPS）を対象にした解析も行う。

**・全登録例**

登録された患者すべてを「全登録例」とする。

**・Full Analysis Set（FAS）**

全登録例から、重大な選択基準違反例除外基準違反例、プロトコール治療未実施症例、有効性に関するデータがひとつもない症例、研究途中に同意を撤回かつすべてのデータの使用を拒否した症例を除く集団を「FAS」とする。

**・PPS（Per Protocol Set）**

FASから、術後2日目までにプロトコル治療を中止した症例や重大なプロトコル違反例を除く集団を「PPS」とする。

**・全治療例**

プロトコル治療の一部または全部が施行された症例を「全治療例」とする。

## 11.２. データ欠測の扱い

原則として欠測は補完しない。ただし、必要に応じて感度分析として欠測を補完した解析を実施する。欠測値の補完に関する詳細は統計解析計画書に記載する。センサーがICU在室期間中に何らかの理由により剥がれ、データの収集に欠測が出た場合、既報告の推奨に基づき、センサーを貼付した時点から、剝がれてしまった時点までに測定し得たデータが、測定予定日数の70%以上(6.3日以上)であった症例を解析対象とした解析も感度分析として実施する[8, 12]。

## 11.３. 主たる解析

本試験では、患者登録終了後に行なう主要評価項目であるCGMで測定した術後2日目までのTime in range (TIR)の平均値の解析を主たる解析とする。共分散分析により割付因子で調整した主要評価項目における2群の平均値の差（介入群-対照群）の平均値（最小二乗平均）とその95%信頼区間及び標準偏差を算出する。また、参考として2群の平均の差=0とする帰無仮説に対する*P*値や各群の主要評価項目の平均値（最小二乗平均）とその95%信頼区間及び標準偏差、割付因子で調整しない結果も算出する。術後2日目までのTIRの平均値とは48時間のうち血糖値が70～180mg/dLの治療域（target range）内にある時間の割合である。

## 11.4. 副次評価項目（有効性）

以下の項目に対して、各群と群間差（介入群-対照群）の平均値とその95%信頼区間及び標準偏差を算出する。必要に応じて2群間の比較としてWelchのt検定による*P*値を算出する。

・CGMで測定した全測定期間（術後1-8日目）におけるTARの平均値

・CGMで測定した術後8日目までの各日毎のTAR

・CGMで測定した測定期間（術後1-8日目）におけるAUCの平均値

・CGMで測定した術後8日目までの各日毎のAUC

・CGMで測定した全測定期間(術後1-8日目)におけるTIRの平均値

・CGMで測定した術後8日目までの各日毎のTIR

## 11.5. 副次評価項目（安全性）

以下の項目に対して、各群の頻度、割合とその95%信頼区間を算出する。95%信頼区間は Clopper & Pearsonの正確な信頼区間とする。必要に応じて2群間の比較としてカイ二乗検定による*P*値を算出する。

有害事象は発現例数、発現件数、発現例数割合と95%信頼区間を算出する。95%信頼区間は Clopper & Pearsonの正確な信頼区間とする。Grade別、因果関係別の集計も行う。また、すべての有害事象について一覧表を作成する。

・入院期間中の感染性合併症の発現割合

・術後30日以内の感染性合併症の発現割合

・入院期間中の全合併症の発現割合

・有害事象発現割合

・高血糖(≧300mg/dL)に対する血糖コントロール介入症例数・術後3日目以降に経腸栄養剤変更症例数

・術後3日目以降、前日比で50%以上の経腸栄養剤減量を要した症例数

以下の項目に対して、全ての測定時点に対して各群の中央値と四分位範囲を算出する。また、登録前の測定値を基準にした変化量の中央値と四分位範囲を算出する。必要に応じて平均値と標準偏差、Wilcoxon検定による *P*値を算出する。

・入院期間中の栄養学的指標（血清のアルブミン値、プレアルブミン値、総蛋白値）

## 11.6. 予定登録数

１）予定症例数: 50例 (対照群25例、介入群25例)。

２）目標症例数の算出根拠

　 本研究は、統計学的仮説に基づく症例数の設定は困難である (グルセルナ^®^-REXの血糖降下作用を報告した

論文がないため)。脱落を見込み、48例を対象にTIRの群間差の標準偏差を8.5%とすると信頼区間幅±5%程度でTIRの群間差の平均値を推定できる。

現在がん研有明病院食道外科では、食道癌に対する切除・再建手術を年間110例程度行っている。除外症例も考慮し、実施承認後～1.5年以内に症例登録が完了する見込みである。

## 11.7. 中間解析

本試験では中間解析は行わない。

## 11.8. 有意水準及び多重性

　本治験のすべての解析の有意水準は両側5%、信頼区間の信頼係数は両側95%とする。有効性評価、安全性評価ともに、評価項目間、及び時点間の多重性の調整は行わない。

## 11.9. 最終解析

追跡期間終了後、最終調査によりデータを確定した後に、すべてのエンドポイントに対する解析を行なう。

# 12. 研究全体の中止

## 12.1. 研究全体の中止基準

- 症例登録の遅れ、研究計画書逸脱の頻発などの理由により、研究の完遂が困難と判断された場合
- ベネフィットに対するリスクの割合が認容できないと判断された場合（本研究の安全性の知見、中間解析をおこなう場合はその結果等）
- 論文や学会発表など、本研究以外から得られた関連情報を評価した結果、本研究の安全性に問題が

あると判断された場合、又は研究継続の意義がなくなったと判断された場合

## 12.2. 臨床研究の中止の手続き

1. 研究責任医師は「12.1研究全体の中止基準」に基づき臨床研究を中止する場合、研究分担医師とともに当該臨床研究の対象者に適切な措置を講じる。なお、必要に応じて研究対象者の措置に伴う研究終了時期やその方法について、CRBの意見を聴く。
2. 研究責任医師は、臨床研究を中止した日から10日以内に、その旨を、中止通知書（統一書式11）

を用いて、当該臨床研究の実施計画に記載されているCRBに通知するとともに、jRCTにて［省令様式第4］を作成し、厚生労働大臣へ報告する。

1. 中止通知書（統一書式11）を提出した場合であっても、その後、臨床研究が終了するまでの間におい

て、臨床研究の進捗状況に関する事項の変更に該当する場合には、実施計画の変更の届出を行う。

1. 中止通知書（統一書式11）を提出した場合であっても、臨床研究が終了するまでの間においては、

疾病等報告、定期報告等を行う。

1. 中止後の臨床研究の終了の時期は、研究対象者の措置を終え、研究が終了するときをいう。
2. 臨床研究を中止した場合であって、中止通知書（統一書式11）を提出し研究対象者の措置を終えた

場合においては、中止した日又は全ての評価項目に係るデータの収集を行うための期間が終了した日のいずれか遅い日から原則1年以内に総括報告書を提出する。

# 13. 倫理的事項

## 13.1. 患者の保護

1. 本試験に関係するすべての研究者は、下記に従い、本試験を実施する。
2. 「人を対象とする生命科学・医学系研究に関する倫理指針^※1^」（令和3年3月23日(令和5年3月27日一部改訂)文部科学省、厚生労働省、経済産業省）

^※1^https://www.mhlw.go.jp/content/001077424.pdf

1. 「ヘルシンキ宣言^※2^」（2013年ブラジル、フォルタレザ）

^※2^http://dl.med.or.jp/dl-med/wma/helsinki2013j.pdf

1. 「臨床研究法^※3^」（平成29年法律第16号）「臨床研究法施行規則」（平成30年厚生労働省令第17号）並びに「関連通知」

^※3^<http://www.mhlw.go.jp/stf/seisakunitsuite/bunya/0000163417.html>

1. 研究責任医師は、本試験の開始に先立ち、本試験の実施について、CRBの承認を得た上で、厚生労働大臣への実施計画^※^の提出（jRCTにて実施計画の登録、研究情報の公表）を行い、また実施医療機関の管理者の研究実施許可を得なければならない。

^※^臨床研究法施行規則第39 条第1 項に規定する省令様式第一

## 13.2. インフォームドコンセント

### 13.2.1. 患者への説明

患者登録に先立って、研究責任医師または研究分担医師はCRBの承認が得られた説明文書を患者本人に渡し、以下の内容を口頭で詳しく説明する。

患者への説明事項：

1. 本試験が臨床試験であること
2. 本試験のデザインおよび根拠（rationale：意義、必要性、目的など）
3. プロトコル治療の内容
4. プロトコル治療により期待される効果
5. 予期される有害事象、合併症、後遺症とその対処法について
6. 研究に係る費用

費用負担と補償治療にかかる費用は保険制度でまかなわれること、健康被害が生じた場合の補償は一般診療での対処に準ずることなど、一般診療と同様であること

1. 予想される利益と可能性のある不利益について

試験に参加することによって享受できると思われる利益と被る可能性のある不利益に関して

1. 同意拒否と同意撤回
   試験参加に先立っての同意拒否が自由であることや、いったん同意した後の撤回も自由であり、それにより不当な診療上の不利益を受けないこと
2. 人権保護
   氏名や個人情報は守秘されるための最大限の努力が払われること
3. 臨床研究に関わる利益相反
4. 研究成果の公表
    本臨床試験で得られた結果は学術論文、学会にて公表すること。その際にも公表内容には個人情報に関することは含まないこと
5. データの二次利用
    委員会が承認した場合に限り、個人識別情報とリンクしない形でデータを二次利用する（メタアナリシスなど）可能性があること
6. 知的財産権の帰属
    本研究から生じる知的財産権は公益財団法人がん研究会に帰属すること
7. 研究組織
8. 質問の自由
    担当医師の連絡先のみでなく、医療機関の研究責任者、試験の研究代表者（または研究事務局）の連絡先を文書で知らせ、試験や治療内容について自由に質問できること

### 13.2.2. 同意

研究責任医師・研究分担医師である担当医師により、患者に試験についての説明を行い、十分に考える時間を与え、患者が試験の内容をよく理解したことを確認した上で、試験への参加について依頼する。患者本人が試験参加に同意した場合、説明文書・同意文書に付表の同意書を用い、患者本人または代諾者による署名を得る。担当医師は、説明を行った医師名、説明を受け同意した患者名、同意を得た日付の記載が、同意書にあることを確認する。同意文書は 2 部作成し、1 部は患者本人に手渡し、1 部は研究責任医師が保管する。原本はカルテもしくは医療機関で定められた保管場所に保管する。

### 13.2.3. 同意撤回

- 試験参加の同意を得た後、患者本人から試験参加への同意を取り消す申し出があった場合、説明文・同意文書に付表の同意撤回書を用い、患者本人または代諾者による署名を得て、同意撤回とする。担当医師は、同意撤回をした患者名、同意撤回をした日付の記載が、同意撤回書にあることを確認する。同意撤回文書は 2 部作成し、1 部は患者本人に手渡し、1 部は研究責任医師が保管する。原本はカルテもしくは医療機関で定められた保管場所に保管する。
- 同意撤回とは、試験参加への同意の撤回を意味し、プロトコル治療継続の可否（下記①）とは区別する。同意の撤回が表明された場合には、下記②か③のいずれかを明確にし、速やかに研究事務局に連絡すること。
- 研究事務局は②同意撤回の場合、以降のプロトコルに従ったフォローアップの依頼を中止する。③の場合、全同意撤回であることが確認された時点で、当該患者のデータをデータベースから削除する。
- 当該患者のフォローアップの依頼の中止及び患者データ削除の手順は別途、手順書に定めることとし、それぞれの作業が完了したことを研究責任医師、研究事務局に報告する。
  - 1. 患者拒否：以降のプロトコル治療継続の拒否（フォローアップは続ける）
    2. 同意撤回：試験参加への同意を撤回し、以後のプロトコールに従った治療、フォローアップのすべてを不可とすること。同意撤回以前のデータの試験利用は可。
    3. 全同意撤回：試験参加への同意を撤回し、登録時の情報を含む試験参加時点からのすべてのデータの試験利用を不可にすること。

## 13.3. 個人情報

個人情報及び診療情報などのプライバシーに関する情報は個人の人格尊重の理念の下、厳重に保護され慎重に取り扱われるべきものと認識し、万全な管理対策を講じ、プライバシー保護に努める。

### 13.3.1. 従うポリシー、法令、規範

研究を行うにあたり、原則として、以下の法令、規範に従う。下記以外の法令、規範、ポリシーが適応となる場合は、加えて従うこととする。

1. 個人情報の保護に関する法律（平成15年法律第57号最終改正：平成27年9月9日法律第65号）
2. 人を対象とする生命科学・医学系研究に関する倫理指針
3. 臨床研究法・臨床研究法施行規則・関連通知
4. ヘルシンキ宣言（日本医師会訳）

### 13.3.2. 個人情報の保護と患者識別

個人情報保護のため、本試験では患者登録時に発行される登録番号を用いて匿名化する。イニシャルやカルテID 等のような特定の個人を識別できる情報は用いない。研究責任医師が厳重に保管・管理し、患者の個人情報の漏洩を防止する。また、研究の成果報告、発表の際には、匿名加工をした上で公開する。

### 13.3.3. 個人情報の利用目的と利用する項目、及び利用方法

- 本試験では、臨床研究の正しい結果を得るために患者個人を特定して調査を行うことを目的として、患者の個人情報を利用する。
- 患者の同定や照会のために利用する個人情報は、年齢、生年月日、性別とする。
- 本試験が利用する患者の個人情報等は、各種症例報告書 (Case Report Form：CRF)等に研究責任医師・研究分担医師が入力し、原則として電子的臨床検査情報収集 (Electronic Data Capture ：EDC)により収集する。
- 報告義務のある疾病等報告などは、電子メール、郵送、手渡しのいずれかの方法で研究事務局に提出することにより収集する。ただし、迅速な連絡が必要となる患者情報の連絡に限り、電話を利用する。その場合、電話連絡の内容は診療録へ記録を残すこととする。
- 研究事務局と医療機関の研究者間で電子メール等による問い合わせや報告のやり取りをする場合には、より匿名性の高い登録番号のみを用い、診療録番号やイニシャル等を用いてはならない。

## 13.4. 原資料

本試験で利用する臨床研究に関する原資料は、本試験の登録患者の、診療録（ワークシートなどを含む）、検査記録、診断に用いた画像、病理診断書、効果判定に用いた画像、同意文書を含む診断・治療に用いた全ての記録を指す（医療機関の規程で電子化した紙文書を原本とすることが認められている場合は電子化文書を原資料と扱う）。これらは、モニタリング、監査（必要に応じて実施）、並びにCRB及び規制当局の調査の際に、直接閲覧に供する。

## 13.5. 試料・情報などの保管

本試験に関する登録患者の試料及び情報などは、「臨床研究法施行規則 （平成30 年厚生労働省令第17号）第53 条」に従って保管すること。参加施設における本試験に関する記録の保管期限、および原資料の保管期限は臨床研究が終了した日から5年間とする。期限を過ぎた後もできるだけ長期に保管することが推奨される。

## 13.6.補償について

本試験の参加に起因して研究対象者に健康被害が生じた場合、研究責任医師、または研究分担医師は、適切な治療及びその他必要な措置を行う。この場合の治療等は保険診療として行い、自己負担分の医療費を研究対象者が支払う。なお、本試験に起因する未知の健康被害が生じた場合に備えて臨床研究保険（賠償、補償金及び医療費・医療手当）に加入する。

## 13.7. 知的財産について

1. 本試験により得られた結果やデータの知的財産権は、公益財団法人がん研究会に帰属する。

## 13.8. プロトコルの遵守

1. 本試験に参加する研究者は、患者の安全と人権を損なわない限り、本プロトコルを遵守する。

## 13.9. CRBへの申請及び実施計画の届出

- 本試験への実施に際しては､本プロトコール及び患者への説明文書を用いて試験を実施することについて、CRBの承認、及び実施医療機関の管理者の研究実施許可を得なければならない。
- jRCTにて、厚生労働大臣へ実施計画を提出し、本試験の概要、進捗状況、主な結果などの研究情報の公表を行う。

**【公益財団法人がん研究会有明病院臨床研究審査委員会（CRB）】**

認定番号： CRB3220003
住所：東京都江東区有明 3 8 31
電話番号： 03 3520 0703
電子メールアドレス：ganken_crb jfcr.or.jp

# 14. 定期報告

臨床研究法施行規則の施行等について（平成30 年2 月28 日医政経発0228 第1 号 厚生労働省医政局経済課長・医政研発0228 第1 号 同研究開発振興課長通知）より、研究責任医師は、jRCTの初回公表日から起算して1年ごと、及び当該期間満了後2か月以内に、自らの所属する医療機関の管理者に報告した上で、CRBに臨床研究の継続の適否について意見を伺う。定期報告では、［定期報告書(統一書式5)］に下記 (1) から (5) の事項を簡潔に記載する。

1. 本試験に参加した対象者の数
2. 本試験に係る疾病等の発生状況及びその後の経過
3. 本試験に係る臨床研究施行規則又は研究計画書に対する不適合の発生状況及びその後の対応
4. 本試験の安全性及び科学的妥当性についての評価
5. 利益相反管理基準に定める医薬品等製造販売業者等との関与に関する事項

研究責任医師は、CRBの継続の適否についての結果を得た日から起算して1か月以内に、jRCTより厚生労働大臣へ報告（［定期報告書(通知別紙様式3)］を作成）し、公表する。

# 15. 臨床研究に関わる者の利益相反(COI)の管理について

1. 本試験は、がん研有明病院食道外科の自己資金により行う試験であり、特定の企業からの資金提供は受けず、利害の衝突は発生しない。
2. 本試験に関わるCOI は、「臨床研究法における利益相反管理ガイダンス」（平成30 年11 月30 日医政発1130 第17 号 厚生労働省医政局研究開発振興課長通知^※^）（以下、ガイダンス）に従い以下のように管理する。
   ^※^ <http://www.mhlw.go.jp/stf/seisakunitsuite/bunya/0000163417.html>COI 管理に用いる書式は、ガイダンスの最新版の書式を用いること。

・利益相反管理基準：様式A
・関係企業等報告書：様式B
・研究者利益相反自己申告書：様式C
・利益相反状況確認報告書：様式D
・利益相反管理計画：様式E

本試験の関連企業は試験計画の立案、データ取得、データ解析、解析結果のディスカッション、発表、論文作成には関与しない。本試験と関わりのある企業等との利益相反はない。

# 16. CRBに承認された書類の内容変更について

## 16.1 実施計画の変更

実施計画(省令様式第1)に変更が生じた場合には、CRBの承認を得た後、jRCTより厚生労働大臣へ「届出」をし、公表する。

## 16.2 プロトコールの内容変更について

本試験では、プロトコールの変更を改正・改訂の2 種類に分けて扱う。また、プロトコール内容の変更に該当しない補足説明の追加をメモランダムとして区別する。改訂と改正は下記を原則として判断する。

1. 改正
2. 試験に参加する患者の危険を増大させる可能性のある、または試験の主要評価項目 に実質的な影響を及ぼすプロトコールの部分的な変更。
3. 「改正」に相当すると判断された時点で、患者登録が継続されていた場合には、患者登録を一旦停止する。
4. 改訂
5. 試験に参加する患者の危惧を増大させる可能性がなく、かつ試験の主要評価項目 に実質的な影響を及ぼさないプロトコールの変更。
6. 原則として「改訂」の際には患者登録の一時停止は行わない。
7. メモランダム
8. プロトコール内容の変更ではなく、文面の解釈上のばらつきを減らす、または注意を喚起するなどの目的で、研究責任医師/研究事務局から試験の関係者に配布するプロトコールの補足説明。書式は問わない。
9. なお、プロトコール内容の変更には当たるが、登録患者のリスクを軽減するために、試験に関係する研究者間で速やかな情報共有が必要な場合には、改訂申請を前提としたメモランダムを発行する。

## 16.3 同意説明文書の内容変更及び、患者説明・再同意について

1. 変更が生じた場合には、CRBに報告し、承認を得る。
2. 説明文書及び同意文書を変更があった場合は、当該患者にその変更内容を説明した上で、臨床研究への参加継続について、自由意思による同意を文書により得る。

## 16.4 その他の承認が得られた書類について

1. 変更が生じた場合には、CRBに報告し、承認を得る。

# 17. 不適合の管理

## 17.1. 不適合

臨床研究法における不適合とは「臨床研究が臨床研究法施行規則または研究計画書に適合していない状態」を指し、「臨床研究法施行規則の施行等について（平成30 年2 月28 日）」において、「規則、研究計画書、手順書等の不遵守及び研究データの改ざん、ねつ造等」が事例として挙げられている。

## 17.2. 重大な不適合

重大な不適合とは、臨床研究の対象者の人権や安全性および研究の進捗や結果の信頼性に影響を及ぼすものをいう。「重大な不適合」の例を以下に示す。これらの重大な不適合に相当する可能性がある場合には、研究事務局は状況を把握次第、速やかにCRBに報告する。

**1）適格性に関する重大な不適合**

違反登録

・適格規準を満たさないと知りながら故意に（偽って）登録した

・必要なインフォームド・コンセントを行わずに患者登録をし、プロトコール治療を実施した

・適格性を判断するための原資料が確認できない（同意書の紛失も含む）

**2）プロトコール違反**

登録患者のリスク増大に影響がある違反、または試験結果の信頼性に影響を及ぼす違反

・重大な適格基準・除外基準違反

・患者の安全性を脅かす中止基準違反

・重大な併用禁止薬違反・禁止併用療法などの不遵守

・故意または系統的なプロトコール規定の不遵守など

**3）その他の重大な不適合**

・CRBの承認前または実施医療機関の管理者の承認前に研究を実施した

・試験継続意思に影響を及ぼす可能性がある情報を提供せずに試験を継続した

・研究不正（データのねつ造、データの改ざんなど）と判断されるもの

・個人情報の漏洩または人権侵害により登録患者への重大な影響が認められるもの

なお、重大な不適合には、研究対象者の緊急の危険を回避するため、その他医療上やむを得ない理由により研究計画書に従わなかったものについては含まない。

## 17.3. 不適合報告の手順

- - 1. 本試験に従事する者は、不適合があると知ったとき、所属する実施医療機関の研究責任医師に速やかに報告する。
    2. 研究責任医師は、当該不適合について速やかに所属する実施医療機関の管理者に報告するとともに、研究責任医師に通知する。
    3. 研究責任医師は、当該不適合が研究対象者の人権や安全性及び研究の進捗や結果の信頼性に影響を及ぼすものであることが判明した場合には、重大な不適合として、速やかにCRBに「重大な不適合報告書(統一書式7)」を提出し意見を求める。
    4. 当該重大な不適合に関する再発防止策を講じ、所属する医療機関の研究分担医師及び当該臨床研究に従事する者に周知するとともに、再発防止の徹底を図る。

# 18. モニタリングと監査

## 18.1. 定期モニタリング

本試験では、試験が安全かつプロトコルに従って実施されているか、データが正確に収集されているかを確認する目的で中央モニタリングを行う。中央モニタリングはモニタリング手順書に従い実施する。

## 18.2. 監査

監査は実施しない。

# 19. 研究全体の終了

## 19.1. 研究の終了

jRCTより厚生労働大臣へ報告（［総括報告書の概要(通知別紙様式3)］を作成）し、公表した日を当該臨床研究が終了した日とする。「臨床研究法施行規則（平成30年2月２８日　厚生労働省令第17号）施行通知2.（24）規則第２４条第1項関係」

## 19.2. 研究終了の手順

1. 研究責任医師は、研究計画書に記載した全ての評価項目に係るデータの収集を行うための期間が終了したとき、原則としてその日を起算日として、一年以内に総括報告書および総括報告書の概要を作成する。

総括報告書には少なくとも以下の項目を含めること。「臨床研究法施行規則（平成30年2月28日　厚生労働省令第17号）施行通知2.（25）規則第24条第2項関係」

1. 臨床研究の対象者の背景情報（年齢、性別等）
2. 臨床研究のデザイン応じた進行状況に関する情報（対象者数の推移等）
3. 疾病等発生状況のまとめ
4. 主要評価項目及び副次評価項目のデータ解析及び結果
5. 総括報告書及び総括報告書の概要を作成したのち、遅滞なく、CRBに提出し、当該委員会の意見を聴く。「臨床研究法施行規則（平成30年2月28日　厚生労働省令第17号）第24条第4項」

CRBへの提出する書類

1. 終了通知書(統一書式12)
2. 総括報告書
3. 終了届書(通知別紙様式3)（総括報告書の概要）
4. 研究責任医師は、当該CRBが意見を述べた日から起算して一か月以内に、総括報告書及び総括報告書の概要を管理者に報告するとともに、jRCTより厚生労働大臣へ報告（［総括報告書の概要(通知別紙様式3)］を作成）し、公表する。「臨床研究法施行規則（平成30年2月28日　厚生労働省令第17号）第24条第5項」

jRCTの公表に際しては、下記の書類を添付する。

1. 研究計画書の最終版
2. 説明同意文書の最終版
3. 統計解析計画書を作成した場合には、当該統計解析計画書

総括報告書の概要の公表について、当該研究成果を論文等で公表する場合においては、CRBに論文投稿中の旨報告した上で、当該論文等の公表後としても差し支えない。この場合であっても、jRCTでの届出・報告は期限内に行い、届出・報告時に公表時期について申し出ること。ただし、研究論文等が公表された場合は、直ちに総括報告書の概要こととし、届け出の際に未記入で提出した項目（「結果に関する最初の出版物での発表日」及び「結果と出版物にURL」）について、jRCTに記録した上で公表すること。臨床研究法施行規則（平成30年2月28日　厚生労働省令第17号）施行通知2.（27）規則第24条第4項関係」

## 19.3. 研究結果の扱い

### 19.3.1. 研究結果の公表

研究結果の主たる公表論文（primary endpointの結果を初めて公表する論文）は英文誌に投稿する。主たる公表論文の著者は、原則として研究事務局を筆頭（first author)とし、研究代表者をcorresponding authorとするが、貢献度の高かった担当医師の貢献などを加味し研究事務局が総合的に判断する。その他の学会発表や副論文は複数回に及ぶ可能性があり、同様に判断する。すべての共著者は投稿前に論文内容をreviewし、発表内容を承認した者のみとする。論文に関して、議論を行っても合意が得られない場合、研究代表者は、その研究者を共著者に含めないことができる。

### 19.3.2. データの二次利用

将来、更なる研究発展のための貴重なデータとして、研究期間終了後も本試験結果を利用する可能性がある。尚、本試験のデータを別の研究に用いる場合には、改めて研究計画書を倫理審査委員会に諮り、承認を受けた上で利用する。

# 20. 効果・安全性評価委員会

1. 本試験では、効果安全性評価委員会を設置する。
2. 本委員会は、研究責任医師と独立した機関として設立され、本試験とは独立した立場である3人以上の専門家による委員で構成される。

研究期間中は効果・安全性評価委員会による監視（有害事象報告、モニタリングレポート審査、プロトコル改訂審査など）を受ける。

# 21. 研究組織

## 21.1. 研究責任医師

公益財団法人がん研究会有明病院　食道外科　部長　渡邊　雅之

〒135-8550

東京都江東区有明3-8-31

TEL：03-3520-0111

FAX：03-3570-0343

E-mail：masayuki.watanabe@jfcr.or.jp

## 21.2. 研究事務局

公益財団法人がん研究会有明病院　食道外科　今村　裕

〒135-8550

東京都江東区有明3-8-31

TEL：03-3520-0111

FAX：03-3570-0343

E-mail：[yu.imamura@jfcr.or.jp](mailto:yu.imamura@jfcr.or.jp)

公益財団法人がん研究会有明病院　食道外科　寺山　仁祥

〒135-8550

東京都江東区有明3-8-31

TEL：03-3520-0111

FAX：03-3570-0343

E-mail：masayoshii.terayama@jfcr.or.jp

## 21.3. 研究分担者

北澤公 がん研究会有明病院　糖尿病・代謝・内分泌内科副部長

金森淳 がん研究会有明病院　食道外科　医長

岡村明彦 がん研究会有明病院　食道外科　医長

栗山健吾 がん研究会有明病院　食道外科　医員

高橋直規 がん研究会有明病院　食道外科　医員

田村直弘 がん研究会有明病院　食道外科　医員

石井美鈴 がん研究会有明病院　栄養科

高木久美 がん研究会有明病院　栄養科

松下亜由子 がん研究会有明病院　栄養科

## 21.4. 統計解析責任者

公益財団法人がん研究会有明病院　先進がん治療開発センター　企画戦略部　宮﨑　直己

〒135-8550

東京都江東区有明3-8-31

TEL：03-3520-0111

FAX：03-3570-0343

E-mail：naoki.miyazaki@jfcr.or.jp

## 21.5. モニタリング責任者

公益財団法人がん研究会有明病院　先進がん治療開発センター　企画戦略部　松井　美子

〒135-8550

東京都江東区有明3-8-31

TEL：03-3520-0111

FAX：03-3570-0343

E-mail：yoshiko.matsui@jfcr.or.jp

## 21.6. データセンター

公益財団法人がん研究会有明病院　先進がん治療開発センター　企画戦略部　松井　美子

〒135-8550

東京都江東区有明3-8-31

TEL：03-3520-0111

FAX：03-3570-0343

E-mail：yoshiko.matsui@jfcr.or.jp

## 21.7. 効果・安全性評価委員会

公益財団法人がん研究会有明病院　肝胆膵外科　伊藤 寛倫

〒135-8550

東京都江東区有明3-8-31

TEL：03-3520-0111

FAX：03-3570-0343

[hiromichi.ito@jfcr.or.jp](mailto:hiromichi.ito@jfcr.or.jp)

公益財団法人がん研究会有明病院　胃外科　入野 誠之

〒135-8550

東京都江東区有明3-8-31

TEL：03-3520-0111

FAX：03-3570-0343

[tomoyuki.irino@jfcr.or.jp](mailto:tomoyuki.irino@jfcr.or.jp)

公益財団法人がん研究会有明病院　大腸外科　松井 信平

〒135-8550

東京都江東区有明3-8-31

TEL：03-3520-0111

FAX：03-3570-0343

[shimpei.mtsui@jfcr.or.jp](mailto:shimpei.mtsui@jfcr.or.jp)

# 22. 参考文献

1. **“がんの統計2021.”**2021. National Cancer Center Japan. <https://ganjoho.jp/data/reg_stat/statistics/brochure/2021/cancer_statistics_2021.pdf>
2. Kataoka K, Takeuchi H, Mizusawa J, Igaki H, Ozawa S, Abe T, et al. Prognostic Impact of Postoperative Morbidity After Esophagectomy for Esophageal Cancer: Exploratory Analysis of JCOG9907. Ann Surg. 2017;265(6):1152-1157.
3. May AK, Kauffmann RM, Collier BR. The place for glycemic control in the surgical patient. SurgInfect (Larchmt). 2011;12:405-18
4. Hori S, Imamura Y, Watanabe M, et al. Early postoperative hyperglycemia as a predictor of postoperative infectious complications and overall survival in non-diabetic patients with esophageal cancer. J Gatrointest Surg (Accepted).
5. Riddle MC, Gerstein HC, Cefalu WT. Maturation of CGM and Glycemic Measurements Beyond HbA(1c)-A Turning Point in Research and Clinical Decisions. Diabetes Care. 2017;40(12):1611-3.
6. Laffel LM, Kanapka LG, Beck RW, Bergamo K, Clements MA, Criego A, et al. Effect of Continuous Glucose Monitoring on Glycemic Control in Adolescents and Young Adults With Type 1 Diabetes: A Randomized Clinical Trial. Jama. 2020;323(23):2388-96.
7. Beck RW, Riddlesworth TD, Ruedy K, Ahmann A, Haller S, Kruger D, et al. Continuous Glucose Monitoring Versus Usual Care in Patients With Type 2 Diabetes Receiving Multiple Daily Insulin Injections: A Randomized Trial. Ann Intern Med. 2017;167(6):365-74.
8. Battelino T, Danne T, Bergenstal RM, et al. Clinical Targets for Continuous Glucose Monitoring Data Interpretation: Recommendations From the International Consensus on Time in Range. Diabetes Care. 2019;42(8):1593-603.
9. Beck RW, Bergenstal RM, Cheng P, et al. The Relationships Between Time in Range, Hyperglycemia Metrics, and HbA1c. J Diabetes Sci Technol. 2019;13(4):614-26.
10. Le Floch JP, Escuyer P, Baudin E, Baudon D, Perlemuter L. Blood glucose area under the curve. Methodological aspects. Diabetes Care. 1990;13(2):172-5.
11. Siegelaar SE, Holleman F, Hoekstra JB, DeVries JH. Glucose variability; does it matter? Endocr Rev. 2010;31(2):171-82.
12. Battelino T, Alexander CM, Amiel SA, Arreaza-Rubin G, Beck RW, Bergenstal RM, et al. Continuous glucose monitoring and metrics for clinical trials: an international consensus statement. Lancet Diabetes Endocrinol. 2023;11(1):42-57.
